# Absolute and relative facial selectivities in organocatalytic asymmetric chlorocyclization reactions†
†Electronic supplementary information (ESI) available. See DOI: 10.1039/c7sc04430e


**DOI:** 10.1039/c7sc04430e

**Published:** 2018-01-02

**Authors:** Nastaran Salehi Marzijarani, Roozbeh Yousefi, Arvind Jaganathan, Kumar Dilip Ashtekar, James E. Jackson, Babak Borhan

**Affiliations:** a Department of Chemistry , Michigan State University , East Lansing , Michigan 48824 , USA . Email: babak@chemistry.msu.edu ; Email: jackson@chemistry.msu.edu; b Dow AgroSciences LLC , 9330 Zionsville Road , Indianapolis , IN 46268 , USA

## Abstract

Though (DHQD)_2_PHAL-catalyzed chlorocyclizations of 1,1-disubstituted olefins show useful (and in some cases, reversible) asymmetric induction, stereochemically complete descriptions of these alkene additions have remained largely unknown. Herein, based on a combination of NMR, derivative, isotope labeling, and computational studies, we present detailed stereochemical analyses of chlorocyclizations of nucleophile-tethered 1,1-disubstituted styryl systems. The selectivities of the two asymmetric bond-forming processes, namely electrophilic chlorine attack and nucleophilic ring closure, are thus mapped out independently. Under the established optimal conditions, four related chlorocyclizations were subjected to this analysis. All showed a strong preference for Cl^+^ delivery from the same face of the alkene. However, depending on reaction conditions and substrate identity (carboxylic acid, amide or carbamate), the internal nucleophiles may close with a strong net preference for either *syn* or *anti* addition relative to the Cl atom. Studies of both uncatalyzed and (DHQD)_2_PHAL-catalyzed processes place new boundary conditions on the role of the catalyst in these reactions.

## Abstract 2

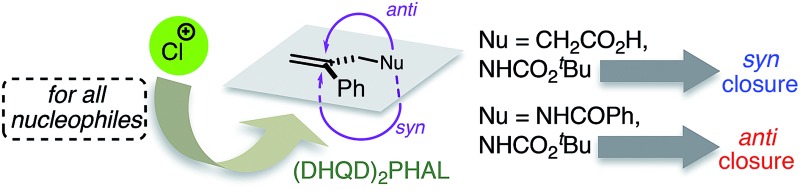
For four related 1,1-disubstituted olefins, (DHQD)_2_PHAL-catalyzed asymmetric chlorocyclization delivers Cl^+^ uniformly to one π face, but cyclizes with strong but differing net *syn vs. anti* addition.

## Introduction

With the advent of effective asymmetric catalytic methods, halocyclizations (and more broadly, electrophilic haloadditions to alkenes) have now emerged as useful tools for synthesis of chiral targets.^1^ Like the Nobel prize-winning asymmetric epoxidation and dihydroxylation reactions, halocyclization to alkenes had been known for many decades. But despite its obvious desirability and earlier valiant attempts, asymmetric control with significant enantiopreferences remained elusive. Reported in 2010,^2^ efforts from our group (and now many others) then discovered effective reagents, catalysts, and conditions to achieve chlorocyclizations with useful levels of stereocontrol, opening the floodgates of empirical exploration and synthetically valuable discovery, but leaving mechanistic understanding behind.

Broadly, stereocontrol requires the reaction environment to be desymmetrized with a chiral catalyst that activates the reaction while guiding bond formation to prefer one face of the alkene. Many asymmetric halocyclizations of alkenes have now been described using a variety of chiral catalysts. Chiral phosphoric acid catalysts or related analogs were shown to catalyze bromo-amination and -etherification by Shi and coworkers;^3^ bromoetherification by Denmark and Burk;^4^ fluoro/bromo/iodo cyclization of amides by Toste and coworkers;^5^ bromo/iodo etherification by Hennecke and coworkers;1f,^6^ and iodolactonization by the Ishihara group.^7^ Fujioka and coworkers disclosed an asymmetric bromolactonization of olefins catalyzed by chiral benzene trimers.^8^ Yeung and coworkers showed that bromo-amination and etherification can be catalyzed by C_2_ symmetric seleno-THF analogs with high enantioselectivity,^9^ whereas asymmetric bromolactonization is achieved by chiral pyrrolidines possessing a thiocarbamate functionality.^10^ More recently the Johnston and Hansen groups reported asymmetric iodolactonizations mediated by chiral PBAM and squaramide based catalysts, respectively.^11^ An elegant report by Jacobsen and coworkers showed iodocyclizations mediated by urea/thiourea catalysts with high enantioinduction.^12^ New binaphthyl analogs reported by Martin and coworkers proved efficient as catalysts for asymmetric bromolactonization.^13^ Most directly relevant to the present report, several research groups including those of Sun, Yeung, Mukherjee, and Tang demonstrated highly enantioselective intramolecular halofunctionalization reactions catalyzed by monomeric cinchona alkaloid derivatives.2b,^14^ A number of excellent reviews can be consulted for a complete coverage of the literature in this area.^1^

Our own and others' studies have uncovered several stereoselective halofunctionalizations based on the organocatalyst (DHQD)_2_PHAL.2a,14b,^15^ This C_2_ symmetric cinchona alkaloid derivative efficiently catalyzes the reaction of substrates bearing hydrogen-bond donor groups such as unsaturated amides, carbamates, naphthols and carboxylic acids. In all these asymmetric halocyclization reactions, the olefin undergoes electrophilic attack by a halenium ion (X^+^) donor with intramolecular ring closure by a pendant nucleophile. Mechanistically, halofunctionalization of alkenes has been extensively studied since their discovery. The exclusive formation of *anti*-products from olefin halogenation led Kimball in 1937 to propose a stepwise mechanism, with symmetrically bridged haliranium ions as putative intermediates.^16^ However, the groups of Fahey, Sauers and others provided firm evidence for open β-halocarbenium ion intermediates in halofunctionalizations of unsymmetrical alkenes, especially those with aryl or other π-delocalizing substituents.^17^ Furthermore, seminal works by Fahey, Poutsma, Williams, and others concluded that neither the bridged nor the open cationic intermediates are completely compatible with the observed experimental outcomes.^18^ Specifically, reaction rates were accelerated by proximity of the intramolecular nucleophile to the alkene, pointing clearly to a concerted Ad_E_3-type mechanism.17a,^19^ Our own recent work in this area has reaffirmed and further illustrated the critical role of the nucleophilic addition partner in activating olefins to abstract the electrophilic halenium ion from its donor reagent. Here, the intrinsic halenium ion affinity^20^ of the alkene pi system is boosted by contact with the nucleophile, favoring Ad_E_3-type concerted halofunctionalization. This nucleophile assisted alkene activation (NAAA)^21^ pathway represents a less familiar but often dominant alternative to the usual textbook scheme of stepwise halofunctionalization wherein initial formation of a bridged halonium ion is followed by its *anti* opening with a nucleophile.

**Fig. 1:**
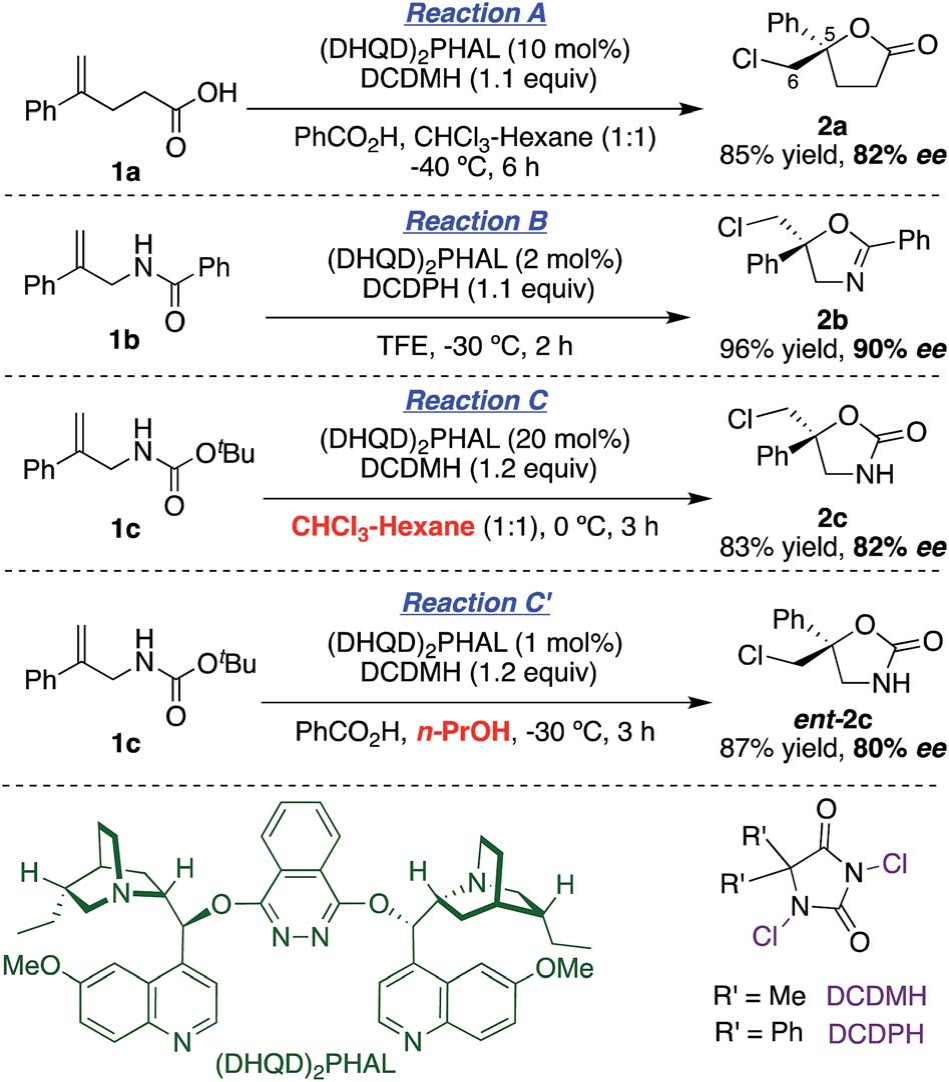
Asymmetric chlorocyclization of alkenoic acid **1a** (Reaction A), unsaturated amide **1b** (Reaction B), and carbamate **1c** under two different conditions (Reactions C and C′).

With asymmetric halocyclizations empirically established as useful synthesis tools, we turned to mechanistic studies of reactions developed in our labs over the past six years. Fig. 1 illustrates the four chlorocyclizations chosen for study.2a,15b,15d In the unlabeled systems as originally reported, the newly formed sp^3^ CH_2_Cl center lacked observable stereochemistry, leaving the face selectivity of chlorination unknown. To reveal the relative and absolute stereochemical outcomes of both the halenium ion attack and the nucleophilic closure in halocyclizations, we resorted to deuterium-labeling in the 1,1-disubstituted olefin substrates. In particular, we investigated the *syn* : *anti* selectivity of addition across the double bond, both for non-catalyzed reactions (to evaluate their intrinsic reactivity) and for their (DHQD)_2_PHAL-catalyzed analogues.

## Results and discussion

This study sought to uncover and understand the stereochemical relationships between the chlorenium ion delivery and the nucleophilic ring closure events that form the adducts shown in Fig. 1. Some key similarities and differences among the reactions of carboxylic acid **1a**, and those of amide **1b** and carbamate **1c**, should be noted at the outset. As earlier reported, (a) all these reactions employ (DHQD)_2_PHAL as the chiral catalyst and chlorinated hydantoins as the electrophilic chlorinating agents. (b) The configuration of the newly created stereogenic carbon (C5) in the product is nucleophile dependent. (c) In the case of the carbamate substrate **1c**, the solvent can also modulate the C5 configuration.

To understand these diverse behaviors, we asked the following questions: (a) is there a facial preference for electrophilic chlorine attack on the 1,1-disubstituted olefin? (b) If so, how strong is the preference, and how does it vary among substrates and conditions? (c) What patterns (if any) of stereochemical relationships are found between the chlorine atom and the nucleophile in the final adduct? Net *syn* or *anti* halocyclization would shed light on the nature of the reaction path. (d) Is the overall reaction concerted or stepwise; and if the latter, what sort of intermediate (*e.g.* bridged chloriranium ion, open benzylic carbocation, other?) might be formed? (e) How is the ultimate enantioselectivity set?

As in our previous mechanistic investigations of Reaction A,^22^ the isotopic labeling enables full characterization of the addition stereochemistry in the chlorocyclization of unsaturated amides (Reaction B) and carbamates (Reactions C and C′, Fig. 1). The presence of the deuterium in **1a-D**, **1b-D**, and **1c-D** leads to diastereomeric products that reveal not only the face selectivity of chlorenium ion attack on the olefin, but also the *syn* or *anti* relationship of addition between the delivered halogen and the captured nucleophile. The results of these studies show a diversity of relative and absolute stereochemical fates in both chlorenium ion delivery and ring closing processes. They also highlight the multiple ways that the catalyst can modulate reactivity.

### Synthesis of labeled substrates

**Scheme 1:**
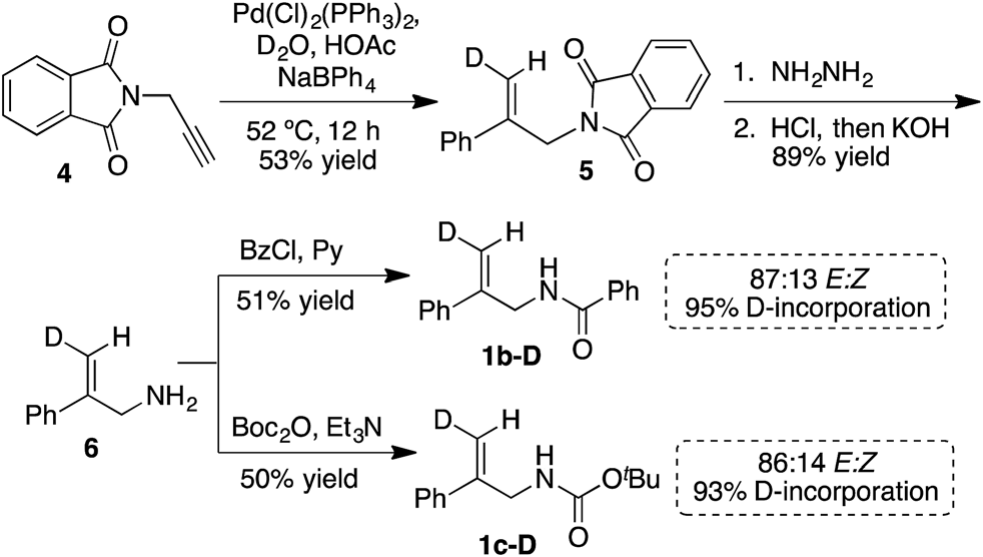
Synthesis of **1b-D** and **1c-D**.

The synthesis of the *E*-deuterated substrates **1b-D** and **1c-D** was accomplished in four steps (Scheme 1). The deuterium was incorporated through palladium-catalyzed *syn* hydrophenylation of **4** with sodium tetraphenylborate in D_2_O/acetic acid to afford **5**.^23^ Hydrolysis of imide **5** and subsequent derivatization of the deuterated alkenoic amine **6** with the appropriate acylating agent led to the formation of the desired substrates **1b-D** and **1c-D** with a high level of deuterium incorporation and good *E*/*Z* selectivity. For the final analysis of the chlorocyclization product ratios, the stereochemical impurity (∼94%) of the deuterated substrates **1b-D** and **1c-D** was taken into account by the mathematical treatment shown in the ESI.†

### Absolute stereochemical determination

**Scheme 2:**
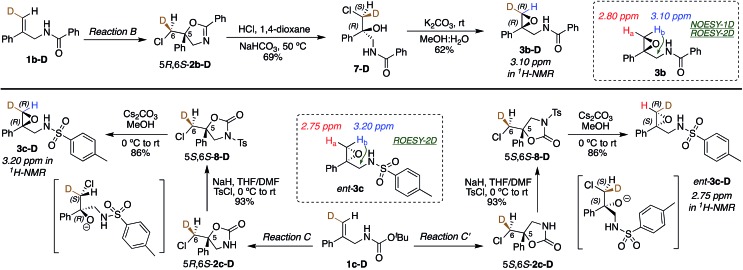
Absolute stereochemical assignment at the deuterated center (C6) for substrates **2b-D** (top) and **2c-D** and *ent*-**2c-D** (bottom).

(DHQD)_2_PHAL catalyzed chlorocyclizations of **1b-D** and **1c-D** (*vide infra*) led to mixtures of diastereomeric products. The absolute configuration of the deuterated chlorocyclized products **2b-D** and **2c-D** at C6 was determined *via* transformation of the major product of both deuterated and non-deuterated substrates to an epoxide with known configuration (Scheme 2, top). Hydrolysis of oxazoline 5*R*-**2b-D** (absolute configuration at C5 was determined previously by X-ray crystallography)15*d* with HCl afforded the *N*-benzoyl β-amino alcohol **7-D** (Scheme 2, top). The resulting halohydrin intermediate was treated with K_2_CO_3_ to afford the 1,1-disubstituted epoxide **3b-D** under mild conditions. Non-deuterated epoxy amide **3b** was synthesized similarly. ROESY and NOESY studies on the epoxy amide **3b** established the relative stereochemistry of *H*_a_ (2.80 ppm, *cis*) and *H*_b_ (3.10 ppm, *trans*) with respect to the phenyl group. ^1^H NMR analysis of the epoxy amide **3b-D**, obtained from the product of the chlorocyclization of **1b-D***via* Reaction B conditions, exhibits the peak at 3.10 ppm, establishing that the deuterium has a *cis* orientation with respect to the phenyl group. This leads to the assignment of *R* configuration for the carbon bearing the deuterium in epoxy amide **3b-D**. Since the epoxy amide is formed through the S_N_2 closure of the corresponding chlorohydrin intermediate, the *S* configuration at C6 is assigned for amide **2b-D** (major product of Reaction B, Scheme 2, top).

In an analogous study, the absolute configuration at C6 in products obtained from the chlorocyclization of the carbamate **1c-D** under conditions denoted as Reaction C and C′ were determined (Scheme 2, bottom). Tosyl protection of oxazolidinone 5*R*-**2c-D** and 5*S*-**2c-D** (configuration of C5 was established previously *via* X-ray crystallography),15*b* followed by CsCO_3_ mediated ring opening of the resulting chlorohydrin intermediate, gave 1,1-disubstituted epoxy sulfonamide **3c-D** and *ent*-**3c-D**. The non-deuterated epoxy amide *ent*-**3c** was synthesized from 5*S*-**2c** following the same reaction protocols. ROESY analysis of epoxy amide *ent*-**3c** indicated that *H*_a_ (2.75 ppm) is *cis* to the phenyl group, while *H*_b_ (3.20 ppm) is *trans*. ^1^H NMR data obtained for the epoxides derived from the major products of Reaction C and C′ chlorocyclization reveal that the absolute configuration on the labeled C6 center of the cyclized products 5*R*-**2c-D** and 5*S*-**2c-D** is *S* for both (Scheme 2, bottom).

### Stereochemical outcomes of uncatalyzed reactions

**Fig. 2:**
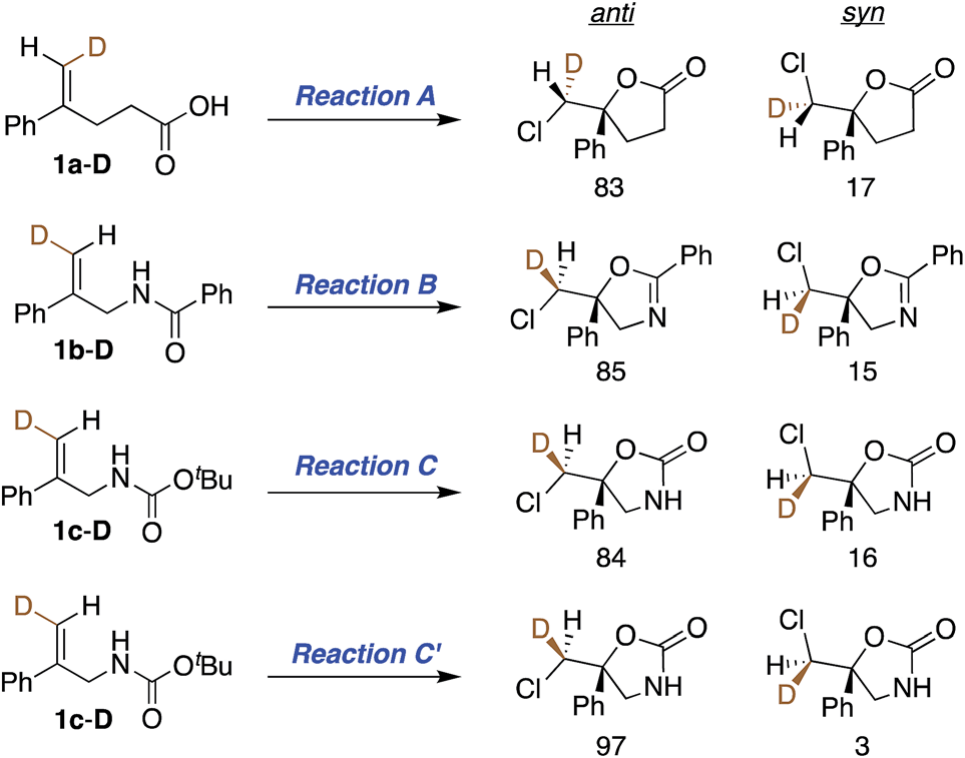
Summary of intrinsic *anti* : *syn* product ratios for uncatalyzed Reactions A, B, C, and C′ (enantiomeric pairs are not shown for clarity). Reaction conditions mimic those shown in Fig. 1, except that the catalyst was omitted, and the reactions were run at room temperature.

To understand the role of the catalyst in the enantiocontrolled reactions, the intrinsic diastereoselectivities of the uncatalyzed analogues of Reactions A–C and C′ were first investigated (Fig. 2).^24^ All favor *anti* addition; in Reaction A, chlorolactonization occurs with an *anti* : *syn* product ratio of 83 : 17.^22^ In Reaction B, amidoalkene **1b-D** reacts with 1,3-dichloro-5,5-diphenylhydantoin (DCDPH) sluggishly in TFE at room temperature to afford an 85 : 15 mixture of the two diastereomers. Similarly, predominant *anti* addition is found for the uncatalyzed reaction of carbamate **1c-D** with 1,3-dichloro-5,5-dimethylhydantoin (DCDMH), both in CHCl_3_-hexanes and *n*-PrOH solvent systems; there, *anti* : *syn* ratios were 84 : 16 and 97 : 3, respectively. Formation of significant quantities of the *syn* isomer serves as evidence that these reactions do not simply proceed *via* one path through a stereochemically-defined intermediate (*e.g.* a cyclic chloriranium ion) able then to dictate stereospecific cyclization to the *anti* isomer; at least two pathways must contribute to yield the two distinct diastereomers. The substantial shift in *anti* : *syn* selectivity (84 : 16 to 97 : 3) between the solvent conditions corresponds to an increase of ∼1 kcal mol^–1^ in the relative barrier for *syn vs. anti* closure; evidently carbamate **1c-D** interacts fairly strongly with its surroundings, as expected for such a polar substrate.

To probe the factors that determine *anti* : *syn* diastereoselectivity in the absence of catalyst, reaction parameters were systematically studied for chlorocyclization of the amidoalkene **1b-D**. First, the effects of the chlorenium ion source were investigated (Table 1, entries 1–8); this factor significantly affects the stereochemical outcome; all yielded mainly *anti* product, but *anti* : *syn* ratios varied from 65 : 35 to 85 : 15. Though the most reactive chlorinating agent (TCCA) does show the lowest selectivity, no direct correlation is seen between chlorenium ion donor ability (assessed *via* HalA values)^20^ and *anti* : *syn* ratios. Thus it appears that the departing moiety of the chlorine donor reagent remains involved at the point of diastereoselection.^25^ What appears unlikely is simple Cl^+^ delivery followed by ring closure in an intermediate free of the leaving group from the chlorinating agent.

**Table 1:** Screen of electrophilic chlorinating reagent, solvent and concentration effects on the *anti* : *syn* ratios of **2b-D**

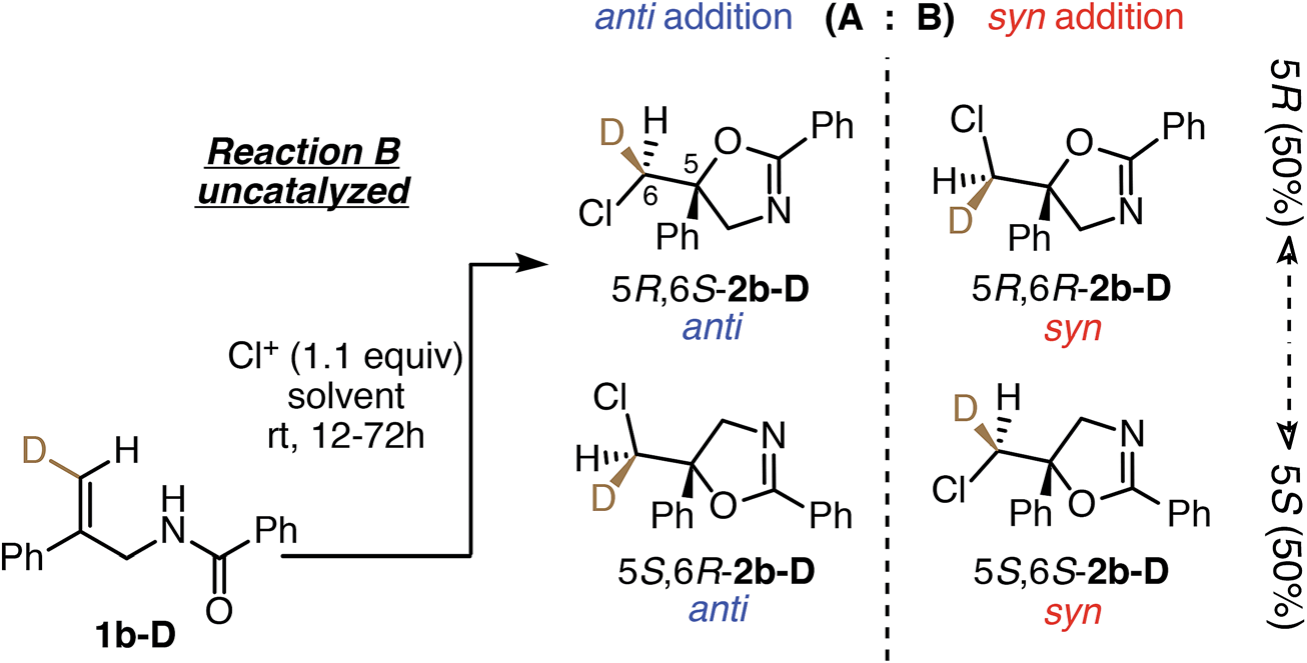						
Entry	Cl^+^ source	HalA or Solvent *ε*	Solvent	[Sub] M	[Cl^+^] M	dr (A : B) (*anti* : *syn*)
1	DiCh.T	273.3	TFE	0.05	0.05	90 : 10
2	NCSac	265.0	TFE	0.05	0.05	86 : 14
3	DCDPH	270.1	TFE	0.05	0.05	85 : 15
4	NCS	290.1	TFE	0.05	0.05	80 : 20
5^*a*^	Ch.T	268.2	TFE	0.05	0.05	80 : 20
6	DCDMH	275.7	TFE	0.05	0.05	79 : 21
7^*b*^	NCP	286.7	TFE	0.05	0.05	78 : 22
8	TCCA	252.1	TFE	0.05	0.05	65 : 35

9	DCDPH	2.38	Toluene	0.05	0.05	98 : 2
10^*c*^	DCDPH	3.35	CHCl_3_ : Hex	0.05	0.05	97 : 3
11	DCDPH	8.93	DCM	0.05	0.05	97 : 3
12	DCDPH	4.81	CHCl_3_	0.05	0.05	85 : 15
13	DCDPH	26.1	TFE	0.05	0.05	85 : 15
14	DCDPH	37.5	CH_3_CN	0.05	0.05	86 : 14

15	DCDPH	—	TFE	0.50	0.50	88 : 12
16	DCDPH	—	TFE	0.20	0.20	88 : 12
17	DCDPH	—	TFE	0.10	0.10	88 : 12
(13)	DCDPH	—	TFE	0.05	0.05	85 : 15
18	DCDPH	—	TFE	0.01	0.01	69 : 31
19	DCDPH	—	TFE	0.005	0.005	62 : 38
20	DCDPH	—	TFE	0.0025	0.0025	56 : 44

21	DCDPH	—	TFE	0.10	0.11	84 : 16
22	DCDPH	—	TFE	0.05	0.11	82 : 18
23	DCDPH	—	TFE	0.01	0.11	66 : 34
24	DCDPH	—	TFE	0.005	0.11	61 : 39

25	DCDPH	—	TFE	0.01	0.055	62 : 38
26	DCDPH	—	TFE	0.01	0.022	61 : 39
27	DCDPH	—	TFE	0.01	0.011	69 : 31

^*a*^58% conversion after 3 days.

^*b*^82% conversion after 3 days.

^*c*^(1 : 1) ratio of the solvent; shown dielectric constant is the average of the *ε* of the two solvents.

The effect of the reaction solvent on *anti* : *syn* ratios was next studied (Table 1, entries 9–14). The dr values showed significant solvent dependence; broadly, reaction in solvents of low polarity such as PhCH_3_ and DCM gave high *anti* : *syn* ratios (98 : 2 and 97 : 3) whereas relatively polar, hydrogen bonding solvents eroded the *anti* selectivities. Likewise, running the reaction in a 1 : 1 mixture of CHCl_3_-hexanes gave a much higher 97 : 3 dr than in CHCl_3_ alone (86 : 14). These results suggest contributions from mechanisms that are differentially affected by solvent polarity and hydrogen bonding ability. For instance, nonpolar solvents would favor substrate self-association *via* hydrogen bonds between the amide moieties,^26^ enabling a structurally defined, concerted path to form *anti* products from reagent + substrate dimers (see Fig. 4a). Polar, hydrogen bonding solvents would disrupt such associations, promoting *syn* product formation *via* the more statistically probable 1 : 1 reaction complex (Fig. 4b). We note here that CHCl_3_ should disrupt substrate aggregation more than CH_2_Cl_2_; CHCl_3_ has long been recognized as the stronger hydrogen bond donor solvent, despite its lower polarity as measured *via* dielectric constants.^27^ Notably, no intermolecular adducts were seen even in neat solvents capable of serving as nucleophiles (CH_3_CN and TFE), or when the amide substrate itself was present in concentrations as high as 0.5 M. Thus, any electrophilic intermediates, if formed, must have lifetimes shorter than trapping times in these neat nucleophilic solvents. And even the most aggressive chlorenium ion donors, expected to require little or no nucleophilic assistance, did not trigger intermolecular product formation.

**Fig. 3:**
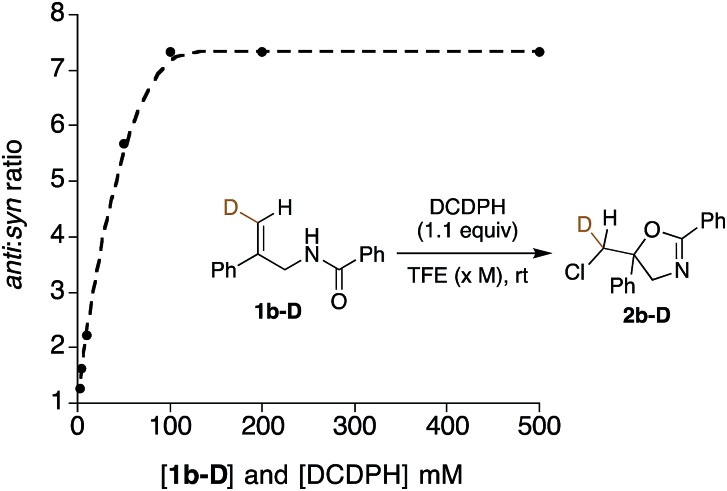
The plot of *anti* : *syn* ratios *vs.* concentration of DCDPH reagent and **1b-D** substrate reaction mixtures.

**Fig. 4:**
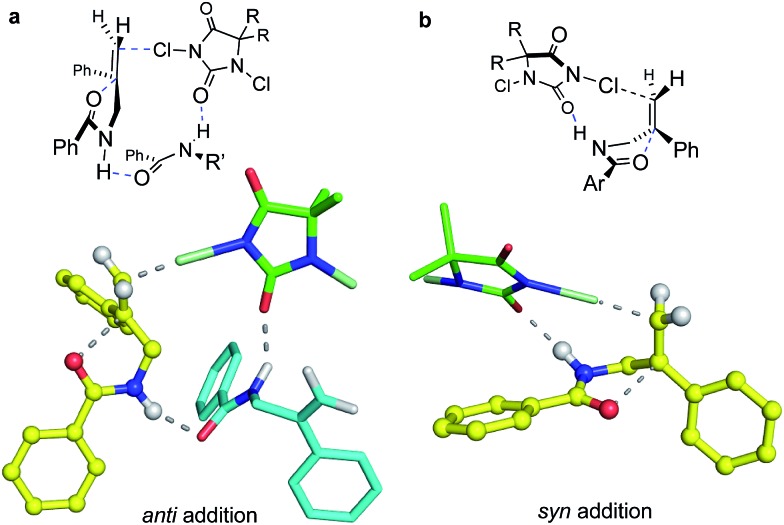
DFT-computed models for *anti* (a) and *syn* (b) cyclo addition of **1b**. For computational efficiency, the dimethyl hydantoin was modeled in place of the diphenyl reagent used in the experiments. The higher propensity for *anti* addition at higher concentrations is consistent with the *ca.* 15 kcal mol^–1^ lower activation free energy calculated for the termolecular addition TS (structure **a**) in which a second amide substrate molecule bridges from the chlorine-donating hydantoin to the N–H site of the cyclizing amide moiety. At lower substrate concentration, the more strained bimolecular path (structure **b**) leading to *syn* addition is favored.

Further mechanistic clues emerged from studies of the effects of reactant concentration on diastereoselectivity. Decreasing the concentration of both the substrate and DCDPH in TFE led to an increase in *syn* product formation (Table 1, entries 15–20). This is graphically illustrated in Fig. 3, where a steep dropoff in *anti* : *syn* ratio occurs at ∼0.05 M. Further explorations varying the individual component concentrations showed little change with varying [DCDPH] but significant falloff in *anti* selectivity as [**1b-D**] was lowered. These findings support the above suggestion that *syn* and *anti* products may arise *via* mechanisms of different reactant molecularities. More succinctly stated, at high concentrations, an *anti*-forming transition state could be composed of two (or more) molecules of olefin substrate and one molecule of the DCDPH reactant (Fig. 4a). An obvious mode of interaction involving two substrate molecules would be amide dimerization *via* NH–O hydrogen bonding, altering conformational preferences and enhancing both the effective amide group size and the nucleophilicity of the carbonyl oxygen. Such a complex would be expected to prefer *anti* addition as depicted in Fig. 4a. Here, the non-reacting amide serves as both the hydrogen bond relay and activator between the DCDPH and the N–H site of the reacting amide, enabling chlorenium ion transfer, ring closure, and proton transfer to take place concertedly with minimal charge separation.

In the 1 : 1 DCDPH : substrate complex that would be entropically favored at lower concentrations, the DCDPH may function as both the base and the chlorenium ion source (Fig. 4b), delivering the halogen from the same face as the nucleophile. The amide NH pyramidalization, and the twisting of the alkene seen in the calculated *syn* transition structure suggests that this species may suffer from substantial strain, consistent with its higher calculated barrier to reaction. On the other hand, the geometries of the hydrogen bonding interactions in the *anti* TS also appear non-ideal. Nonetheless, these modeled reaction paths are qualitatively consistent with the drop in *anti* : *syn* selectivity observed when the concentration of substrate in TFE was decreased from 0.10 M to 0.005 M with [DCDPH] held constant at 0.11 M (Table 1, entries 21–24). Conversely, when the concentration of substrate was kept constant at 0.01 M in TFE and concentration of DCDPH was lowered from 0.11 M (10.0 equiv.) to 0.011 M (1.1 equiv.), no significant changes were observed in diastereoselectivity (Table 1, entries 25–28). These findings point to a scenario with more than one substrate molecule but only one chlorinating agent in the transition state for *anti* cyclization (Fig. 4a).

Quantum chemical modeling of the above bi- and tri-molecular complexes at the T1//EDF2/6-31G* level, with TFE “solvation” simulated with the SMD solvent model,^28^ did indeed find a lower energy path for the termolecular than for the bimolecular process, as seen in Fig. 4; the energetics of these species are summarized in the ESI.† The free energy barriers from separated starting materials *via* these respective TS structures place the termolecular complex *ca.* 15 kcal mol^–1^ lower in energy than the bimolecular case. Solvation in TFE and in chloroform, as simulated at the SMD/EDF2/6-31G* level, modulates both paths but does little to change their relative energies. Aggregation *via* hydrogen bonded interactions, as seen in the termolecular complex, might seem unexpected in a hydroxylic medium such as TFE; however, this solvent is well known to promote polypeptide folding.^29^ On the other hand, entropy would strongly disfavor this higher molecularity structure at the dilute concentrations where *syn* products emerge.

**Fig. 5:**
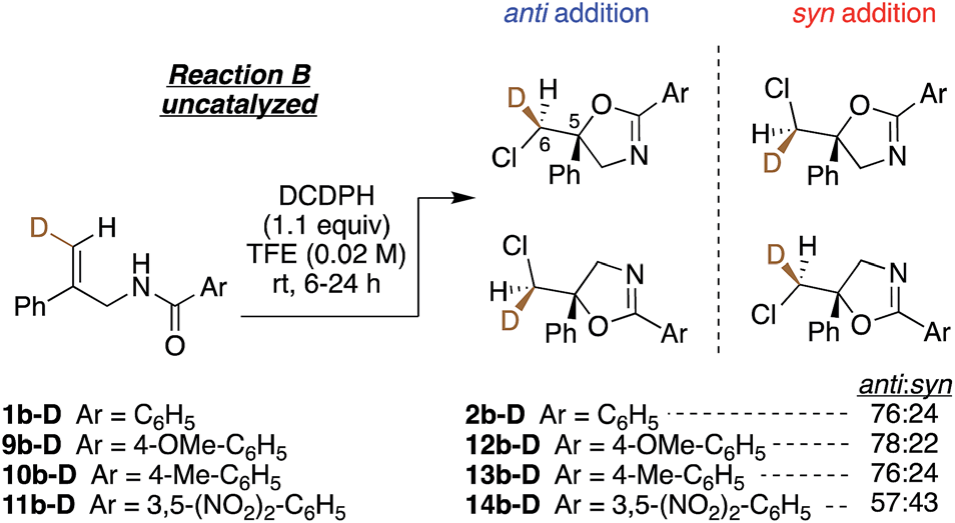
*Anti* : *syn* product ratios of electronically perturbed aryl amides.

Lastly, we investigated the effect of altering the electronic properties of the amide aroyl group on the diastereoselectivity of the chloro amidoalkene cyclization (Fig. 5). The largest change is seen with the 3,5-dinitro substituted benzamide **11b-D**, which yields a near 1 : 1 *anti* : *syn* ratio of products **14b-D**. Here, we speculate that reduced carbonyl group nucleophilicity in this substrate weakens dimer formation *via* hydrogen bonding, enhancing the contribution from the *syn*-favoring 1 : 1 reaction. On the other hand, the increased N–H acidity could favor interaction with the DCDMH, activating less selective chlorenium ion delivery reaction.

### Diastereoselectivity in catalytic asymmetric reactions

**Fig. 6:**
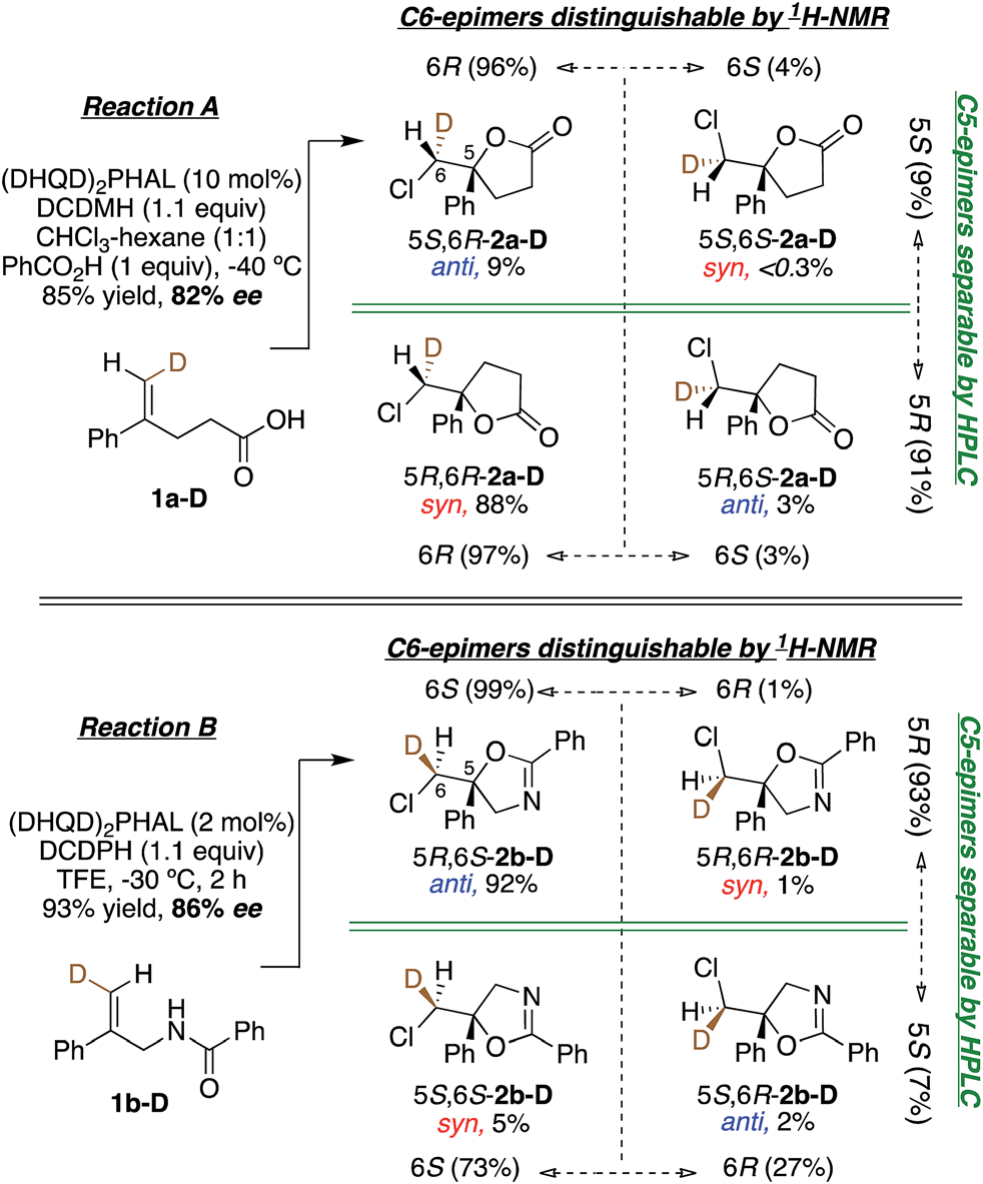
(DHQD)_2_PHAL catalyzed chlorocyclization of **1-D** leads to four isomeric products. Ratios of each isomer are quantified by ^1^H NMR and HPLC analysis. Reaction A (top, previously reported) shows *syn* selectivity in net addition of the chlorenium cation and carboxylate anion to olefin **1a-D**.^22^ Noting that deuterium differs in configuration between **1a-D** and **1b-D**, and in CIP group priorities about C5 in the product, Reaction B, (bottom) shows predominant *anti* addition with C6-pro-*S*/C5-pro-*R* selectivity in this catalyst templated addition of the chlorine electrophile and the amide nucleophile across the olefin.

Having probed the intrinsic diastereoselectivity of uncatalyzed Reaction B, we turned to the stereochemical analysis of the (DHQD)_2_PHAL catalyzed asymmetric reactions. As reported earlier, chlorolactonization of alkenoic acid **1a-D** (Fig. 1, Reaction A) effects *syn*-selective carboxylate/chlorenium ion addition with an overall 88 : 12 *syn* : *anti* preference (Fig. 6, top).^22^,^30^ In Reaction B, the (DHQD)_2_PHAL-catalyzed chlorocyclization of deuterated amide **1b-D** yields four stereoisomers under the previously reported catalytic asymmetric conditions (Fig. 6, bottom).15*d* The facial selectivity of chlorination, as determined *via*^1^H NMR analysis of the HPLC purified diastereomers (see ESI† for full details), reveals that the *anti* product is the major component of the mixture (94%). Thus, (DHQD)_2_PHAL controls the facial selectivity of chlorenium ion attack, forming the major epimer with a 99 : 1 preference for the 6*S* configuration, but only moderate 6*S* selectivity in forming the minor (5*S*) product (73 : 27). The nucleophilic closure occurs with high selectivity (93 : 7 ratio) favoring the *R* configuration at C5. As such, the two bond-forming events appear to be independently controlled by the catalyst. Noteworthy is the fact that in Reactions A and B (chlorolactonization and chloroamidocyclization, respectively), the chlorenium ion delivery occurs to the same face of the olefin, but ring closure occurs on opposite faces of the cyclizing carbon (note that differing substituent priorities designate both products 5*R*, while differing C6 configurations in **1a-D***vs.***1b-D** and **1c-D** lead to opposite C6 configurations in the respective products **2** for the same facial preference of Cl attack). The overall process, therefore, is *syn* for chlorolactonization (Reaction A) and *anti* for chloroamidocyclization (Reaction B).

**Fig. 7:**
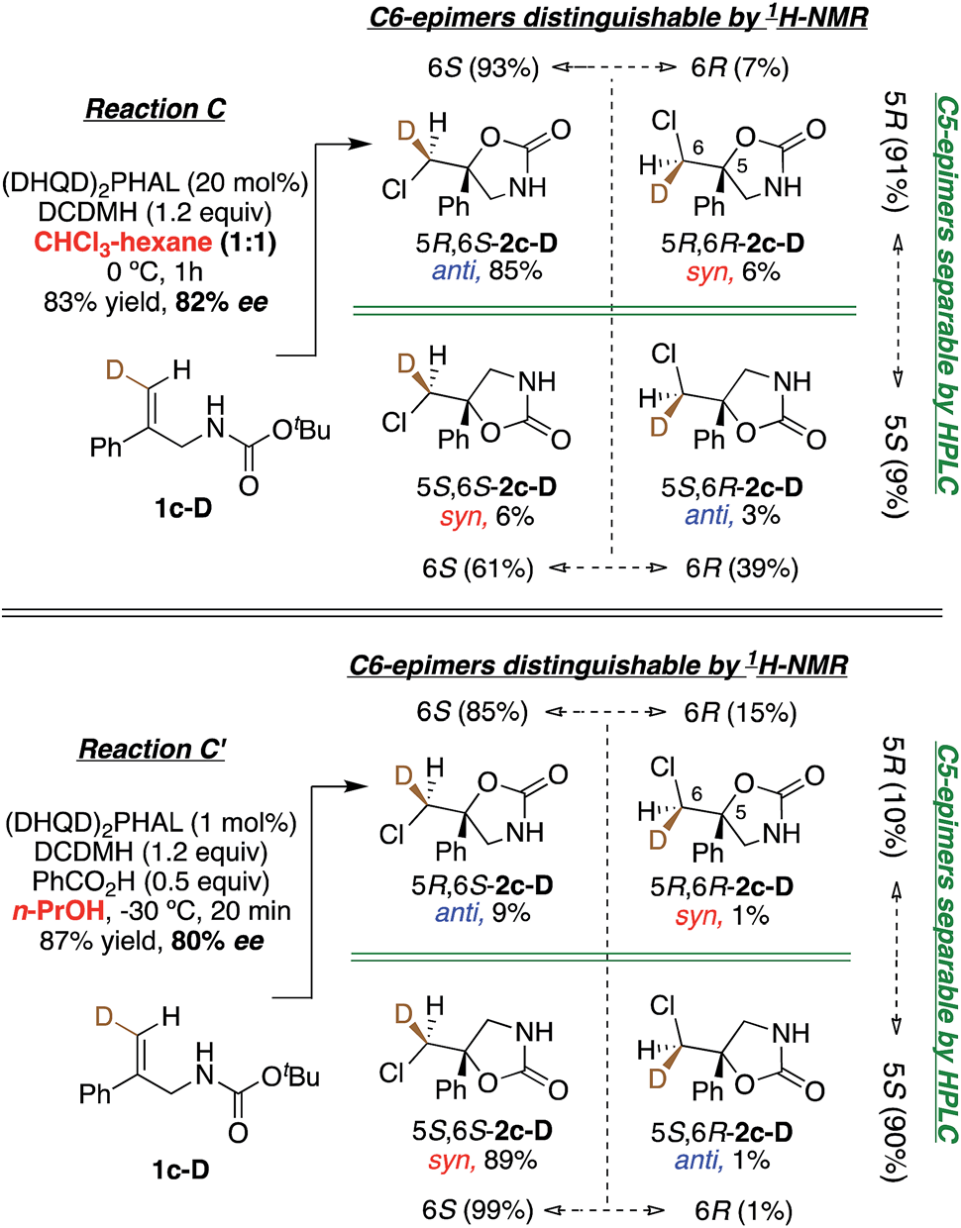
Chlorocyclization of carbamate **1c-D** yields the *anti* product as the major isomer in 1 : 1 chloroform : hexanes (Reaction C, top), and the *syn* product as the major isomer in *n*-PrOH (Reaction C′, bottom). Both reactions are catalyzed by (DHQD)_2_PHAL.

In Reactions C and C′, carbamate **1c** shows a switch in the enantiopreference of (DHQD)_2_PHAL-catalyzed chlorocyclization depending on reaction conditions – primarily the reaction solvent.15*b* The overall stereochemistry of both these asymmetric alkene additions is now revealed *via* deuterated probe **1c-D**. Chlorocyclization of **1c-D** catalyzed with (DHQD)_2_PHAL in 1 : 1 CHCl_3_ : hexane (Reaction C, Fig. 7, top) yields the *anti* product 5*R*,6*S*-**2c-D** as the major isomer (85%); this behavior is thus stereochemically the same as for amide **1b-D**. The chlorenium ion delivery to yield products with 6*S* configuration occurs with high selectivity (93 : 7) for the major (5*R*) diastereomers. As in the above cases, although the 6*S* selectivity is still predominant for the minor diastereomer, it occurs with reduced discrimination (61 : 39). Thus, the overall face selectivity for chlorination on the pro-*S* face of the =CHD site (*i.e.* 6*S* : 6*R*) is 91 : 9. This C6 stereoselectivity incidentally has the same numerical value as the enantioselectivity of the reaction (91 : 9 5*R* : 5*S*).

When applied to Reaction C′ (Fig. 7, bottom), the above analysis reveals a contrast relative to both the non-catalyzed reaction in *n*-PrOH and the catalyzed Reaction C in CHCl_3_-hexanes where *anti* additions predominate (97 : 3 and 88 : 12, respectively). Instead, the (DHQD)_2_PHAL catalyzed reaction in *n*-PrOH effects *syn* addition (net *anti* : *syn* = 10 : 90). However, as in all the other reactions discussed so far, chlorenium ion delivery occurs to the same face, forming the 6*S* epimers (with this substrate) in high (99 : 1) and good (85 : 15) enantioselectivity for the major and minor diastereomers, respectively. The net selectivity for chlorination of the C6 pro-*S* face of **1c-D** is 98 : 2 whereas the C5 face selectivity is somewhat lower, at 90 : 10 pro-*S*.

**Scheme 3:**
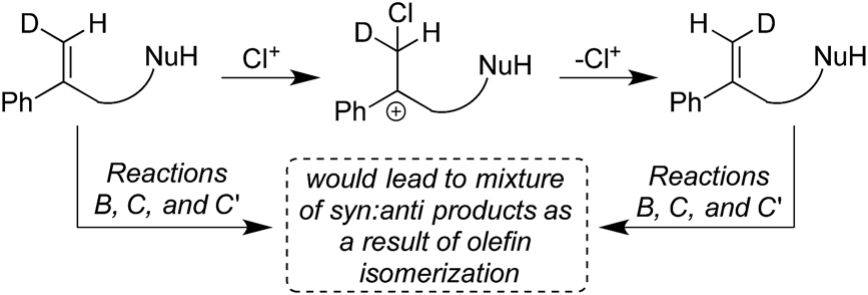
Possibility of the formation of the isomerized starting material, ruled out by recovery studies, see Table S3.†

Given the various *syn* : *anti* addition ratios seen as a function of starting material and reaction conditions, it might be suggested that *cis*–*trans* isomerization in the starting olefin could explain the observed stereo-randomized products (see Scheme 3 for this hypothetical pathway). This scenario was ruled out (a) implicitly, by the large C6 stereoselectivities seen in some cases (*e.g.* 99 : 1 in Reaction B), and (b) explicitly, by verification of the stereochemical integrity of labeled substrates **1b-D** and **1c-D** recovered during the course of Reactions B, C, and C′. Reactions quenched at various extents of conversion yield recovered alkenes with *E*/*Z* isomeric ratios identical within measurement uncertainties to the starting ratios for all three reactions (see Table S3†). Besides showing that isomerized reactant is not the source of the disfavored addition products, these results agree with earlier findings that chlorenium ion transfer is not reversible.^22^,^31^

**Fig. 8:**
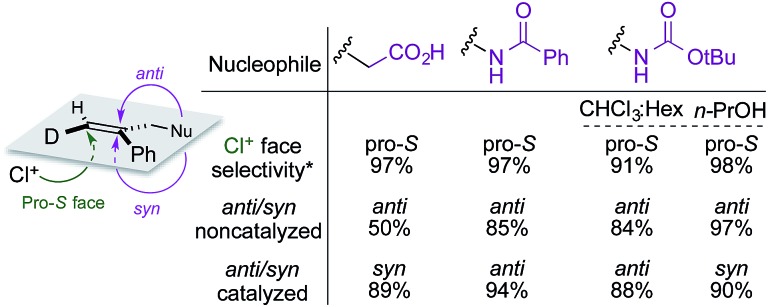
Summary of stereochemical outcomes for Reactions A, B, C, and C′. *Configuration designators are listed as shown in the image at left.

A summary of the stereochemical outcomes of the various asymmetric chlorocyclization reactions disclosed here is presented in Fig. 8. Most striking is the uniform and selective delivery of chlorine to the same face of C6 in all the alkene substrates. On the other hand, the net *syn* addition of halogen and nucleophile across the olefin that we had first observed in the initial chlorolactonization chemistry is by no means general. Despite the use of the same organocatalyst and similar hydantoin chlorine sources, different cyclization stereopreferences dominate with different substrates and reaction conditions. Related work from other laboratories5d,^7^,^8^,^10^–^14^,15a,^32^ seems likely to exhibit a similar diversity of *syn* and *anti* addition across 1,1-disubstituted alkenes.

Though a comprehensive quantum chemical simulation of interacting substrates, organocatalyst, reagents and solvents is beyond the scope of this report, limited simulation studies of the complexation and reaction processes of carbamate substrate **1c** were pursued using the Spartan'16 software.^33^ As noted earlier, the ring closure stereochemistry of **1c** switches from *syn* to *anti* when the medium is changed from *n*-PrOH to CHCl_3_/hexane. Based on preliminary NMR studies that suggest a 2 : 1 binding of substrates in the (DHQD)_2_PHAL organocatalyst, broad conformational searches on such 2 : 1 complexes were performed with the MMFF94 (ref. 34) and Sybyl^35^ force fields. These simulations typically explored 1–2000 conformations and were repeated multiple times from various arbitrary starting geometries. They consistently turned up low energy structures that exposed the pro-*R* alkene face, the experimentally preferred site of chlorine attack. Conformations in the lowest 10 kcal mol^–1^ range were then re-optimized using the PM6 semiempirical molecular orbital model.^36^ Again, the lowest energy conformations found were orientations with the pro-*R* face of the alkene exposed. A second series of conformational searches of similar breadth was generated by placing the PM6 calculated transition structures (TSs) for *anti* and *syn* chlorocyclizations into one (DHQD)_2_PHAL pocket, together with a partner non-reacting substrate complexed to the other face. In these cases, the lengths of the partial (reacting) bonds (N–Cl, Cl–C, and C–O), were held constant at the gas-phase TS values, but all other degrees of freedom were allowed to vary in the conformational search. Again, the lowest energy set of resulting conformations was reoptimized (still constrained) using PM6, after which full TS optimizations were completed. The resulting *syn* and *anti* TSs (each with a single imaginary vibrational frequency corresponding to the alkene addition trajectory) were reevaluated *via* single-point B3LYP-D3/6-31G* energy calculations,28a,^37^ with solvation corrections for chloroform and *n*-PrOH solvents (dielectric constants of 4.8 and 20.3) computed *via* the C-PCM method.^38^

**Fig. 9:**
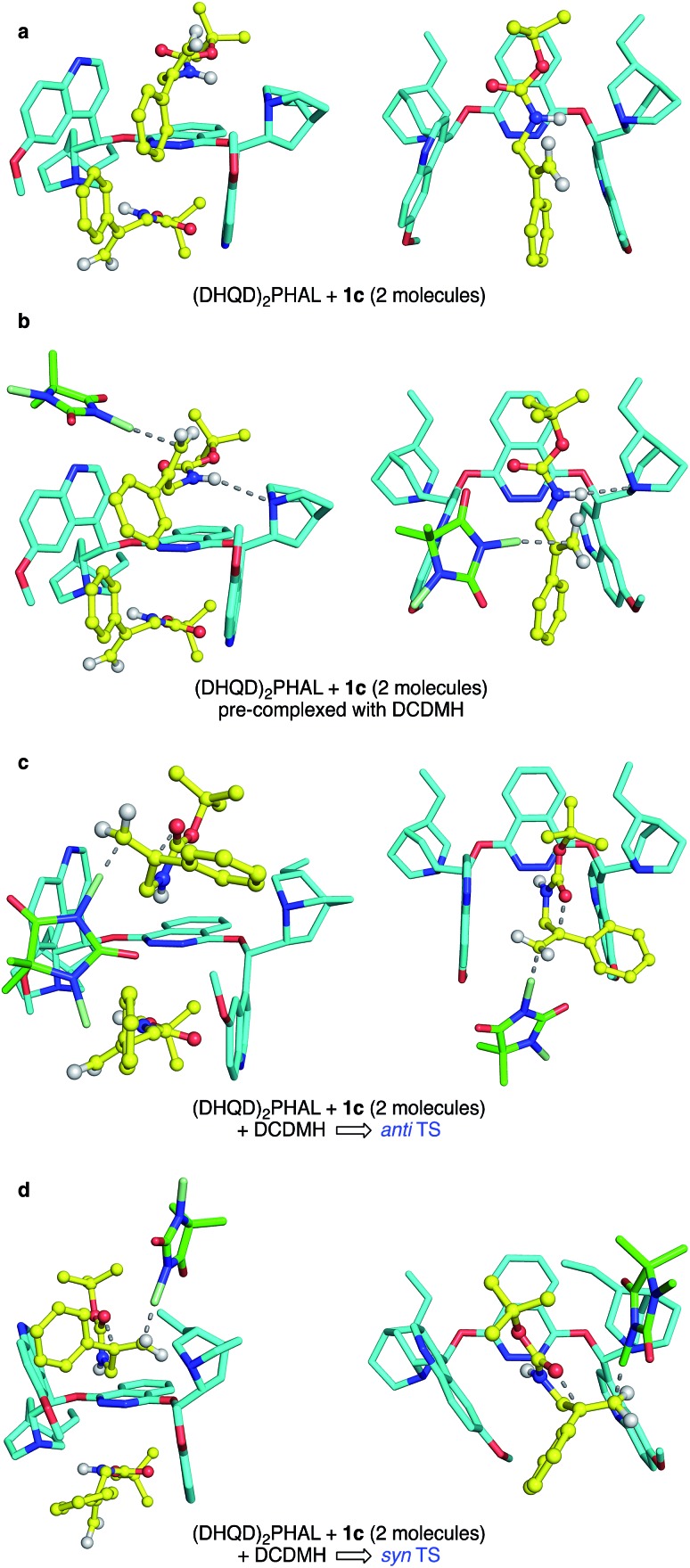
Side (left) and top (right) view of (DHQD)_2_PHAL complexed with **1c** and DCDMH. Hydrogen atoms are not shown in all structures except for the substrate amide and vinylidene protons. In the top view, the second bound substrate is omitted for sake of clarity. (a) (DHQD)_2_PHAL bound with **1c**. (b) Ground state, pre-complexed interaction of DCDMH with **1c**, bound to catalyst, showing the preferred olefin face selectivity as a result of the orientation of the substrate. (c) The *anti* TS, illustrating a concerted path to product formation. (d) The *syn* TS, also in a concerted path toward product.

The final complexes identified *via* the above procedures are displayed in Fig. 9. Given the relatively low level of theoretical models used to develop them, these structures must be understood mainly as proposed guides for visualization. Nonetheless, we were encouraged by the predominance of low energy catalyst–substrate complex conformations (Fig. 9a) that orient the alkene to expose the face that is actually chlorinated. Likewise, the close energies calculated for the *anti* and *syn* transition state structures correspond well with the fact that the difference between CHCl_3_ and *n*-PrOH media can switch the *anti*/*syn* addition selectivities of Reactions C/C′. Notably, the relaxed precursor complex (Fig. 9b) positions the carbonyl oxygen close to the alkene plane, suggesting that its rotations to approach to either side might face similar barriers. Whether closing in *anti* or *syn* modes (Fig. 9c and d, respectively), the extended carbamate backbone lying in the catalyst groove must fold up to a TS conformation that brings the carbonyl into contact with the alkene, activating the chlorine transfer and closing the ring. Both TS structures have thus lost factors that stabilized the bound GS, specifically van der Waals interactions between substrate *t*-butyl groups and the phthalazine ring, and among substrate phenyl groups and the quinoline side “walls” of the catalyst. The amide N–H site, however, remains associated with catalyst nitrogen atoms by hydrogen bonding, which also activates the nucleophilicity of the carbonyl oxygen as it closes to form the oxazolinone ring.

Energetically, the TS structures for *anti* and *syn* (Fig. 9c and d) are calculated to be almost 40 kcal mol^–1^ higher than the relaxed (DHQD)_2_PHAL-carbamate-DCDMH complex (Fig. 9b) at the B3LYP-D3/6-31G*//PM6 level. These high DFT-based barriers change surprisingly little upon extension of the calculations to include CPCM simulated *n*-PrOH and CHCl_3_ solvation environments. Interestingly, the pure PM6//PM6 barriers fall in the much more reasonable 20 kcal mol^–1^ range (see SI for summary) with a small (4.1 kcal mol^–1^) free energy preference for *syn*. We suggest that the more intimately bound complexes in Fig. 9a and b are over-stabilized by the well-known overestimation of van der Waals interactions in B3LYP and related DFT methods, augmented by the basis set superposition errors of the relatively small 6-31G* basis set. Unfortunately, the solvation calculations, which include no geometry relaxation or explicit solvent interactions, offer little insight into the solvent-switched selectivities of Reactions C/C′.

The work presented above highlights one of the fundamental challenges in developing highly enantioselective halocyclizations and more generally, additions across alkenes. Here, exceptional face-selectivity in the alkene chlorination is no guarantee of a strong enantiopreference for the newly created C5 sp^3^ stereocenter; *syn* and *anti* addition paths are in close competition. Conversely, a finding of poor enantioselectivity at C5 does not imply poor face-selectivity in the halogen-alkene bond formation.

With the present array of data, our working mechanistic interpretation is that reaction occurs *via* intramolecular versions of the Ad_E_3 olefin addition.^39^ Here, preorganization of the catalyst and substrate expose the preferred alkene face to the incoming chlorenium ion donor, while still allowing the nucleophilic moiety the conformational flexibility to fold in to contact either face of the alkene. The calculations suggest that the chlorinating agent may form a weak preassociation with the complexed alkene, but the actual bond-forming addition of the halogen to the alkene requires the nucleophile to fold to a conformation that contacts and activates the pi bond for the concerted addition. We recently explored such concerted paths in the substrate framework of Reaction A, where nucleophile-assisted alkene activation (NAAA) promoted non-catalyzed chlorolactonization of 1,1-disubstituted alkenoic acids. That both *syn* and *anti* paths can selectively occur among reactions A–C rules out bridged chloronium ions as stereocontrolling intermediates, as expected for these conjugated, 1,1-disubstituted alkenes.

What remains difficult to explain is the observed variability in the ring closure stereochemistry for the four highlighted reactions. As noted above, the complexed alkenes in our calculated structures (see Fig. 9b) place the carbonyl oxygen nearly in the plane of the alkene, establishing no obvious preference for which alkene face would be more easily accessed. Yet amide and carbamate reacting in a non-polar solvent system (Reactions B and C′) proceed with *anti* ring closure in contrast to the *syn* mode seen for carboxylic acids and for carbamates reacting in polar protic solvents. We propose that the amides and carbamates **1b** and **1c** (both more nucleophilic and, with only two freely rotating bonds, less conformationally flexible than **1a**) most readily fold “inward” to achieve the pro-*anti* conformation. In contrast, carboxylic acid **1a** is less conformationally restricted and potentially capable of hydrogen bond formation, which enables a preference for it to fold outward and activate the alkene from the same face approached by the chlorine-donating hydantoin. Likewise, folding of the polar carbamate moiety of **1c** out into the solvent could be supported by the more polar *n*-PrOH medium, favoring approach from the same face as the chlorenium ion delivery. The carbamate in *n*-PrOH (Reaction C′) thus behaves much like the carboxylic acid **1a**, yielding mainly *syn* addition product.

## Conclusions

For a family of chlorocyclizations, both uncatalyzed and mediated by (DHQD)_2_PHAL, the relative and absolute stereochemical outcomes have been fully analyzed. In four distinct (DHQD)_2_PHAL—catalyzed processes—chlorocyclizations of carboxylic acid **1a**, of amide **1b**, and of carbamate **1c**2a,15b,15d under two sets of conditions—the chlorine attacks the same face of the olefin. This high facial selectivity for chlorenium ion delivery presumably reflects catalyst-mediated pre-organization of the styrene substrate, directing chlorine donor access to only one alkene face. Cyclization by nucleophilic bond closure can show high *syn* or *anti* selectivity depending on the nature of the nucleophile and the medium. Thus, the net stereoselectivities at the two new stereocenters appear to be related only in the sense that one of these concerted paths is strongly preferred over others in the optimized reactions. The resulting structural insights place boundary conditions on any mechanistic hypothesis proposed to further refine and generalize this synthetically versatile class of transformations. Detailed kinetic analyses and simulation efforts are ongoing to probe molecularity, catalyst-substrate-reagent binding, preferred conformations in different settings, and reaction rate effects.

## Conflicts of interest

There are no conflicts to declare.

## Supplementary Material

Supplementary information
