# Supplementary material for: Absolute and relative facial selectivities in organocatalytic asymmetric chlorocyclization reactions
Source: Chem Sci. 2018 Jan 2;9(11):2898–908. doi: 10.1039/c7sc04430e (PMC5896375; doi:10.1039/c7sc04430e)
Supplement: Supplementary file 1 [file SC-009-C7SC04430E-s001.pdf]

Electronic Supplementary Material (ESI) for Chemical Science.
This journal is © The Royal Society of Chemistry 2018

Absolute and relative facial selectivities in organocatalytic asymmetric chlorocyclization reactions

Nastaran Salehi Marzijarani,[‡] Roozbeh Yousefi,[‡] Arvind Jaganathan,[†] Kumar Dilip Ashtekar,[‡] James E. Jackson,*[‡] and Babak Borhan*[‡]

[‡] Department of Chemistry, Michigan State University, East Lansing, MI 48824
[†]Dow AgroSciences LLC, 9330 Zionsville Road, Indianapolis, IN 46268

babak@chemistry.msu.edu, jackson@chemistry.msu.edu

Table of Contents:

I. General remarks: S3
II. General procedure for the catalytic asymmetric chlorocyclization of unsaturated amide **1b**: S4
III. Procedure for synthesis of unsaturated amide substrate **1b**: S5
IV. Labeling studies of 1,1-unsaturated amide: S7
 IV.a. Procedure for the synthesis of labeled amide substrate **1b-D**: S8
 IV.b1. Procedure for the non-catalyzed chlorocyclization of labeled unsaturated amide **1b-D**: S11
 IV.b2. Calculations to determine diastereomeric ratio of **2b-D** via non-catalyzed cyclization of **1b-D**: S12
 IV.b3. Effects of chlorine source, solvent and concentration on the diastereoselectivity of chlorocyclization of labeled amide **1b-D** in the absence of any catalyst: S14
 IV.b4. Changing the electronics of the aryl group and its effect on diastereoselectivity of non-catalyzed chlorocyclization of unsaturated amide **1b-D**: S16
 IV.c1. Procedure for the catalytic asymmetric chlorocyclization of labeled unsaturated amide **1b-D**: S20
 IV.c2. Calculations to determine the quantity of the four isomeric products **2b-D** obtained through $(DHQD)_2$PHAL catalyzed chlorocyclization: S22
V. Effect of other chlorine sources on the face selectivity of chlorenium attack and/or ring-closure: S27
VI. Absolute stereochemical assignment of C-6 configuration for **2b-D**: S28
 VI.a. General Procedure for the synthesis of labeled epoxy alcohols **3a** and **3b-D**: S29
VII. General procedure for the synthesis of carbamate substrate **1c**: S31
VIII. General procedure for the catalytic asymmetric chlorocyclization of carbamates in *n*-PrOH: S32
IX. General procedure for the catalytic asymmetric chlorocyclization of carbamates in $CHCl_3$-Hexane: S33
X. Procedure for synthesis of labeled carbamate substrate **1c-D**: S34

XI. Procedure for the non-catalyzed chlorocyclization of labeled unsaturated carbamate **1c-D**: S35
XII. Procedure for the catalytic asymmetric chlorocyclization of labeled unsaturated carbamate **1c-D**: S36
XIII. $(DHQD)_2$PHAL catalyzed chlorocyclization of **1c-D** monitored by ^{1}HNMR and HPLC: S36
XIV. Absolute stereochemical assignment at the deuterated center of substrates **2c-D** and *ent*-**2c-D**: S37
XV. General procedure for the synthesis of labeled epoxy sulfonamides *ent*-**3c** and *ent*-**3c-D**: S38
XVI. *E/Z* ratios of recovered labeled starting materials S42
XVII. Summaries of quantum chemical modeling (a) Uncatalyzed *Reaction B*: S44
XVIII. Summaries of quantum chemical modeling (b) $(DHQD)_2$PHAL-catalyzed *Reaction C*: S45
XIX. References: S46
XX. NMR spectra: S47

I. General remarks:

NMR spectra were obtained using a 300, 500, or 600 MHz NMR spectrometers (VARIAN INOVA). Chemical shifts are reported in parts per million (ppm) and are referenced using the residual ^{1}H peak from the deuterated solvent. For HRMS (ESI) analysis, a Waters 2795 (Alliance HT) instrument was used and referenced against Polyethylene Glycol (PEG-400-600).

Column chromatography was performed using Silicycle 60 Å, 35-75 μm silica gel. Pre-coated 0.25 mm thick silica gel 60 F254 plates were used for analytical TLC and visualized using UV light, iodine, potassium permanganate stain, *p*-anisaldehyde stain or phosphomolybdic acid in EtOH stain. TLC analyses were performed on silica gel plates (pre-coated on aluminum; 0.20 mm thickness with fluorescent indicator UV_{254}).

All reagents were purchased from commercial sources and used without purification unless otherwise mentioned. THF and Et_2O were freshly distilled from Na-benzophenone ketyl whereas CH_2Cl_2 and $PhCH_3$ were distilled over CaH_2. Trifluoroethanol was purchased from Aldrich or Alfa Aesar and used without further purification. Flash silica gel (32-63 µm) was used for column chromatography. Enantiomeric excess for all products was judged by HPLC analysis using DAICEL CHIRALPAK OJ-H or AD-H columns. Diastereomeric ratios were determined by crude NMR analysis. All known compounds were characterized by ^{1}H and ^{13}C NMR and are in complete agreement with samples reported elsewhere.

Abbreviations used: DCDMH = 1,3-dichloro-5,5-dimethylhydantoin; DCDPH = 1,3-

dichloro-5,5-diphenylhydantoin; TCCA = trichloroisocyanuric acid; DiCh.T = dichloramine-T; Ch.T = Chloramine-T; NCS = N-chlorosuccinimide; TLC = Thin layer chromatography; DCM = Dichloromethane; THF = Tetrahydrofuran; EtOAc = Ethyl Acetate; Hex = Hexane; *n*-PrOH = *n*-Propanol.

II. General procedure for the catalytic asymmetric chlorocyclization of unsaturated amide 1a:

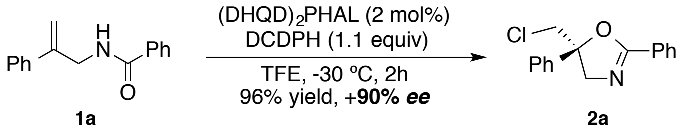

$(DHQD)_2PHAL$ (1.56 mg, 312 μL of a 5 mg/mL solution in TFE, 2 mol%) was introduced to a suspension of DCDPH (35 mg, 0.11 mmol, 1.1 equiv) in trifluoroethanol (TFE, 2.2 mL) in a screw capped vial equipped with a stir bar at -30 °C. After stirring vigorously for 10 min, the substrate (0.10 mmol, 1.0 equiv) was added in a single portion. The vial was capped and the stirring was continued at -30 °C till the reaction was complete (TLC). The reaction was quenched by the addition of 10% aq. Na_2SO_3 (3 mL) and diluted with DCM (3 mL). The organics were separated and the aqueous layer was extracted with DCM (3 x 3 mL). The combined organics were dried over Na_2SO_4, concentrated and purified by column chromatography on silica gel using EtOAc-Hexanes as the eluent to give product **2a** in 96% yield and 90% *ee*. Resolution of enantiomers: CHIRALCEL OJ-H, 5% IPA-Hexane, 0.8 mL/min, 260 nm, RT1 = 18.0 min, RT2 = 34.0 min. Analytical data for **2a**: ^1H NMR (500 MHz, $CDCl_3$) δ 8.05 (d, *J* =

7.0 Hz, 2H), 7.48 – 7.51 (m, 1H), 7.36 – 7.46 (m, 6H), 7.30 – 7.34 (m, 1H), 4.50 (d, *J* = 15.0 Hz, 1H), 4.23 (d, *J* = 15.0 Hz, 1H), 3.92 (d, *J* = 12.0 Hz, 1H), 3.85 (d, *J* = 12.0 Hz, 1H); ^{13}C NMR (125 MHz, $CDCl_3$) δ 163.0, 141.5, 131.5, 128.7, 128.4, 128.3, 128.2, 127.3, 124.9, 87.56, 64.93, 51.04. HRMS (ESI) Calculated Mass for $C_{16}H_{14}NOCl$: ([M+H]$^+$) = 271.0764, Found ([M+H]$^+$) = 271.0765.

III. Procedure for the synthesis of unsaturated amide substrate 1b:

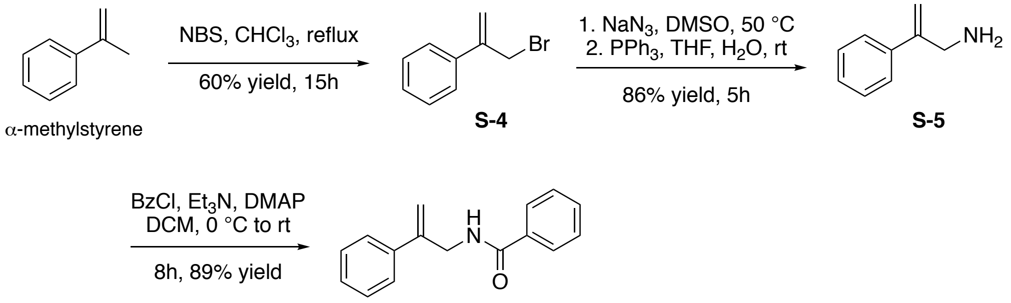

1b

S-4, (3-bromoprop-1-en-2-yl)benzene

NBS (8.58 g, 48.23 mmol, 1.14 equiv) was added to a solution of α-methylstyrene (5.0 g, 42.31 mmol, 1.0 equiv) in chloroform (7 mL) at room temperature. Reaction was refluxed for 15 h. After completion, the reaction mixture was filtered and concentrated to remove chloroform. Crude sample was subjected to column chromatography using hexane (100%) to afford **S-4** in 60% yield. Analytical data for **S-4**: ^{1}H NMR (500 MHz, $CDCl_3$) δ 7.27-7.39 (m, 5H), 5.54 (d, *J* = 1.2 Hz, 1H), 5.47 (d, *J* = 1.2 Hz, 1H), 4.37 (s, 2H); ^{13}C NMR (125 MHz, $CDCl_3$) δ 144.2, 137.6, 128.5, 128.3, 126.1, 117.2, 34.2.

S-5, 2-phenylprop-2-en-1-amine

Allyl bromide **S-4** (4.5 g, 22.96 mmol, 1.0 equiv) dissolved in THF (85 mL) and H_2O (21

mL), was treated with NaN_3 (1.79 g, 27.53 mmol, 1.2 equiv) at room temperature. After TLC analysis revealed the complete consumption of starting material (~ 3 h), PPh_3 (9.04 g, 34.46 mmol, 1.5 equiv) was added to the reaction vessel. After 2 h at ambient temperature, the reaction was concentrated to remove most of the THF. The resulting suspension was diluted with aq. HCl and extracted with ether (3x). The aqueous layer was then basified by adding solid KOH and extracted with ether (3x). The combined organics were dried (Na_2SO_4) and concentrated to give the crude amine, which was usually pure enough to use in the next step. Analytical data for **S-5**: ^{1}H NMR (500 MHz, $CDCl_3$) δ 7.27-7.40 (m, 5H), 5.33 (d, *J* = 1.2 Hz, 1H), 5.20 (d, *J* = 1.2 Hz, 1H), 3.70 (s, 2H); ^{13}C NMR (125 MHz, $CDCl_3$) δ 149.4, 139.5, 132.3, 131.5, 128.3, 111.0, 45.8.

1b, *N*-(2-phenylallyl)benzamide

A solution of 2-phenylprop-2-en-1-amine **S-5** (1.20 g, 9.0 mmol, 1.0 equiv), triethylamine (1.04 mL, 18.0 mmol, 2.0 equiv) and a catalytic amount of DMAP in DCM (50 mL) was cooled in an ice bath. To this solution, benzoyl chloride (1.57 mL, 13.5 mmol, 1.5 equiv) was added drop wise. After the completion of the reaction, it was allowed to warm to room temperature. It was quenched with water and extracted with DCM (3 x 25 mL). The combined organics were washed with brine (1 x 30 mL), dried over Na_2SO_4 and concentrated under reduced pressure to give the product as a colorless solid in 89% yield after column chromatography (10% EtOAc-Hexanes). Analytical data for **1b**: MP: 123 – 125 °C.; R_f: 0.46 (30% EtOAc-Hexane). ^{1}H NMR (500 MHz, $CDCl_3$) δ 7.7 (d, *J* = 8.5 Hz, 2H), 7.43-7.47 (m, 3H), 7.27-7.39 (m, 5H), 6.30 (br s,

1H), 5.49 (s, 1H), 5.29 (s, 1H), 4.52 (d, J = 6.0 Hz, 2H); ^{13}C NMR (125 MHz, $CDCl_3$) δ 167.3, 144.2, 138.3, 134.4, 131.5, 128.6, 128.5, 128.09, 126.9, 126.04, 114.0, 43.7. HRMS (ESI) Calculated Mass for $C_{16}H_{15}NO$: ($[M+H]^+$) = 237.1154, Found ($[M+H]^+$) = 237.1154.

IV. Labeling studies of 1,1-unsaturated amide:

To explore the stereocontrol in chlorocyclization reactions, we replaced one of the vinylic protons with deuterium. This leads to diastereotopic products with two stereogenic carbons, the quaternary carbon and the carbon attached to the chlorine (Scheme S1). Having two stereocenters on the final product provides the opportunity to investigate the face selectivity in the chlorination and the subsequent attack of the

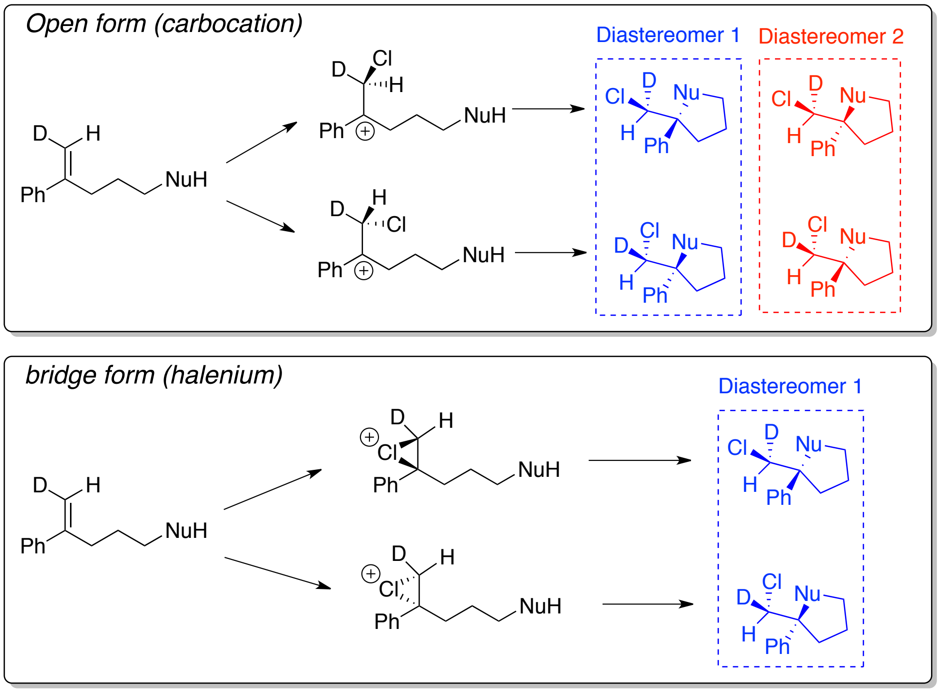

Scheme S1. Two plausible intermediates for a stepwise process: Open (forming two diastereomers) and bridged (forms one diastereomer) cations.

internal nucleophile. To execute this idea, the deuterated amide substrate **1b-D** was synthesized from 3-bromoprop-1-yne in 4 steps.

IV.a. Procedure for the synthesis of labeled amide substrate 1b-D:

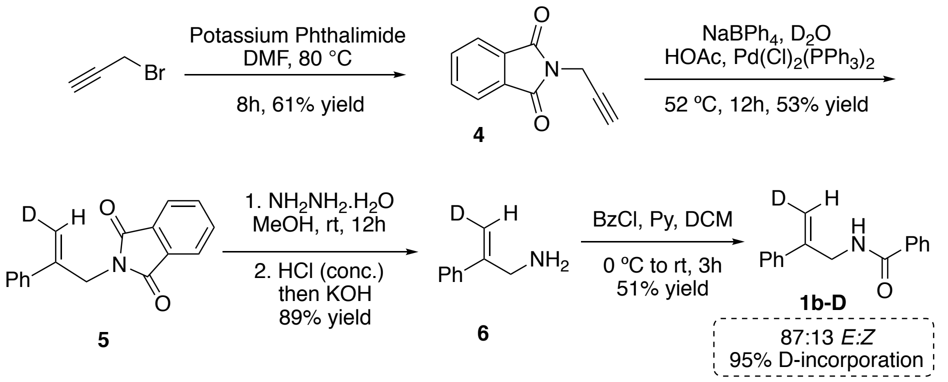

4, 2-(prop-2-yn-1-yl)isoindoline-1,3-dione

Potassium phthalimide (3.0 g, 16.2 mmol, 1.0 equiv) was added to a solution of 3-bromoprop-1-yne (2.8 mL of 80% wt. in toluene, 19.1 mmol, 1.18 equiv) in dry DMF (15 mL) at room temperature. After stirring the reaction at 80 °C for 8 h, it was poured into an ice cold water. The solid was filtered, washed with water and dried under vacuum. A white solid was obtained in 61% yield after recrystallization with DCM and Hexanes. Analytical data for **4**: ^{1}H NMR (500 MHz, $CDCl_3$) δ 7.89-7.85 (m, 2H), 7.70-7.74 (m, 2H), 4.44 (d, J = 2.5 Hz, 2H), 2.21 (t, J = 2.5 Hz, 1H); ^{13}C NMR (125 MHz, $CDCl_3$) δ 166.9, 134.2, 131.9, 123.5, 76.7, 71.5, 27.0.

5, Deuterated 2-(2-phenylallyl)isoindoline-1,3-dione[1]

A mixture of compound **4** (800 mg, 4.33 mmol, 1.0 equiv), $NaBPh_4$ (1.48 g, 4.33 mmol, 1.0 equiv), HOAc (247 µL, 4.33 mmol, 1.0 equiv), $PdCl_2(PPh_3)_2$ (91 mg, 0.13 mmol,

0.03 equiv), and D_2O (8.7 mL) was heated in a sealed tube under nitrogen at 50 °C for 12 h. After that, the solvents and volatiles were removed under reduced pressure and the mixture was subjected to column chromatography to afford compound **5** as a pale yellow powder in 53% yield. Analytical data for **5**: ^{1}H NMR (600 MHz, $CDCl_3$): δ7.82 (d, *J* = 6.0, 3.0 Hz, 2H), 7.68 (dd, *J* = 6.0, 3.0 Hz, 2H), 7.48 (dd, *J* = 7.8 Hz, 2H), 7.32 (m, 2H), 7.26 (t, *J* = 7.2 Hz, 1H), 5.12 (t, *J* = 1.8 Hz, 1H), 4.69 (d, *J* = 1.8 Hz, 2H), ^{13}C NMR (150 MHz, $CDCl_3$): δ 167.9, 142.3, 138.5, 133.9, 132.0, 128.4, 128.0, 126.4, 123.3, 113.6 (t, *J* = 23.7 Hz), 41.4. IR (cm^{-1}, NaCl plate): 2918, 2495, 1704; HRMS ($C_{17}H_{12}DNO_2$): Calc. $[M+H]^+$: 265.1088, Found $[M+H]^+$: 265.1086.

6, Deuterated 2-phenylprop-2-en-1-amine

Compound **5** (3.7 g, 18.9 mmol, 1.0 equiv) was dissolved in MeOH (40 mL). $NH_2NH_2.H_2O$ (1.7 mL) was introduced into the reaction vessel and the resulting suspension was stirred at room temperature overnight. The reaction was then diluted with water (100 mL) and most of MeOH was removed by rotary evaporation. Concentrated HCl (19 mL) was added and the resulting suspension was stirred for a further 60 min at ambient temperature. The precipitated solids were filtered and the filter cake was washed with water. The combined filtrates were basified with solid NaOH and extracted with EtOAc and concentrated to give deuterated 2-phenylprop-2-en-1-amine **6**, which was used in the next step without any purification. Analytical data for **6**: ^{1}H NMR (600 MHz, $CDCl_3$): δ 7.26-7.4 (1H, 5H), 5.19 (t, *J* = 1.8 Hz, 1H), 3.69 (s, 2H), 1.39 (br s, 2H); ^{13}C NMR (150 MHz, $CDCl_3$): δ 149.7, 139.7, 128.4, 127.6, 126.1, 110.9 (t, *J* = 24 Hz), 46.1. HRMS (ESI) Calculated Mass for $C_9H_{10}DN$: ($[M+H]^+$) =

135.1033, Found ([M+H]$^+$) = 135.1034.

1b-D, Deuterated *N*-(2-phenylallyl)benzamide

A solution of labeled 2-phenylprop-2-en-1-amine **6** (50 mg, 0.37 mmol, 1.0 equiv) and pyridine (63 mg, 0.74 mmol, 2.0 equiv) in DCM (2.5 mL) was cooled in an ice bath. To this, benzoyl chloride (80 mg, 0.57 mmol, 1.5 equiv.) was added drop wise. After the addition was complete, the reaction was allowed to warm to room temperature. After 3 h, the reaction was diluted with an equal amount of water and extracted with DCM (3 x 5 mL). The combined organics were washed with brine (1 x 3 mL), dried over Na_2SO_4 and concentrated under reduced pressure to give the product as a yellow solid. It was recrystallized from MeOH to obtain the pure product as a colorless solid in 51% yield [95% D-incorporated, (87:13) *E*:*Z* ratio of isomers]. Analytical data for **1b-D**: ^{1}H NMR (600 MHz, $CDCl_3$): δ 7.69 (d J = 8.4 Hz, 2H), 7.47 (m, 3H), 7.38 (t, J = 7.8 Hz, 2H), 7.34 (t, J = 7.2 Hz, 2H), 7.30 (t, J = 7.2 Hz, 1H), 6.15 (br s, 1H), 5.3 (t, J = 1.8 Hz, 1H), 4.52 (d, J = 5.4 Hz, 2H); ^{13}C NMR (150 MHz, $CDCl_3$): δ 167.3, 144.1, 138.2, 134.4, 131.4, 128.6, 128.5, 128.1, 126.8, 126.0, 113.7 (t, J = 24.0 Hz), 43.6; IR (cm^{-1}, NaCl plate): 3340, 2345, 1647. HRMS ($C_{16}H_{14}DNO$): Calc. [M+H]$^+$: 239.1294, Found [M+H]$^+$: 239.1297.

IV.b1. Procedure for the non-catalyzed chlorocyclization of labeled unsaturated amide 1b-D:

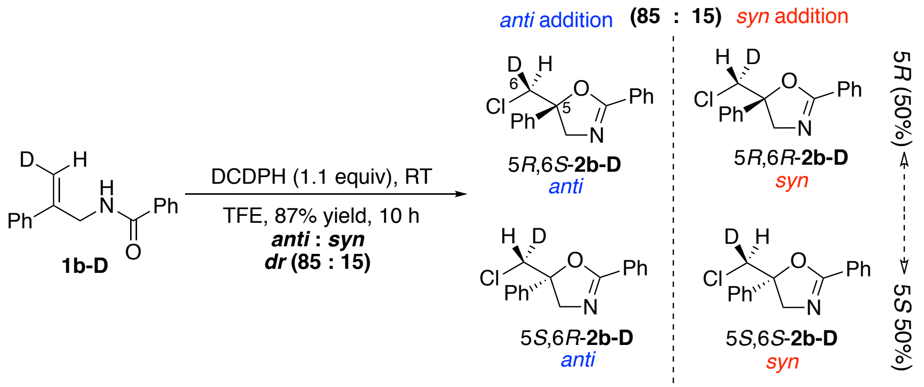

Substrate **1b-D** (24 mg, 0.10 mmol, 1.0 equiv) was added to a solution of DCDPH (35 mg, 0.11 mmol, 1.1 equiv) in TFE or $CHCl_3$ (2.2 mL) in a screw-capped vial equipped with a stir bar. After stirring vigorously for 10 h, the reaction was quenched by the addition of 10% aq. Na_2SO_3 (3 mL) and diluted with DCM (3 mL). The organics were separated and the aqueous layer was extracted with DCM (3 x 3 mL). The combined organics were dried over anhydrous Na_2SO_4. Pure products were isolated by column chromatography on silica gel using EtOAc-Hexanes (1:19) as the eluent to give the desired product **2b-D** in 87% yield as a mixture of two diastereomers (85:15 *dr*) (analyzed by ^{1}HNMR). Analytical data for **2b-D**: ^{1}H NMR (500 MHz, $CDCl_3$): δ 8.05 (dd, J = 1.5, 11.5 Hz, 2H), 7.32-7.50 (m, 8H), 4.49 (d, J = 15.0 Hz, 1H), 4.22 (d, J = 15.0 Hz, 1H), 3.91 (s, 1H); ^{1}H NMR (500 MHz, C_6D_6): δ 8.23 (m, 2H), 7.0-7.15 (m, 8H), 4.23 (d, J = 15.0 Hz, 1H), 3.98 (d, J = 15.0 Hz, 1H), 3.41 (s, 1H); ^{13}C NMR (125 MHz, $CDCl_3$): δ 163.0, 141.5, 131.6, 128.7, 128.4, 128.3, 128.2, 127.4, 124.9, 87.5, 64.9, 50.8 (t, J = 23.7 Hz). HRMS (ESI) Calculated Mass for $C_{16}H_{14}ONClD$: ($[M+H]^+$) = 273.0905, Found ($[M+H]^+$) = 273.0900.

IV.b2. Calculations to determine diastereomeric ratio of 2b-D via non-catalyzed cyclization of 1b-D:

The synthesized starting material **1b-D** contains 5% unlabeled amide **1b,** the integral value of each diastereomeric product was calculated by subtracting the integral value of the overlapping non-labeled product as depicted in Scheme S2. Peaks **a**, **b**, **c**, **d** (AB quartet) belongs to the non-labeled substrate. The integral values of peaks **a** and **d** are equivalent (**a** = **d** = 2.47) and so are the integral values for peaks **b** and **c** (**b** = **c** = 3.10). Peaks **e** and **f** belong to the deuterated product and overlap with **b** and **d**, respectively. To obtain the corrected value of **e**, the integral value of **c** is subtracted from the overall integral of **b** and **e** (**e** = 100 – 3.10 = 96.90). Similarly, to evaluate the integral of **f**, the integral value of **a** is subtracted from the overall integral value of **d** and **f** (**f** = 33.60 – 2.47 = 31.13). This calculation results in a 76:24 *anti*:*syn* ratio prior to 13% correction for the presence of the Z-olefin.

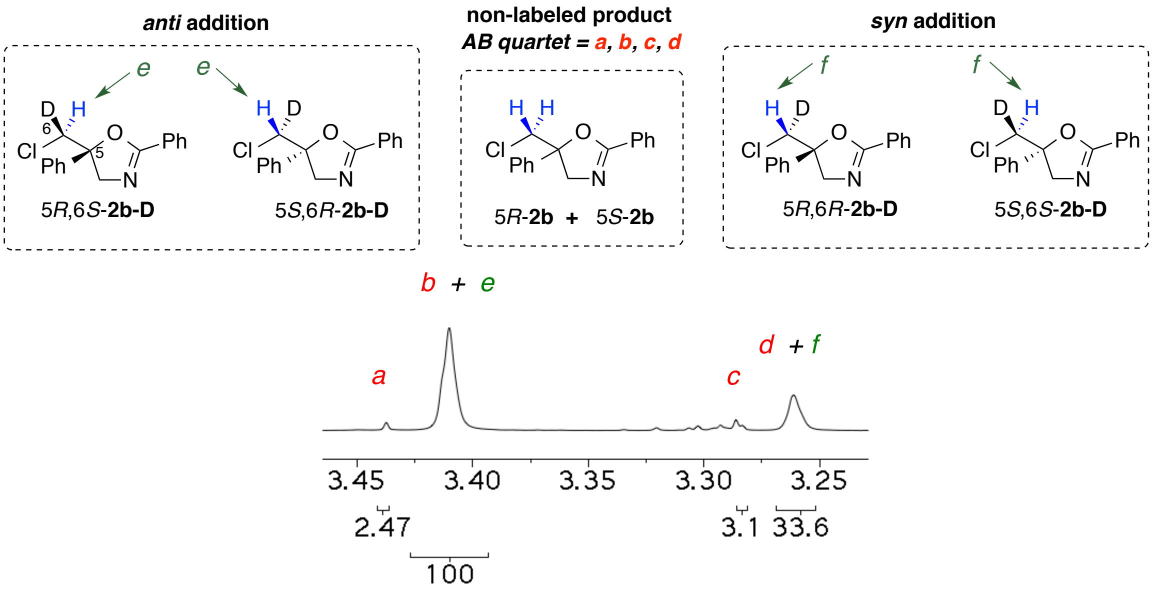

Scheme S2. ^{1}H NMR of labeled **2b-D**, cyclized under non-catalyzed condition.

For correction of the *E*/*Z* isomeric mixture, we used the following equations. Defining the fraction of product that arises from attack of Cl^+ on the olefin *syn* to the nucleophile as (**S**), and that *anti* to the nucleophile as (**A**), the following equations can be derived:

$$\left(\text{D1-}anti\right) = E\,\frac{\boldsymbol{S}}{(\boldsymbol{A}+\boldsymbol{S})} + Z\,\frac{\boldsymbol{A}}{(\boldsymbol{A}+\boldsymbol{S})} \qquad (i)$$

$$\left(\text{D2-}syn\right) = Z\,\frac{\boldsymbol{S}}{(\boldsymbol{A}+\boldsymbol{S})} + E\,\frac{\boldsymbol{A}}{(\boldsymbol{A}+\boldsymbol{S})} \qquad (ii)$$

After rearrangement and cross-multiplying, we get equation (*iii*). Since the dihydrogen corrected amount of *syn* and *anti* products are known (see correction discussed above), the corresponding values can be inserted in the below equation (*iii*) to evaluate the ratio of *anti*-addition to *syn*-addition for the final corrected value.

$$\%\,\frac{\text{D1}}{\text{D2}} = \frac{\left(\boldsymbol{S} \times Z\right) - \left(\boldsymbol{A} \times E\right)}{\left(\boldsymbol{A} \times Z\right) - \left(\boldsymbol{S} \times E\right)} \qquad (iii)$$

Replacing the numbers would give:

$$\%\,\frac{\text{D1}}{\text{D2}} = \frac{\left(31.13 \times 13\right) - \left(96.90 \times 87\right)}{\left(96.90 \times 13\right) - \left(31.13 \times 87\right)} = 5.54$$

Hence, %**D1** = 84.7 and %**D2** = 15.29. i.e. ***anti*-addition**: ***syn*-addition** = 85:15

This result suggests that the reaction does not go through a bridged chloronium intermediate, as two diastereomers resulting from *syn* and *anti* addition were observed in the absence of any catalyst. Other conditions such as changes in chlorenium source, solvent, and concentration were utilized to investigate the *syn*/*anti* diastereoselectivity.

IV.b3. Effects of chlorenium source, solvent and concentration on the diastereoselectivity of chlorocyclization of labeled amide 1b-D in the absence of any catalyst:

Most of the chlorenium sources result in diastereoselectivities in the range of 78:22 to 85:15 *anti*:*syn* cyclization except TCCA that gave 65:35 *dr,* nonetheless still favoring *anti* (Table S1, entries 1-8). Change of solvent from TFE to $CHCl_3$ or MeCN led to similar diastereoselectivity (Table S1, entries 1, 10-11). Other solvents such as DCM, toluene, and $CHCl_3$:Hex (1:1) yield more of the *anti*-cyclized product (Table S1, entries 9,12-13). Dilution plays an important role in the diastereoselectivity of this reaction. Decreasing the concentration of both substrate and DCDPH in TFE led to the formation of more *syn* product (Table S1, entries 14-20). Keeping the concentration of DCDPH constant (0.11 M), concentration of substrate in TFE was decreased from 0.10 M to 0.005 M (Table S1, entries 21-24). This led to a similar trend with an increase in the *syn* product. Conversely, when the concentration of substrate was kept constant at 0.01 M in TFE and concentration of DCDPH was lowered from 0.11 M (10.0 equiv) to 0.011 M (1.1 equiv), no significant changes were observed in diastereoselectivity (Table S1, entries 25-28).

Table S1. Screen of chlorenium source, solvents and effect of concentration on *syn*/*anti* ratio.

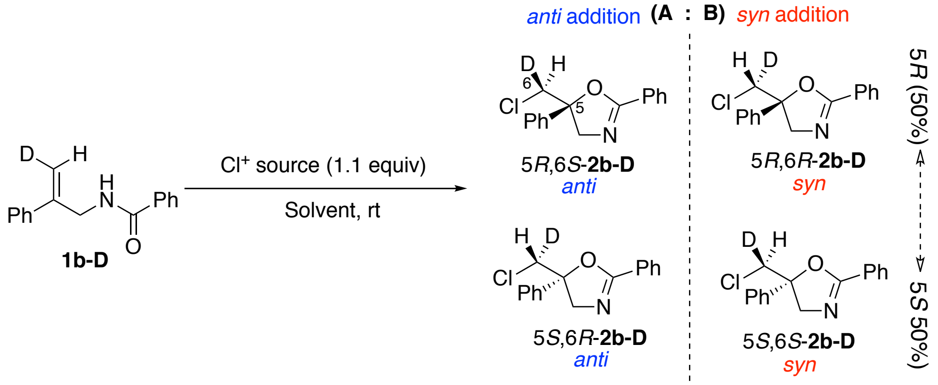

Entry	Cl^+ source	Solvent	[Sub]	$[Cl^+]$	time	*dr* (A:B) (*anti*:*syn*)
1	DCDPH	TFE	0.05 M	0.05 M	12 h	85:15
2	DCDMH	TFE	0.05 M	0.05 M	12 h	79:21
3	DiCh.T	TFE	0.05 M	0.05 M	12 h	90:10
4	N-Chlorosaccharin	TFE	0.05 M	0.05 M	12 h	86:14
5	TCCA	TFE	0.05 M	0.05 M	12 h	65:35
6	NCS	TFE	0.05 M	0.05 M	72 h	80:20
7[a]	N-Chlorophthalimide	TFE	0.05 M	0.05 M	72 h	78:22
8[b]	Ch.T	TFE	0.05 M	0.05 M	72 h	80:20
9	DCDPH	$CHCl_3$:Hex (1:1)	0.05 M	0.05 M	12 h	97:3
10	DCDPH	$CHCl_3$	0.05 M	0.05 M	12 h	85:15
11	DCDPH	MeCN	0.05 M	0.05 M	12 h	86:14
12	DCDPH	DCM	0.05 M	0.05 M	12 h	97:3
13	DCDPH	toluene	0.05 M	0.05 M	12 h	98:2
14	DCDPH	TFE	0.5 M	0.5 M	12 h	88:12
15	DCDPH	TFE	0.2 M	0.2 M	12 h	88:12
16	DCDPH	TFE	0.1 M	0.1 M	12 h	88:12
17	DCDPH	TFE	0.05 M	0.05 M	12 h	85:15
18	DCDPH	TFE	0.01 M	0.01 M	12 h	69:31
19	DCDPH	TFE	0.005 M	0.005 M	12 h	62:38

20	DCDPH	TFE	0.0025 M	0.0025 M	12 h	56:44
21	DCDPH	TFE	0.10 M	0.11 M	12 h	84:16
22	DCDPH	TFE	0.05 M	0.11 M	12 h	82:18
23	DCDPH	TFE	0.01 M	0.11 M	12 h	66:34
24	DCDPH	TFE	0.005 M	0.11 M	12 h	61:39
25	DCDPH	TFE	0.01 M	0.011 M	12 h	69:31
26	DCDPH	TFE	0.01 M	0.022 M	12 h	61:39
27	DCDPH	TFE	0.01 M	0.055 M	12 h	62:38

[a] 82% conversion after 3 days. [b] 58% conversion after 3 days.

IV.b4. Changing the electronics of the aryl group and its effect on the diastereoselectivity of non-catalyzed chlorocyclization of unsaturated amide 1a-D:

Electronic properties of the benzoyl group with the labeled amide substrate were varied to see the effect of nucleophilicity on the diastereoselectivity of this reaction. Both electron donating and withdrawing groups yield more *syn* product as compared to substrate **1b-D** (Table S2, entries 2-4).

Table S2. Effects of nucleophile on the *anti:syn* ratio.

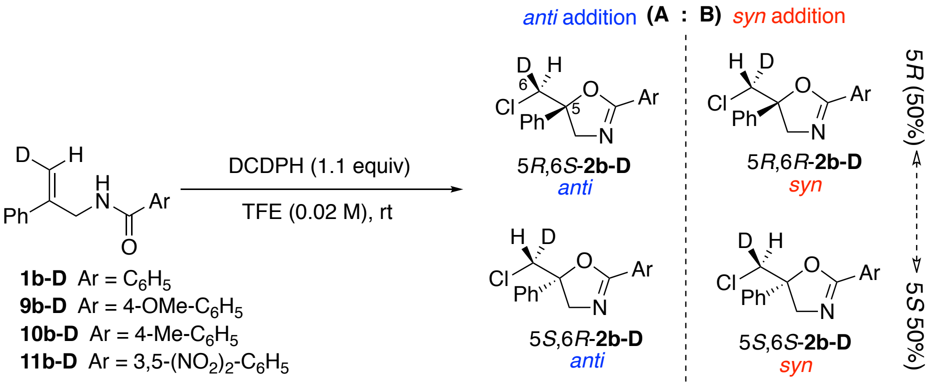

Entry	Substrate label	Product label (yb-D)	*dr* (A:B) (*anti*:*syn*)
1	**1b-D**, Ar = C_6H_5	**2b-D**	76:24
2	**9b-D**, Ar = 4-OMe-C_6H_5	**12b-D**	78:22
3	**10b-D**, Ar = 4-Me-C_6H_5-	**13b-D**	76:24
4	**11b-D**, Ar = 3,5-$(NO_2)_2$-C_6H_5	**14b-D**	57:43

General procedure for the synthesis of labeled substrates 9b-D to 11b-D: Substrates **9b-D** to **11b-D** were synthesized similarly to that described for substrate **1b-D** with the only difference being the choice of the amine protecting groups.

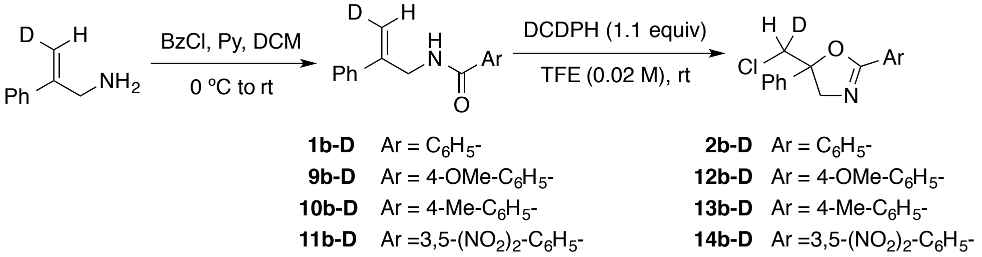

9b-D, Deuterated 4-methoxy-*N*-(2-phenylallyl)benzamide

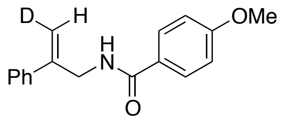

Analytical data for **9b-D**: ^{1}H NMR (500 MHz, $CDCl_3$) δ 7.66 (d, J = 8.5 Hz, 2H), 7.45 (d, J = 7.0 Hz, 2H), 7.34 (*t*, J = 7.0 Hz, 2H), 7.29 (d, J = 7.5 Hz, 1H), 6.87 (d, J = 9.0 Hz, 2H), 6.07 (br s, 1H), 5.28 (s, 1H), 4.50 (d, J = 5.5 Hz, 2H), 3.81 (s, 3H); ^{13}C NMR (125 MHz, $CDCl_3$) δ 166.8, 162.2, 144.3, 138.3, 128.7, 128.6, 128.1, 126.7, 126.1, 113.75, 113.70 (t, J = 22.0 Hz), 55.4, 43.7.

10b-D, Deuterated 4-methyl-*N*-(2-phenylallyl)benzamide

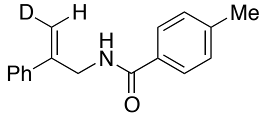

Analytical data for **10b-D**: ^{1}H NMR (500 MHz, $CDCl_3$) δ 7.59 (d, J = 8.0 Hz, 2H), 7.46 (d, J = 7.0 Hz, 2H), 7.33 (t, J = 6.5 Hz, 2H), 7.28 (d, J = 7.0 Hz, 1H), 7.17 (d, J = 8.0 Hz, 2H), 6.17 (br s, 1H), 5.28 (s, 1H), 4.51 (dd, J = 5.5 Hz, J = 1.0 Hz, 2H), 2.35 (s, 3H); ^{13}C NMR (125 MHz, $CDCl_3$) δ 167.2, 144.25, 141.9, 138.3, 131.6, 129.2, 128.6, 128.1, 126.9, 126.0, 113.7 (t, J = 24.0 Hz), 43.6, 21.4. HRMS (ESI) Calculated Mass for $C_{17}H_{16}DNO$: ($[M+H]^+$) = 253.1451, Found ($[M+H]^+$) = 253.1450.

11b-D, Deuterated 3,5-dinitro-*N*-(2-phenylallyl)benzamide

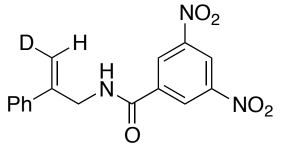

Analytical data for **11b-D**: ^{1}H NMR (500 MHz, $CDCl_3$) δ 9.10 (s, 1H), 8.86 (s, 2H), 7.42 (d, J = 7.5 Hz, 2H), 7.26-7.36 (m, 3H), 6.69 (br s, 1H), 5.31 (s, 1H), 4.57 (d, J = 5.5 Hz, 2H); ^{13}C NMR (125 MHz, $CDCl_3$) δ 162.7, 148.6, 143.2, 137.7, 137.6, 128.7, 128.4, 127.2, 126.0, 121.0, 114.8 (t, J = 24.0 Hz), 44.3.

General procedure for the non-catalyzed chlorocyclization of labeled 9a-D to 11b-D:

Substrates **12a-D** to **14b-D** were synthesized similarly to that described for substrate **2b-D**.

12b-D, Deuterated 5-(chloromethyl)-2-(4-methoxyphenyl)-5-phenyl-4,5-dihydrooxazole

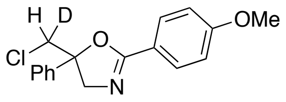

Analytical data for **12b-D**: ^{1}H NMR (500 MHz, C_6D_6) δ 8.20 (d, J = 8.5 Hz, 2H), 6.90-7.10 (m, 5H), 6.68 (d, J = 9.0 Hz, 2H), 4.22 (d, J = 15 Hz, 1H), 3.99 (d, J = 15 Hz, 1H), 3.42 (s, 1H), 314 (s, 3H); HRMS (ESI) Calculated Mass for $C_{17}H_{15}DNO_2Cl$: ([M+H]$^+$) = 303.1010, Found ([M+H]$^+$) = 303.1004.

13b-D, Deuterated 5-(chloromethyl)-5-phenyl-2-(*p*-tolyl)-4,5-dihydrooxazole

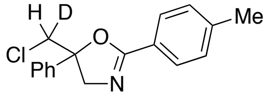

Analytical data for **13b-D**: ^{1}H NMR (500 MHz, $CDCl_3$) δ 7.94-7.97 (m, 2H), 7.39-7.45 (m, 3H), 7.34-7.37 (m, 1H), 7.26-7.28 (m, 3H), 4.51 (d, J = 14.5 Hz, 1H), 4.24 (d, J = 14.5 Hz, 1H), 3.93 (s, 1H), 2.42 (s, 3H); HRMS (ESI) Calculated Mass for $C_{17}H_{15}DNOCl$: ($[M+H]^+$) = 287.1061, Found ($[M+H]^+$) = 287.1057.

14b-D, Deuterated 5-(chloromethyl)-2-(3,5-dinitrophenyl)-5-phenyl-4,5-dihydrooxazole

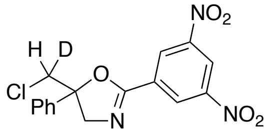

Analytical data for **14b-D**: ^{1}H NMR (500 MHz, $CDCl_3$) δ 9.20 (s, 3H), 7.37-7.45 (m, 5H), 4.63 (d, J = 15.5 Hz, 1H), 4.35 (d, J = 15.5 Hz, 1H), 3.96 (s, 1H).

IV.c1. Procedure for the catalytic asymmetric chlorocyclization of labeled unsaturated amide 1b-D:

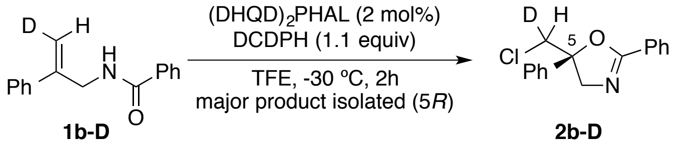

DCDPH (35 mg, 0.11 mmol, 1.1 equiv) was suspended in trifluoroethanol (TFE, 2.2 mL) in a screw-capped vial equipped with a stir bar. The resulting suspension was

cooled to -30 °C in an immersion cooler. $(DHQD)_2PHAL$ (1.56 mg, 312 mL of a 5 mg/mL solution in TFE, 2 mol%) was then introduced. After stirring vigorously for 10 min, unsaturated amide **1b-D** (24 mg, 0.10 mmol, 1.0 equiv) was added in a single portion. The vial was capped and the stirring was continued at -30 °C till the reaction was complete (TLC). The reaction was quenched by the addition of 10% aq. Na_2SO_3 (3 mL) and diluted with DCM (3 mL). The organics were separated and the aqueous layer was extracted with DCM (3 x 3 mL). The combined organics were dried over anhydrous Na_2SO_4. The pure product was isolated by column chromatography on silica gel using EtOAc-Hexanes (1:19) as the eluent in 93% yield. The resulting two enantiomers (ignoring the stereochemistry at the labeled carbon (C-6) as it is not distinguishable by chiral HPLC) were separated using a chiral pack OJ-H column (5% IPA in hexanes; 0.8 mL/min; 265 nm; RT_1 = 17.9 (*S* enantiomer) and RT_2 = 34.3 (*R* enantiomer)) and an *R* to *S* ratio *of* 93:7 was obtained. Analytical data for **2b-D**: ^1H NMR (500 MHz, $CDCl_3$): δ 8.05 (dd, *J* = 1.5, 11.5 Hz, 2H), 7.32-7.50 (m, 8H), 4.49 (d, *J* = 15.0 Hz, 1H), 4.22 (d, *J* = 15.0 Hz, 1H), 3.91 (s, 1H); ^1H NMR (500 MHz, C_6D_6): δ 8.23 (m, 2H), 7.0-7.15 (m, 8H), 4.23 (d, *J* = 15.0 Hz, 1H), 3.98 (d, *J* = 15.0 Hz, 1H), 3.41 (s, 1H); ^{13}C NMR (125 MHz, $CDCl_3$): δ 163.0, 141.5, 131.6, 128.7, 128.4, 128.3, 128.2, 127.4, 124.9, 87.5, 64.9, 50.8 (t, *J* = 24.0 Hz). HRMS (ESI) Calculated Mass for $C_{16}H_{14}ONClD$: ($[M+H]^+$) = 273.0905, Found ($[M+H]^+$) = 273.0900.

IV.c2. Calculations to determine the quantity of the four isomeric products 2b-D obtained through $(DHQD)_2PHAL$ catalyzed chlorocyclization:

$(DHQD)_2PHAL$ catalyzed chlorocyclization of labeled amide **1b-D** was investigated. The high enantioselectivity of the final cyclized product must be the result of high facial selectivity of the ring-closing event. However, understanding the enantioselectivity at both alkene ends is important to fully comprehend the role of the chiral catalyst. Desymmetrization of dichlorohydantoin by binding to the cinchona alkaloid catalyst *in the absence of substrate* is observable by NMR.[2] But with 1,1-disubstituted alkene substrates, the pi face selectivity of chlorenium delivery is not evident from the products formed, in which the chlorine atom resides on a non-stereogenic carbon. To measure the face selectivity of the chlorenium attack, deuterated substrate **1b-D** was utilized. Reaction of the labeled substrate under optimized asymmetric catalytic conditions results in products that enable analysis of olefin face selectivity for chlorenium delivery and ring closure via chiral HPLC and ^{1}H NMR spectroscopy (see Scheme S3).[2b]

In order to obtain the ratio of four isomeric products, two groups of products epimeric at C5 (5*R* versus 5*S*) were separated by chiral HPLC [with (93:7) *er* favoring *R* configuration at C-5 as determined by crystallography].[3] Since HPLC does not separate deuterated from non-deuterated analogs, we obtained D1En2/D2En2 (set1) and D1En1/D2En1 (set2) after HPLC purification using OJ-H chiral column (Scheme S3). Set1 contains two diastereomers (5*R*,6*R*) and (5*R*,6*S*), while set2 has diastereomers (5*S*,6*S*) and (5*S*,6*R*). Note that each set contains epimeric C6 compounds, reflecting

the diastereomers engendered by the deuterium labels. ^{1}H NMR analysis of these two sets reveal a *dr* of (99:1) for set1 (obtained from chiral HPLC separation of the major enantiomer at C-5), and a *dr* of (73:27) for set2 (obtained from chiral HPLC separation of the minor enantiomer). These diastereomeric ratios are the finalized values after correction for the amount of non-labeled product and *E*/*Z* mixture of the starting material. The corrections are described below.

Steps toward the calculation of the final corrected *dr* numbers are as follows. The *dr* ratios were calculated by subtracting the integral value of the non-labeled product from the overall integral of the diastereomers as explained earlier for the non-catalyzed chlorocyclization analysis. The ^{1}H NMR of the starting material **1b-D** shows 13% *Z* olefin as contaminant. For correction of the *E*/*Z* isomeric mixture, we used the following equations. Defining the fraction of product that arises from C6-Pro-*S* attack of Cl^+ on the olefin as **B** (*bottom face attack*), and that from the C6-Pro-*R* attack of Cl^+ as **T** (*top face attack*), the following equations can be derived:

$$\left(5R,\ 6R\right) = E\ \frac{\boldsymbol{B}}{(\boldsymbol{T}+\boldsymbol{B})} + Z\ \frac{\boldsymbol{T}}{(\boldsymbol{T}+\boldsymbol{B})} \qquad (iv)$$

$$\left(5R,\ 6S\right) = Z\ \frac{\boldsymbol{B}}{(\boldsymbol{T}+\boldsymbol{B})} + E\ \frac{\boldsymbol{T}}{(\boldsymbol{T}+\boldsymbol{B})} \qquad (v)$$

After rearrangement and cross-multiplying, we get equation (*vi*). Since the diastereomeric ratio is available from ^{1}H NMR analysis, the corresponding values can be inserted in equation (*vi*) to evaluate the ratio of *syn*-addition to *anti*-addition for the final corrected value.

$$\% \frac{\boldsymbol{B}}{\boldsymbol{T}} = \frac{(5R, 6S) \times Z - (5R, 6R) \times E}{(5R, 6R) \times Z - (5R, 6S) \times E} \qquad (vi)$$

For an example, lets walk through the calculation of *anti*:*syn* (D1:D2) ratio of the major enantiomer (En2) using ^{1}H NMR analysis [D1En2/D2En2 (set1), shown in Scheme S3]. The synthesized starting material **1b-D** contains 5% unlabeled amide **1b,** the integral value of each diastereomer was calculated by subtracting the integral value of the overlapping non-labeled product as depicted in Scheme S4. Peaks **a**, **b**, **c**, **d** (AB quartet) belongs to the non- labeled substrate. The integral values of peaks **a** and **d** are equivalent (**a** = **d** = 2.05) and so are the integral values for peaks **b** and **c** (**b** = **c** = 3.60). Peaks **e** and **f** belong to the deuterated product and overlap with **b** and **d**, respectively. To obtain the corrected value of **e**, the integral value of **c** is subtracted from the overall integral of **b** and **e** (**e** = 100 – 3.60 = 96.4). Similarly to evaluate the integral of **f**, the integral value of **a** is subtracted from the overall integral value of **d** and **f** (**f** = 16.49 – 2.05 = 14.44). This calculation results in 87:13 *anti*:*syn* ratio prior to 13% correction for the *Z*-olefin.

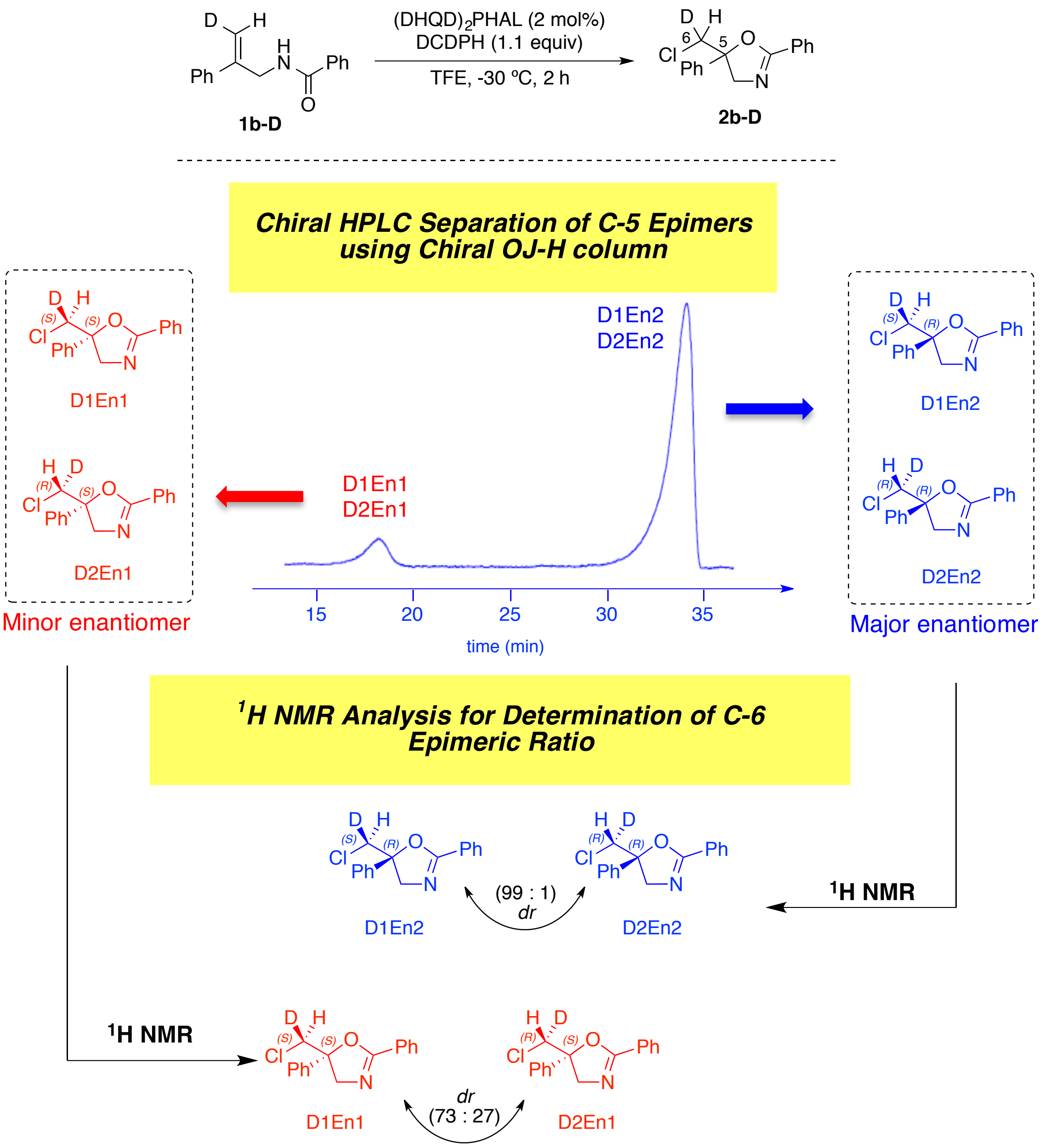

Scheme S3. Face selectivity in chlorine delivery was measured by using chiral HPLC and ^{1}H NMR.

Assuming similar stereoselectivity of *E* and *Z* isomers, the major diastereomer (*anti*) (5*R*,6*S*) product [which was found to be *anti* diastereomer through derivatization studies], results from bottom face (**B**) attack of Cl^+ (C6-Pro-*S* attack) on the *E* olefin (major contribution) and top face (**T**) attack of Cl^+ (C6-Pro-*R* attack) onto the *Z* olefin

(minor contribution). Similarly, the minor diastereomer (*syn*) (5*R*,6*R*), results from top face (**T**) attack of Cl^{+} on the *E* olefin (major contribution) and bottom face (**B**) attack of Cl^{+} on *Z* olefin (minor contribution). In order to correct for 13% *Z* olefin impurity, we used equation (*vi*):

$$\%\ \frac{\boldsymbol{B}}{\boldsymbol{T}} = \frac{(14.44 \times 13) - (96.40 \times 87)}{(96.40 \times 13) - (14.44 \times 87)} = 2662.04$$

Hence, %**B** = 99.96 and %**T** = 0.04. i.e. ***anti*-addition**:***syn*-addition** = >99:1

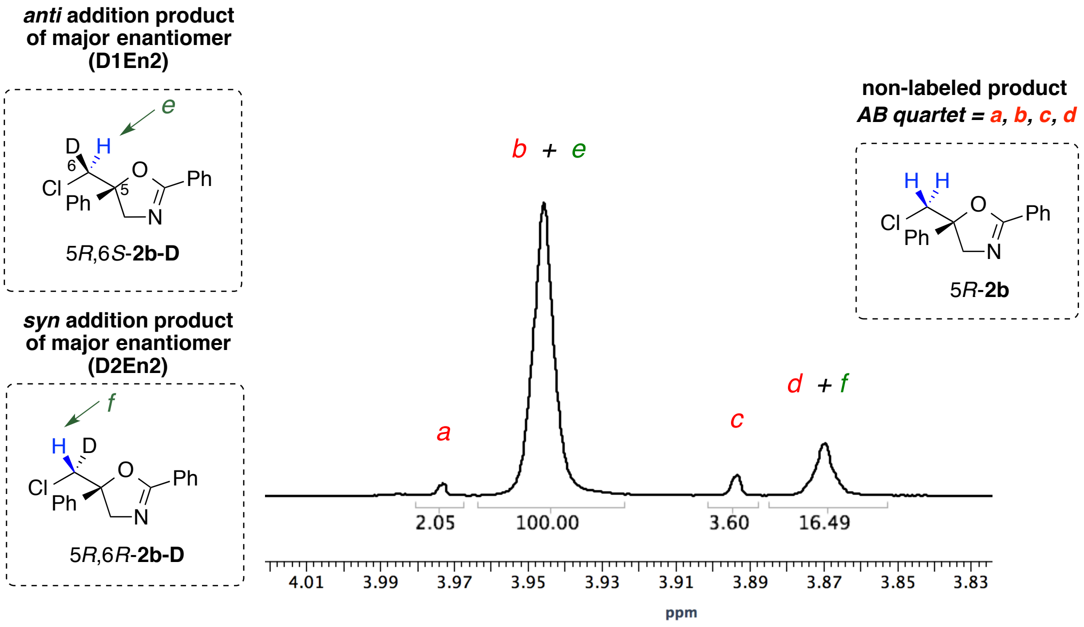

Scheme S4. ^{1}H NMR spectrum of the major enantiomer 5*R*-**2b-D**, cyclized under catalyzed condition.

V. Effect of other chlorine sources on the face selectivity of chlorenium attack and/or ring-closure

Dichloramine.T salt is as effective as DCDPH in chlorocyclization of unsaturated amide **1b-D** yielding the *anti* product 5*R*,6*S*-**2b-D** in 91% yield and 92:8 *er*. The ratio of each isomer is shown in Scheme S5 (top). Replacing DCDPH with TCCA, however results in a significant drop in enantioselectivity apparent from loss of C-5 selectivity (Scheme S5, bottom). The facial selectivity in chlorenium delivery suffers also, albeit not to the same extent (82:18).

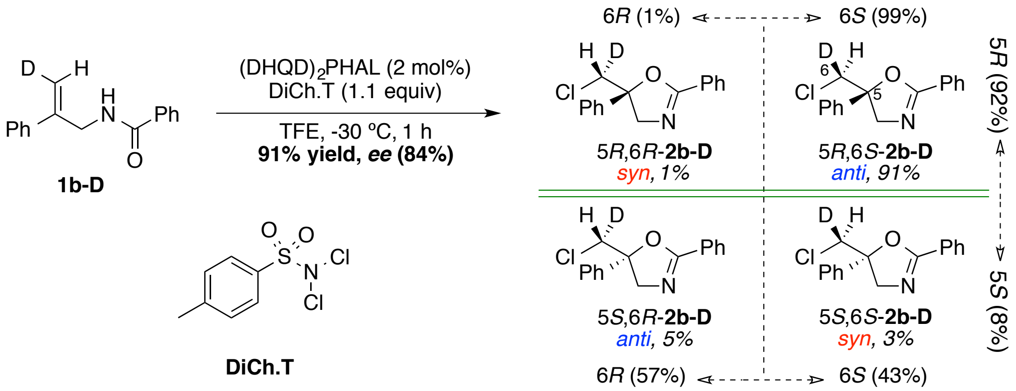

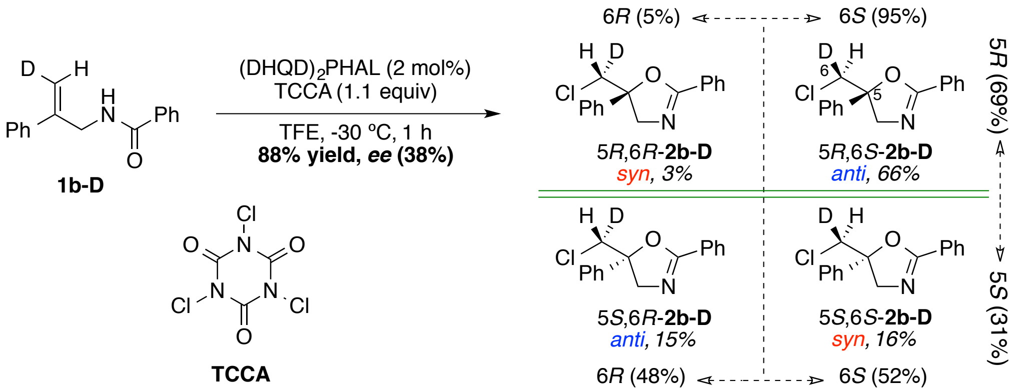

Scheme S5. Change of chlorine source to DichT (top) and TCCA (bottom) and their effects on ratio of four isomers.

VI. Absolute stereochemical assignment of C-6 configuration for 2b-D:

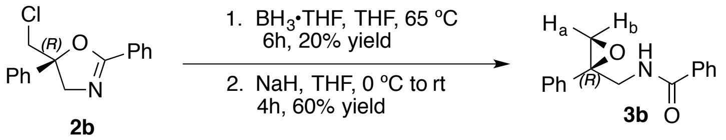

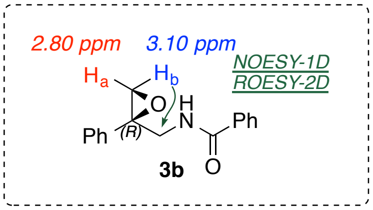

Borohydride reduction of oxazoline **2b** followed by sodium hydride mediated cyclization of the resulting chlorohydrin intermediate returned 1,1-disubstituted epoxy amide **3b**. ROESY and NOESY studies on the corresponding epoxy amide **3b** show that H_a (2.80 ppm) has a *syn* orientation with the phenyl group and H_b (3.10 ppm) has an *anti* orientation with the phenyl group. After assignment of H_a and H_b, the labeled substrate **2b-D** as a mixture (synthesized in the presence of the $(DHQD)_2PHAL$) was reduced with BH_3.THF, followed by base treatment to yield the desired labeled epoxy amide **3b-D**. ^1H NMR analysis of the epoxy amide **3b-D** shows only a peak at 3.10 ppm, which suggests that the deuterium has a *cis* orientation with respect to the phenyl group. Based on this finding, the deuterated carbon is assigned as the *R* configuration in the corresponding epoxy amide **3b-D**. Since the epoxy amide is formed through the S_N2

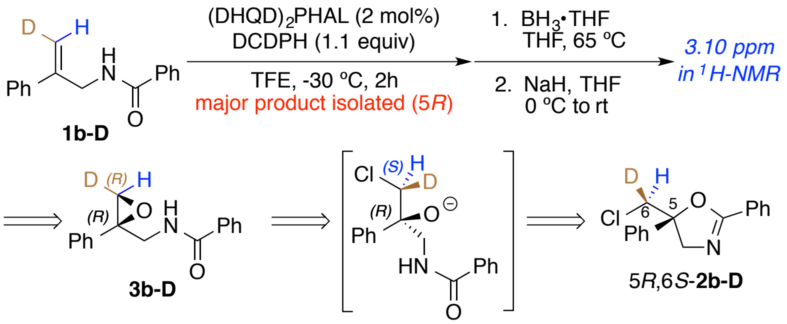

opening of the corresponding chlorohydrin intermediate, amide **2b-D** (major product) is assigned the *S* configuration at C-6.

VI.a. General Procedure for the synthesis of epoxy alcohols 3b and 3b-D:

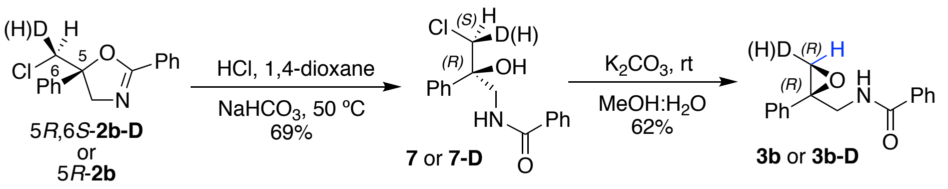

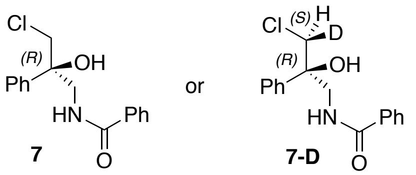

Oxazoline **2b** or **2b-D** (127 mg, 0.47 mmol, 1.0 equiv, 93:7 *er* after chiral chromatographic separation) was placed in a pre-dried flask. 1,4-Dioxane (10 mL) and 1*N* hydrochloric acid (10 mL) were added to the flask. The resulting mixture was stirred for 4 h at 50 °C. After it was cooled to room temperature, the reaction mixture was neutralized with saturated $NaHCO_3$ and subsequently extracted six times with EtOAc. The combined organic layers was dried over Na_2SO_4 and the solvent was evaporated under reduced pressure. Column chromatography of the crude isolate gave the product **7** (94 mg) or **7-D** in 69% yield. Analytical data for **7**: ^{1}H NMR (500 MHz, $CDCl_3$) δ 7.63 (d, *J* = 8.0 Hz, 2H), 7.50 (d, *J* = 7.5 Hz, 2H), 7.46 (t, *J* = 7.0 Hz, 1H), 7.34-7.35 (m, 4H), 7.30 (t, *J* = 7.0 Hz, 1H), 6.50 (br s, 1H), 4.11 (dd, *J* = 14.5 Hz, *J* = 7.0 Hz, 1H), 3.92 (d, *J* = 11.5 Hz, 1H), 3.85 (d, *J* = 11.5 Hz, 1H), 3.75 (dd, *J* = 14.0 Hz, *J* = 5.5 Hz, 1H); ^{13}C NMR (125 MHz, $CDCl_3$) δ 168.8, 141.0, 133.8, 131.8, 128.6, 128.0, 126.9, 125.5, 76.7,

52.0, 48.0. HRMS (ESI) Calculated Mass for $C_{16}H_{16}NO_2Cl$: ($[M+H]^+$) = 290.0948, Found ($[M+H]^+$) = 290.0948. Analytical data for **7-D**: ^{1}H NMR (500 MHz, $CDCl_3$) δ 7.64 (d, *J* = 8.0 Hz, 2H), 7.50 (d, *J* = 7.0 Hz, 2H), 7.45 (t, *J* = 7.0 Hz, 1H), 7.35-7.39 (m, 4H), 7.30 (t, *J* = 7.0 Hz, 1H), 6.51 (br s, 1H), 4.12 (dd, *J* = 14.5 Hz, *J* = 6.5 Hz, 1H), 3.91 (s, 1H), 3.74 (dd, *J* = 14.0 Hz, *J* = 5.5 Hz, 1H); ^{13}C NMR (125 MHz, $CDCl_3$) δ 168.8, 141.0, 133.7, 131.8, 128.5, 128.0, 126.9, 125.5, 76.7, 51.8 (t, J = 23.0 Hz), 48.0. HRMS (ESI) Calculated Mass for $C_{16}H_{15}DClNO_2$: ($[M+H]^+$) = 291.1010, Found ($[M+H]^+$) = 291.1011.

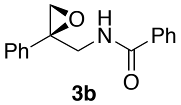

3b

or

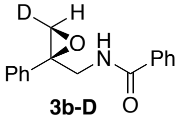

3b-D

K_2CO_3 (7.2 mg, 0.052 mmol, 1.5 equiv) was added to a solution of **3b** or **3b-D** (10 mg, 0.035 mmol, 1.0 equiv) in MeOH:H_2O (10:1, 1.0 mL) at 0 °C and then warmed to room temperature. After completion of the reaction, monitored by TLC, MeOH was removed under reduced pressure. The remaining aqueous suspension was extracted with EtOAc and H_2O. The organics were dried over Na_2SO_4 and the solvent was removed under reduced pressure. The crude product was purified with column chromatography to yield **3b** (5.5 mg) or **3b-D** in 62% yield. Analytical data for **3b**: ^{1}H NMR (500 MHz, $CDCl_3$) δ 7.71 (d, *J* = 7.0 Hz, 2H), 7.48 (t, *J* = 7.5 Hz, 1H), 7.25-7.44 (m, 7H), 6.29 (br s, 1H), 4.22 (dd, *J* = 14.5 Hz, *J* = 7.0 Hz, 1H), 4.01 (dd, *J* = 14.5 Hz, *J* = 4.5 Hz, 1H), 3.11 (d, *J* = 5.0 Hz, 1H), 2.81 (d, J = 5.0 Hz, 1H). HRMS (ESI) Calculated Mass for $C_{16}H_{15}NO_2$: ($[M+H]^+$) = 254.1181, Found ($[M+H]^+$) = 254.1178. Analytical data

for **3b-D**: ^{1}H NMR (500 MHz, $CDCl_3$) δ 7.75 (d, *J* = 7.5 Hz, 2H), 7.22-7.56 (m, 8H), 6.39 (br s, 1H), 4.27 (dd, *J* = 14.5 Hz, *J* = 7.0 Hz, 1H), 4.06 (dd, *J* = 14.5 Hz, *J* = 5.0 Hz, 1H), 3.14 (s, 1H). HRMS (ESI) Calculated Mass for $C_{16}H_{14}DNO_2$: ($[M+H]^+$) = 255.1244, Found ($[M+H]^+$) = 255.1252.

VII. General procedure for the synthesis of carbamate substrate 1c:

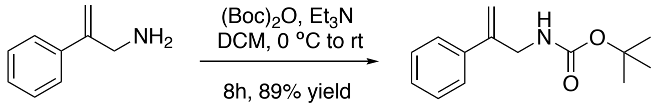

S-5 **1c**

A solution of di-*tert*-butyl dicarbonate (0.71 g, 3.14 mmol, 1.1 equiv) in dry DCM (1 mL) was added under nitrogen at 0 °C to a solution of amine **S-5** (0.5 g, 2.85 mmol, 1.0 equiv) and triethylamine (0.87 mL, 6.27 mmol, 2.2 equiv) in dry DCM (2 mL). The reaction mixture was stirred at room temperature for 24 h. The solvent was removed in *vacuo*, and the residue was dissolved with DCM (6 mL) and water (4 mL). The aqueous layer was extracted with DCM (3 × 5 mL). The combined organic extracts were washed with water (5 mL), dried with $MgSO_4$, filtered, and concentrated. The resulting mixture was purified by flash chromatography on silica gel using 10% EtOAc-hexane to give carbamate **1c** (*tert-butyl (2-phenylallyl)carbamate)* in 89% yield. Analytical data for **1c**: ^{1}H NMR (500 MHz, $CDCl_3$) δ 7.40 (d, *J* = 7.5 Hz , 2H), 7.30-7.38 (m, 2H), 7.25-7.29 (m, 1H), 5.41 (s, 1H), 5.21 (s, 1H), 4.61 (br s, 1H), 4.17 (d, *J* = 5.0 Hz, 2H), 1.42 (s, 9H); ^{13}C NMR (125 MHz, $CDCl_3$) δ [illegible] 144.8, 138.6, 128.4,

127.9, 126.1, 112.9, 79.4, 44.3, 28.4. HRMS (ESI) Calculated Mass for $C_{14}H_{19}NO_2$: ($[M+Na]^+$) = 256.1313, Found ($[M+Na]^+$) = 256.1322.

VIII. General procedure for the catalytic asymmetric chlorocyclization of carbamates in *n*-PrOH:

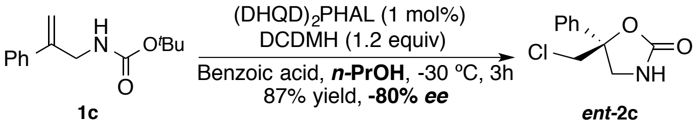

A screw-capped vial equipped with a stir bar was charged with 1.3 mL of a 0.21 mg/mL stock solution of $(DHQD)_2PHAL$ in *n*-PrOH [0.30 mg $(DHQD)_2PHAL$, 1 mol%]. After cooling to -30 °C in an immersion cooler**,** DCDMH (9.5 mg, 0.041 mmol, 1.3 equiv) and benzoic acid (2.3 mg, 0.019 mmol, 0.5 equiv) were added sequentially. After stirring vigorously for 10 min, the substrate (0.037 mmol, 1.0 equiv) in *n*-PrOH (0.2 mL, pre-cooled to reaction temperature) was added in a single portion. The vial was capped and the stirring was continued at -30 °C until the reaction was complete as judged by TLC. The reaction was quenched by the addition of 2% aq. NaOH (3 mL) and diluted with DCM (3 mL). The organics were separated and the aqueous layer was extracted with DCM (3 x 3 mL). The combined organics were dried over anhydrous Na_2SO_4 and concentrated in the presence of a small quantity of silica gel. Pure product *ent*-**2c** (*5-(chloromethyl)-5-phenyloxazolidin-2-one*) was isolated by column chromatography on a short silica gel column using EtOAc-hexane as the eluent (87% yield). The resulting two enantiomers were separated using a chiral pack AD-H column (10% IPA in

hexanes; 1.0 mL/min. Analytical data for *ent*-**2c**: ^{1}H NMR (500 MHz, $CDCl_3$) δ 7.33-7.42 (m, 5H), 5.77 (br s, 1H), 4.11 (d, J = 9.0 Hz, 1H), 3.84 (d, J = 12.0 Hz, 1H), 3.79 (d, J = 8.5 Hz, 1H), 3.75 (d, J = 12.0 Hz, 1H); ^{13}C NMR (125 MHz, $CDCl_3$) δ 158.1, 139.8, 128.9, 128.8, 124.7, 83.8, 50.7, 49.4. HRMS (ESI) Calculated Mass for $C_{10}H_{10}ClNO_2$:([M+H]$^+$) = 212.0478, Found ([M+H]$^+$) = 212.0479.

IX. General procedure for the catalytic asymmetric chlorocyclization of carbamates in $CHCl_3$-Hexane:

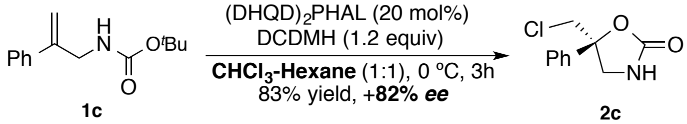

1c **2c**

$(DHQD)_2$PHAL (6 mg, 20 mol%) was introduced to a suspension of DCDMH (9.5 mg, 0.041 mmol, 1.3 equiv) in $CHCl_3$-hexane (1:1 mixture, 1.3 mL) in a screw-capped vial equipped with a stir bar. The resulting suspension was cooled to 0 °C in an immersion cooler. After stirring vigorously for 10 min, the substrate (0.037 mmol, 1.0 equiv) in $CHCl_3$-hexane (1:1 mixture, 0.2 mL, pre-cooled to reaction temperature) was added in a single portion. The vial was capped and the stirring was continued at 0 °C until the reaction was complete (TLC). The reaction was quenched by the addition of 2% aq. NaOH (3 mL) and diluted with DCM (3 mL). The organics were separated and the aqueous layer was extracted with DCM (3 x 3 mL). The combined organics were dried over anhydrous Na_2SO_4 and concentrated in the presence of a small quantity of silica gel. Pure products were isolated by column chromatography on a short silica gel

column using EtOAc-hexane (20-50%) as the eluent. Analytical data is similar to the part VIII.

X. Procedure for the synthesis of labeled carbamate substrate 1c-D:

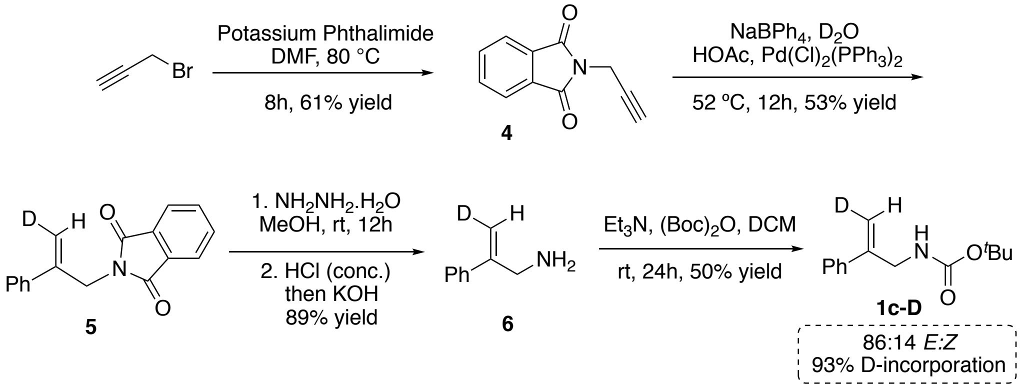

Synthesis of compound **6** was shown previously. Boc-protection of the primary amine **6** to provide **1c-D** was carried out similar to the procedure for synthesis of non-labeled carbamate **1c**. The desired compound **1c-D** was synthesized in 50% yield with 86:14 *E*:*Z* ratio and 93% deuterium incorporation. Analytical data for **1c-D**: ^{1}H NMR (500 MHz, $CDCl_3$) δ 7.40 (d, J = 7.0 Hz, 2H), 7.25-7.34 (m, 3H), 5.2 (t, J = 1.5 Hz, 1H), 4.61 (br s, 1H), 4.17 (d, J = 5.0 Hz, 2H), 1.42 (s, 9H); ^{1}H NMR (500 MHz, C_6D_6) δ 7.30 (d, J = 7.0 Hz, 2H), 7.01-7.20 (m, 3H), 5.00 (s, 1H), 4.18 (br s, 1H), 4.07 (d, J = 5.5 Hz, 2H), 1.46 (s, 9H); ^{13}C NMR (125 MHz, $CDCl_3$) δ 155.7, 144.9, 138.7, 128.5, 127.9, 126.1, 112.9 (t, J = 25.0 Hz), 79.4, 44.4, 28.4. HRMS (ESI) Calculated Mass for $C_{14}H_{18}DNO_2$: ($[M+Na]^+$) = 257.1376, Found ($[M+Na]^+$) = 257.1380.

XI. Procedure for the racemic chlorocyclization of labeled unsaturated carbamate 1c-D:

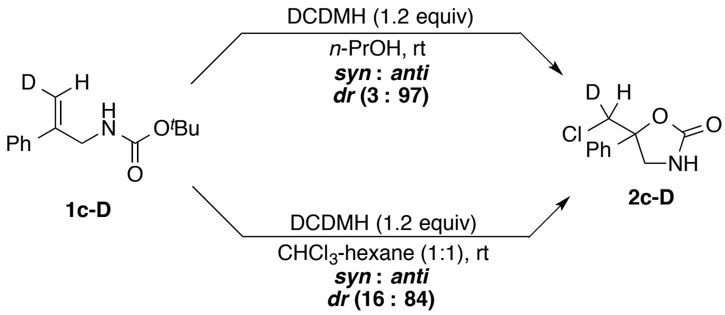

Substrate **1c-D** (15 mg, 0.064 mmol, 1.0 equiv) was added to a solution of DCDMH (15 mg, 0.072 mmol, 1.2 equiv) in $CHCl_3$:Hexane (1:1) (2.5 mL) or *n*-PrOH (2.5 mL) in a screw-capped vial equipped with a stir bar. After stirring vigorously for 12 h, the reaction was quenched by the addition of 2% aq. NaOH (3 mL) and diluted with DCM (3 mL). The organics were separated and the aqueous layer was extracted with DCM (3 x 3 mL). The combined organics were dried over Na_2SO_4 and the solvent was removed under reduced pressure. Pure products were isolated by column chromatography on silica gel using EtOAc-Hexanes as the eluent to give the desired product **2c-D** as a mixture of two diastereomers (97:3 *dr*) and (84:16) for reactions carried out in *n*-PrOH and $CHCl_3$/Hexane, respectively (as analyzed by ^{1}H NMR). The resulting two enantiomers were separated using a chiral pack AD-H column (10% IPA in hexanes; 1.0 mL/min. Analytical data for **2c-D**: ^{1}H NMR (500 MHz, $CDCl_3$) δ 7.35-7.43 (m, 5H), 5.29 (br s, 1H), 4.11 (dd, J = 8.5 Hz, J = 1.0 Hz, 1H), 3.83 (s, 1H), 3.79 (d, J = 8.0 Hz, 1H); ^{13}C NMR (125 MHz, $CDCl_3$) δ 157.8, 139.8, 128.93, 128.90, 124.7,

83.7, 50.4 (t, J = 22.0 Hz), 49.3. HRMS (ESI) Calculated Mass for $C_{10}H_9DNO_2Cl$: ($[M+H]^+$) = 213.0541, Found ($[M+H]^+$) = 213.0541.

XII. Procedure for the catalytic asymmetric chlorocyclization of labeled unsaturated carbamate 1c-D:

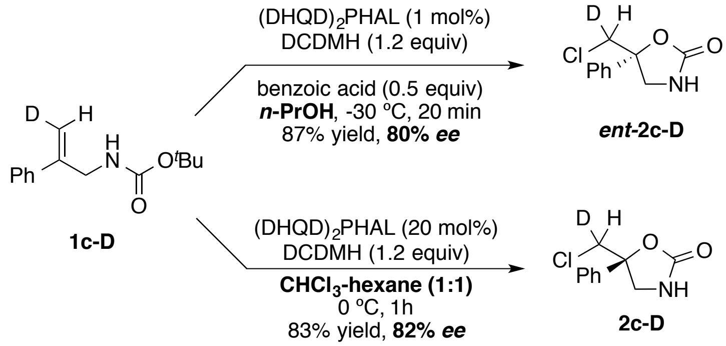

Procedure for the synthesis of carbamates **2c-D** and *ent*-**2c-D** is identical to the one reported for the non-labeled **2c** and *ent*-**2c** (Section XI).

XIII. (DHQD)$_2$PHAL catalyzed chlorocyclization of 1c-D monitored by ^{1}H NMR and HPLC:

To investigate the face selectivity of the chlorenium delivery, the labeled substrate **1c-D** was cyclized under both optimized catalytic conditions (i.e. cyclization in *n*-PrOH and in $CHCl_3$/Hexane) (see manuscript for details). Analysis of product ratios was accomplished using chiral HPLC and ^{1}H NMR spectroscopy (after the correction for considering the *E*:*Z* ratios) similar to that explained for amide substrate **1b-D** and previously reported alkenoic acid **1a-D**.[2b]

XIV. Absolute stereochemical assignment at the deuterated center of substrate 2c-D and *ent*-2c-D:

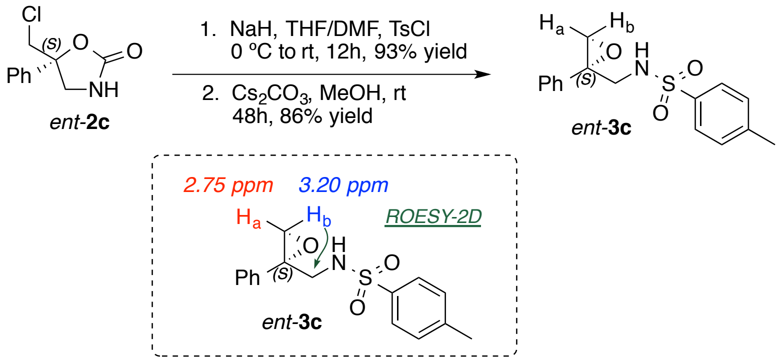

Tosyl protection of oxazolidinone *ent*-**2c** followed by cesium carbonate mediated ring opening of the resulting chlorohydrin intermediate returned 1,1-disubstituted epoxy sulfonamide *ent*-**3c**. ROESY study on the corresponding epoxy amide *ent*-**3c** show that H_a (2.75 ppm) has a *syn* orientation with the phenyl group and H_b (3.20 ppm) has an *anti* orientation with the phenyl group. After assignment of H_a and H_b, the labeled substrate *ent*-**2c-D** as a mixture (synthesized in the presence of the $(DHQD)_2PHAL$) was protected with tosyl chloride and sodium hydride, followed by cesium carbonate treatment to yield the desired labeled epoxy sulfonamide *ent*-**3c-D**. ^{1}H NMR analysis of the epoxy sulfonamide *ent*-**3c-D** shows only a peak at 2.75 ppm, which suggests that the deuterium has an *anti* orientation with respect to the phenyl group. Based on this finding, the deuterated carbon is assigned the *R* configuration for the corresponding epoxy sulfonamide *ent*-**3c-D** (see Figure S2b). Since the epoxide is formed through the S_N2 ring opening of the corresponding chlorohydrin intermediate, substrate *ent*-**3c-D** (major product) is assigned as the *S* configuration on the labeled C6 center. Similar procedure was applied to substrate **2c-D** to afford epoxide **3c-D** (Figure S2c).

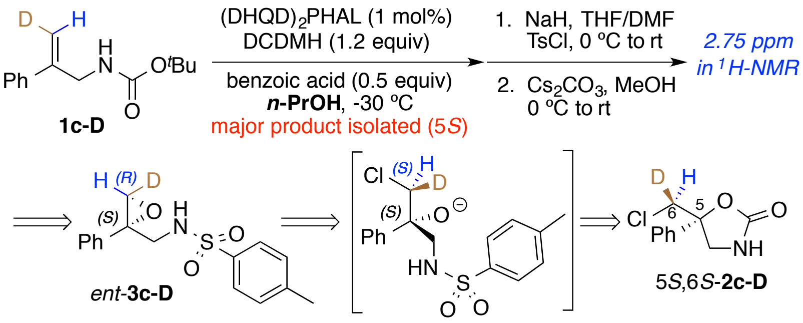

XV. General procedure for synthesis of labeled epoxy sulfonamides *ent*-3c and *ent*-3c-D:

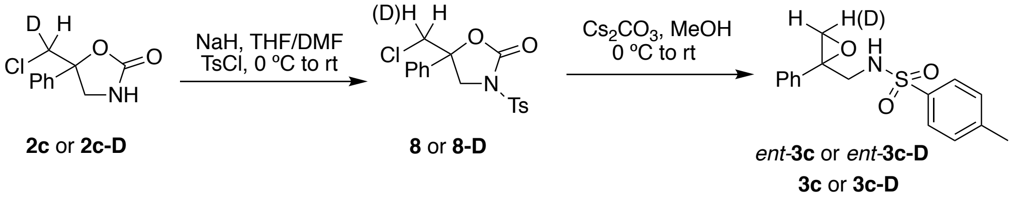

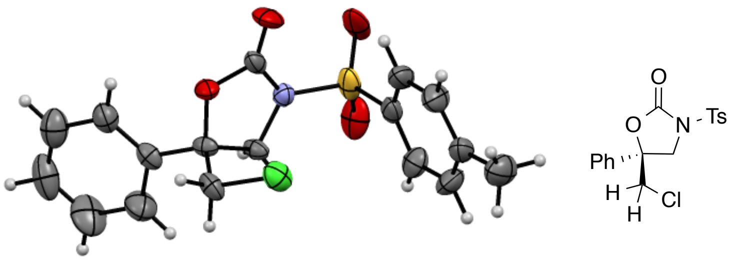

Figure S1. The X-ray structure of 5*S*-**8**.[4]

To a stirred suspension of NaH (60% wt., 12 mg, 0.28 mmol, 2.0 equiv) in a mixture of THF (0.8 mL) and DMF (0.5 mL) at 0 °C were added successively substrate *ent*-**2c-D** or *ent*-**3c** (30 mg, 0.14 mmol, 1.0 equiv) in THF (1.0 mL) and *p*-TsCl (35.2 mg, 0.18 mmol, 1.3 equiv). Stirring was continued for 18 h at room temperature, at which time the reaction was quenched by the addition of a saturated solution of NH_4Cl (2.0 mL) at -78 °C with vigorous stirring. The mixture was extracted with Et_2O, and the extract was washed successively with HCl (3 mL), $NaHCO_3$ (3 mL), H_2O (3 mL). The

combined organics were dried over $MgSO_4$ and the solvent was removed under reduced pressure. Purification by column chromatography on silica gel afforded the N-tosyl oxazolidinone (*5-(chloromethyl)-5-phenyl-3-tosyloxazolidin-2-one)* **8** in 93% yield. Analytical data for **8**: ^{1}H NMR (500 MHz, $CDCl_3$) δ 7.90 (d, *J* = 8.0 Hz, 2H), 7.34-7.41 (m, 3H), 7.29-7.33 (m, 4H), 4.52 (d, *J* = 9.5 Hz, 1H), 4.22 (d, *J* = 9.5 Hz, 1H), 3.72 (dd, *J* = 17.5 Hz, *J* = 12.5 Hz, 2H), 2.42 (s, 3H); ^{13}C NMR (125 MHz, $CDCl_3$) δ 150.3, 145.8, 138.1, 133.9, 129.8, 129.4, 129.1, 128.2, 124.4, 81.3, 52.8, 50.6, 21.7. HRMS (ESI) Calculated Mass for $C_{17}H_{16}NO_4SCl$: ($[M+H]^+$) = 366.0567, Found ($[M+H]^+$) = 366.0559. Absolute configuration of 5*S*-**8** (CCDC 1416555 is the corresponding Cambridge Structural Database deposition number) was determined by single crystal X-ray diffraction (XRD).[4]

Two diastereomers could be obtained from the deuterated substrate depending on the reaction condition. Analytical data for deuterated 5R,6S-**8-D** (diastereomer B obtained from chlorocyclization in $CHCl_3$-Hex): ^{1}H NMR (500 MHz, $CDCl_3$) δ 7.91 (d, *J* = 8.5 Hz, 2H), 7.36-7.41 (m, 3H), 7.29-7.33 (m, 4H), 4.51 (d, *J* = 9.5 Hz, 1H), 4.22 (d, *J* = 9.0 Hz, 1H), 3.73 (s, 1H), 2.43 (s, 3H). HRMS (ESI) Calculated Mass for $C_{17}H_{15}DClNO_4S$: ($[M+H]^+$) = 367.0630, Found ($[M+H]^+$) = 367.0626.

Analytical data for deuterated 5S,6S-**8-D** (diastereomer A obtained from chlorocyclization in *n*-PrOH): ^{1}H NMR (500 MHz, $CDCl_3$) δ 7.90 (d, *J* = 8.5 Hz, 2H), 7.35-7.41 (m, 3H), 7.29-7.33 (m, 4H), 4.51 (d, *J* = 9.0 Hz, 1H), 4.21 (d, *J* = 9.5 Hz, 1H), 3.69 (s, 1H), 2.42 (s, 3H); ^{13}C NMR (125 MHz, $CDCl_3$) δ 150.3, 145.9, 138.1, 134.0, 129.9, 129.4, 129.2, 128.3, 124.5, 81.2, 52.8, 50.4 (t, *J* = 23.0 Hz), 21.7. HRMS (ESI)

Calculated Mass for $C_{17}H_{15}DClNO_4S$: ($[M+H]^+$) = 367.0630, Found ($[M+H]^+$) = 367.0626.

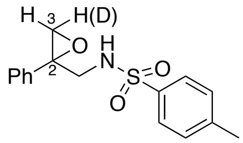

A catalytic amount of cesium carbonate (2 mg, 0.03 g.mmol^{-1}) was added to a solution of N-tosyl oxazolidinone **8** or **8-D** (20 mg, 0.55 mmol) in methanol (1mL). The reaction was stirred at room temperature for 12 h and was then concentrated in vacuum. The residue was purified by chromatography on a silica-gel column using a mixture of hexanes and ethyl acetate as the eluent to give the product in 86% yield (87% conversion). Analytical data for *ent*-**3c**: ^{1}H NMR (500 MHz, $CDCl_3$) δ 7.68 (d, *J* = 8.5 Hz, 2H), 7.25-7.33 (m, 7H), 4.51 (t, *J* = 4.0 Hz, 1H), 3.58 (dd, *J* = 8.5 Hz, *J* = 13.5 Hz, 1H), 3.46 (dd, *J* = 4.5 Hz, *J* = 13.5 Hz, 1H), 3.20 (d, *J* = 5.0 Hz, 1H), 2.74 (d, *J* = 5.0 Hz, 1H), 2.40 (s, 3H); ^{13}C NMR (125 MHz, $CDCl_3$) δ 143.7, 137.0, 136.6, 129.8, 128.6, 128.3, 127.0, 125.7, 58.6, 53.0, 45.6, 21.5. HRMS (ESI) Calculated Mass for $C_{16}H_{17}NO_3S$: ($[M+H]^+$) = 304.1007, Found ($[M+H]^+$) = 304.1003.

Analytical data for 2S,3R-*ent*-**3c-D** (diastereomer A obtained from chlorocyclization in *n*-PrOH): ^{1}H NMR (500 MHz, $CDCl_3$) δ 7.68 (d, *J* = 8.0 Hz, 2H), 7.25-7.33 (m, 7H), 4.51 (dd, *J* = 4.5 Hz, *J* = 8.0 Hz, 1H), 3.59 (dd, *J* = 8.5 Hz, *J* = 13.5 Hz, 1H), 3.46 (dd, *J* = 4.0 Hz, *J* = 13.5 Hz, 1H), 2.74 (s, 1H), 2.40 (s, 3H); ^{13}C NMR (125 MHz, $CDCl_3$) δ 143.6, 137.0, 136.5, 129.7, 128.6, 128.3, 127.0, 125.7, 58.5, 52.7 (t, *J* = 26.8 Hz), 45.6, 21.5. HRMS (ESI) Calculated Mass for $C_{16}H_{16}DNO_3S$: ($[M+Na]^+$) = 327.0890, Found ($[M+Na]^+$) = 327.0886.

Analytical data for 2R,3R-*ent*-**3c-D** (diastereomer B obtained from chlorocyclization in $CHCl_3$:Hex): ^{1}H NMR (500 MHz, $CDCl_3$) δ 7.69 (d, J = 8.5 Hz, 2H), 7.25-7.32 (m, 7H), 4.51 (t, J = 4.0 Hz, 1H), 3.59 (dd, J = 8.0 Hz, J = 13.5 Hz, 1H), 3.46 (dd, J = 4.5 Hz, J = 13.5 Hz, 1H), 3.19 (s, 1H), 2.40 (s, 3H); ^{13}C NMR (125 MHz, $CDCl_3$) δ 143.6, 137.0, 136.6, 129.8, 128.6, 128.3, 127.0, 125.7, 58.5, 52.7 (t, J = 28.0 Hz), 45.7, 21.5. HRMS (ESI) Calculated Mass for $C_{16}H_{16}DNO_3S$: ($[M+Na]^+$) = 327.0890, Found ($[M+Na]^+$) = 327.0886.

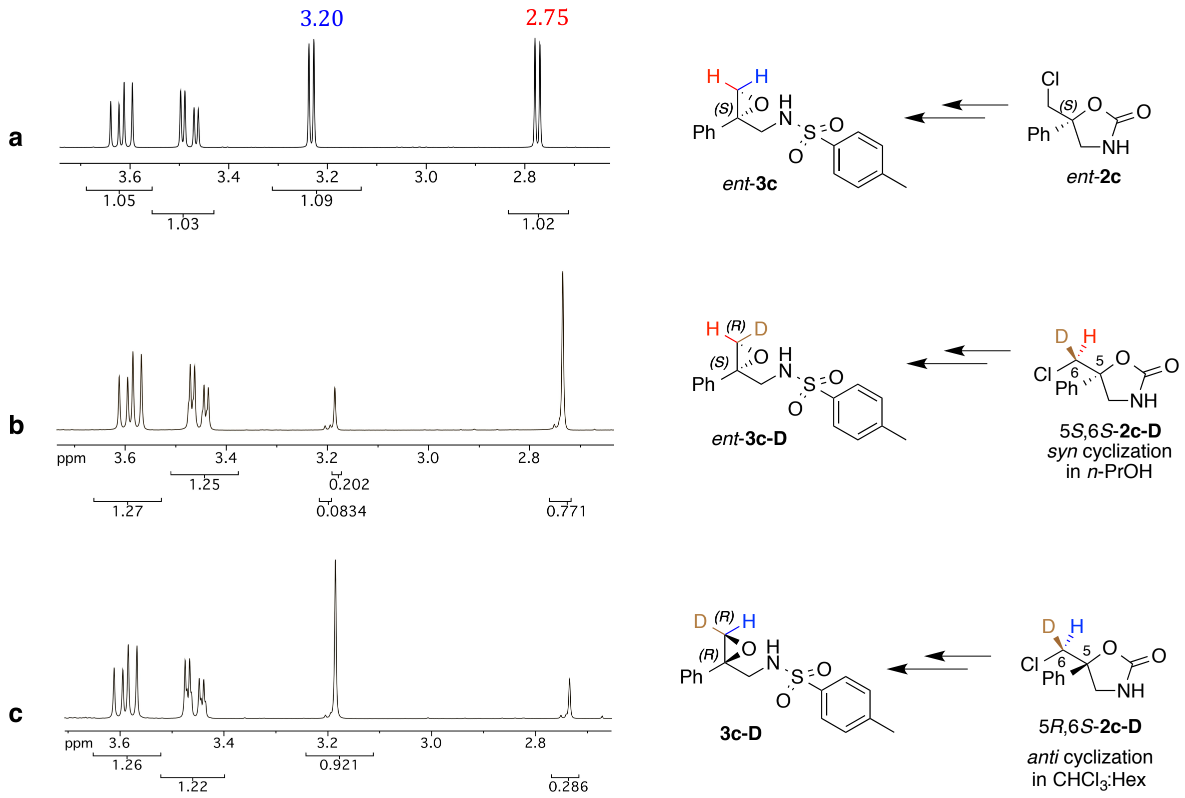

Figure S2. Summary of stereochemical assignments coupled with the NMR spectra of the epoxides.

XVI. *E/Z* ratios of recovered labeled starting materials

To ensure that various *syn*:*anti* addition ratios seen as a function of starting material and reaction conditions are independent of *cis*-*trans* isomerization of the starting olefin during the course of the reaction (for example, reversibility of a putative cationic species that could stereo-randomize the starting olefin, see Scheme 3 in the main text) a detailed analysis of the starting material geometry at various reaction conversions for each reaction was performed. The data in Table S2 clearly shows that the stereochemical integrity of the starting material for labeled substrates **1b-D** and **1c-D** recovered during the course of *Reactions B*, *C*, and *C'* do not change.

Table S3. *E*/*Z* ratios of recovered labeled starting materials at various conversions.

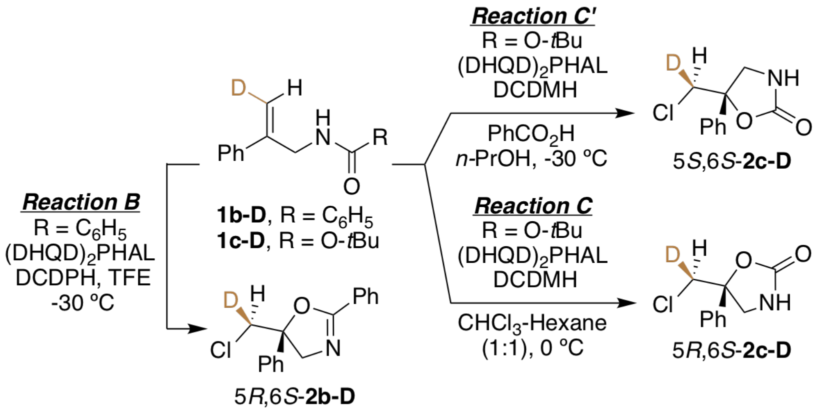

Entry	Substrate, *Reaction*[a]	Conv.[b]	*E*/*Z* ratio (**1b-D** or **1c-D**)[c]
1	**1b-D**, *B*	0%	90 : 10
2	**1b-D**, *B*	28%	90 : 10
3	**1b-D**, *B*	30%	90 : 10
4	**1b-D**, *B*	31%	90 : 10
5	**1b-D**, *B*	49%	90 : 10
6	**1b-D**, *B*	59%	89 : 11
7	**1b-D**, *B*	76%	90 : 10
8	**1c-D**, *C*	0%	92 : 8
9	**1c-D**, *C*	42%	90 : 10
10	**1c-D**, *C*	56%	89 : 11
11	**1c-D**, *C*	68%	90 : 10
12	**1c-D**, *C*	80%	91 : 9
13	**1c-D**, *C'*	0%	92 : 8
14	**1c-D**, *C'*	8%	92 : 8
15	**1c-D**, *C'*	17%	92 : 8
16	**1c-D**, *C'*	26%	91 : 9
17	**1c-D**, *C'*	60%	91 : 9

[a]1.1 equiv chlorine source was added; 2, 20, and 1 mol% catalyst loadings were used in reactions **B**, **C**, and **C'**, respectively; [b]Conversion% of **2b-D** was measured by ^{1}H NMR analysis of the crude reaction; [c]*E*/*Z* isomeric ratios of the recovered starting material were measured by ^{1}H NMR analysis.

XVII. Summaries of computational modeling: (a) Uncatalyzed reaction:

Species	EDF2/6-31G* Energy (au)	Erel vs Separate Starting Components (kcal/mol)	EDF2/6-31G* ΔH0 (au)	ΔHrel vs Separate Starting Components (kcal/mol)	EDF2/6-31G* ΔG0 (au)	ΔGrel vs Separate Starting Components (kcal/mol)	T1 ΔH0 values (kcal/mol)	Gas phase ΔΔH values (kcal/mol)	ΔG = T1+EDF2(ΔH->ΔG) (kcal/mol)	Gas phase ΔΔG values	SMD CHCl3 Esolv (kcal/mol)	SMD 1-propanol Esolv (kcal/mol)	SMD TFE Esolv (kcal/mol)	ΔG values from T1 ΔH0 + EDF2/6-31G* (ΔH->ΔG) + SMD CHCl3 solvation ΔG	ΔΔG values in SMD CHCl3	ΔG values from T1 ΔH0 + EDF2/6-31G* (ΔH->ΔG) + SMD 1-propanol solvation ΔG	ΔΔG values in SMD 1-propanol	ΔG values from T1 ΔH0 + EDF2/6-31G* (ΔH->ΔG) + SMD TFE solvation ΔG	ΔΔG values in SMD TFE
DCDMH	-1374.20682		-1374.08364		-1374.13423		-52.59		-84.34		-8.99	-9.38	-5.99	-93.33		-93.71		-90.33	
PhCONHCH2CPh=CH2 lowest conformer	-748.14542		-747.869228		-747.93135		12.89		-26.09		-14.41	-13.66	-11.89	-40.50		-39.75		-37.98	
Cyclized product	-1207.77136		-1207.50288		-1207.56576		5.37		-34.09		-14.90	-13.85	-12.77	-48.99		-47.94		-46.86	
Monochlorohydantoin-NH	-914.63369		-914.501442		-914.54841		-76.85		-106.32		-9.28	-10.75	-7.60	-115.60		-117.07		-113.92	
Sum DCDMH + 1 alkene substrate **1b**	-2122.35224	[0]	-2121.952868	[0]	-2122.06558	[0]	-39.70	[0]	-110.43	[0]	-23.40	-23.03	-17.88	-133.83	[0]	-133.46	[0]	-128.30	[0]
Unimolecular (in alkene **1b**) initial comple	-2122.36467	-7.80	-2121.96486	-7.52	-2122.05854	4.42	-52.74	-13.04	-111.53	-1.10	-19.38	-18.84	-14.59	-130.90	2.92	-130.37	3.10	-126.11	2.19
bimolecular TS (shown in Fig. 4b in text)	-2122.31481	23.49	-2121.91654	22.80	-2122.00726	36.60	-11.53	28.17	-68.46	41.97	-22.91	-24.84	-21.36	-91.36	42.46	-93.29	40.17	-89.82	38.48
bimolecular cyclized product complex	-2122.40153	-30.93	-2122.00206	-30.87	-2122.09544	-18.74	-76.35	-36.65	-134.95	-24.52	-21.04	-20.60	-16.96	-155.98	-22.16	-155.54	-22.08	-151.91	-23.61
Separated products	-2122.40505	-33.14	-2122.00432	-32.29	-2122.11417	-30.49	-71.48	-31.78	-140.41	-29.98	-24.18	-24.60	-20.37	-164.59	-30.76	-165.01	-31.54	-160.77	-32.47
Sum DCDMH + 2 alkene substrates **1b**	-2870.49765	[0]	-2869.822096	[0]	-2869.99693	[0]	-26.81	[0]	-136.52	[0]	-37.80	-36.69	-29.76	-174.33	[0]	-173.21	[0]	-166.28	[0]
Bimolecular (in alkene **1b**) initial complex	-2870.51929	-13.58	-2869.84342	-13.38	-2869.98274	8.91	-48.54	-21.73	-135.96	0.56	-32.85	-31.39	-25.91	-168.82	5.51	-167.35	5.86	-161.88	4.40
termolecular lowest TS (shown in Fig. 4a)	-2870.50296	-3.33	-2869.82703	-3.10	-2869.96125	22.39	-29.55	-2.74	-113.77	22.75	-33.27	-33.06	-28.49	-147.04	27.28	-146.83	26.38	-142.26	24.02
termolecular cyclized product	-2870.55744	-37.52	-2869.87988	-36.26	-2870.01285	-9.99	-72.17	-45.36	-155.61	-19.09	-34.14	-33.60	-29.21	-189.75	-15.42	-189.21	-16.00	-184.82	-18.54
Separated products	-2870.55047	-33.14	-2869.87355	-32.29	-2870.04552	-30.49	-58.59	-31.78	-166.50	-29.98	-38.59	-38.26	-32.25	-205.09	-30.76	-204.76	-31.54	-198.75	-32.47

Uncatalyzed chlorocyclization reaction summary. This spreadsheet summarizes the uncatalyzed chlorocyclization energetics as computed at the EDF2/6-31G* and T1 levels of theory. Energies for each structure are listed in atomic units (hartrees; 1 H = 627.51 kcal/mol), while derived energy differences are shown in kcal/mol. DCDMH = Dichlorodimethylhydantoin, the chlorenium delivery reagent. TFE = 2,2,2-trifluoroethanol. Alkene substrate ($PhCONHCH_2CPh{=}CH_2$) energies represent the most stable conformer as determined at the EDF2/6-31G* level. Monochlorohydantoin byproduct is the lowest energy tautomer, with H on the N from which Cl had been abstracted. "Unimolecular" and "bimolecular"label refer to the number of substrate alkene molecules involved in the scheme, not total molecularity.

XVIII. Summaries of computational modeling (b) $(DHQD)_2$Phal-catalyzed reaction:

Species optimized at PM6 level	**gas phase PM6 ΔH0 geometry optimized (kcal/mol)**	gas phase energy: B3LYP-D3/6-31G*//PM6 (au)	**gas phase energy B3LYP-D3/6-31G*//PM6 (kcal/mol)**	PM6 Corrns (vib, trans, rot) to ΔH (kcal/mol)	**ΔH0 (kcal/mol)**	PM6 (ΔH->ΔG correction) at 298K (kcal/mol)	**ΔG0 (kcal/mol)**	B3LYP-D3/6-31G*//PM6 CPCM solvation ΔG (1-propanol; dielectric=20.52) (Spartan implementation, kcal/mol)	ΔG0 in 1-propanol (kcal/mol)	B3LYP-D3/6-31G*//PM6 CPCM solvation ΔG (CHCl3) dielectric=4.71) (Spartan implementation, kcal/mol)	ΔG0 in CHCl3 (kcal/mol)
DHQD2PHAL alone	-22.51	-2491.113121	-1563198.4	581.6	-1562616.8	494.9	-1562703.4	-18.9	-1562722.3	-15.2	-1562718.7
carbamate **1c** alone (lowest conformer)	-80.32	-750.173478	-470741.4	188.1	-470553.3	147.3	-470594.0	-7.1	-470601.1	-5.7	-470599.7
1carbamate **1c** in DHQD2PHAL	-109.61	-3241.321654	-2033961.8	769.2	-2033192.6	656.3	-2033305.5	-21.6	-2033327.1	-17.3	-2033322.8
2carbamates **1c** in DHQD2PHAL (Fig 9a in text)	-197.16	-3991.538562	-2504730.4	957.0	-2503773.3	816.0	-2503914.3	-24.9	-2503939.2	-20.0	-2503934.3
DCDMH alone	-45.69	-1374.451957	-862482.3	75.2	-862407.1	44.3	-862438.0	-7.3	-862445.3	-5.9	-862443.9
2carbamates **1c** in DHQD2PHAL+DCDMH pre-rxn complex (Fig 9b in text)	-251.58	-5366.010895	-3367225.5	1032.4	-3366193.0	873.7	-3366351.8	-28.7	-3366380.6	-23.0	-3366374.9
2carbamates **1c** in DHQD2PHAL+DCDMH anti TS (Fig 9c in text)	-231.15	-5365.942767	-3367182.7	1032.0	-3366150.7	875.2	-3366307.5	-33.6	-3366341.2	-26.9	-3366334.5
2carbamates **1c** in DHQD2PHAL+DCDMH syn TS (Fig 9d in text)	-235.60	-5365.949604	-3367187.0	1031.6	-3366155.4	875.6	-3366311.5	-29.1	-3366340.6	-23.4	-3366334.9
DHQD2PHAL + carbamate --> 1carbamate in DHQD2PHAL	-6.8		-22.0		-22.5		-8.0		-3.6		-4.3
1carbamate in DHQD2PHAL + carbamate --> 2carbamates in DHQD2PHAL	-7.2		-27.3		-27.5		-14.8		-11.0		-11.8
DHQD2PHAL + 2carbamate --> 2carbamate-DHQD2PHAL complex	-14.0		-49.3		-49.9		-22.9		-14.6		-16.2
2carbamate in DHQD2PHAL + DCDMH -->											
2carbamates in DHQD2PHAL + DCDMH pre-rxn complex	-8.7		-12.8		-12.6		0.5		3.9		3.3
2carbamate-DHQD2PHAL-DCDMH complex --> *syn* addition TS	16.0		38.5		37.6		40.4		40.0		40.0
2carbamate-DHQD2PHAL-DCDMH complex --> *anti* addition TS	20.4		42.8		42.3		44.3		39.4		40.4
2carbamate in DHQD2PHAL + DCDMH --> *syn* addition TS	7.3		25.7		25.0		40.9		43.9		43.3
2carbamate in DHQD2PHAL + DCDMH --> *anti* addition TS	11.7		30.0		29.7		44.8		43.3		43.7
DHQD2PHAL + 2carbamate + DCDMH --> *syn* addition TS	-6.8		-23.6		-24.9		18.0		29.3		27.2
DHQD2PHAL + 2carbamate + DCDMH --> *anti* addition TS	-2.3		-19.3		-20.2		21.9		28.7		27.6

$(DHQD)_2$PHAL-catalyzed chlorocyclization reaction summary. This spreadsheet summarizes the catalyzed chlorocyclization energetics as computed at the B3LYP-D3/6-31G*//PM6 level of theory with further explorations of solvation effects. All calculations were performed with the Spartan '16 quantum chemistry package. Energies for each structure are listed in kcal/mol (except for the initial total electronic energies, which are also listed in atomic units (hartrees; 1 H = 627.51 kcal/mol). All derived energy differences are shown in kcal/mol. DCDMH = Dichlorodimethylhydantoin, the chlorenium delivery reagent. TFE = 2,2,2-trifluoroethanol. Alkene substrate ($PhCONHCH_2CPh{=}CH_2$) energies represent the most stable conformer as determined at the EDF2/6-31G* level. Monochlorohydantoin byproduct is the lowest energy tautomer, with H on the N from which Cl had been abstracted. "Unimolecular" and "bimolecular"labels refer to the number of substrate alkene molecules involved in the scheme, not total molecularity.

XIX. References:

(1) Zeng, H.; Hua, R. *J. Org. Chem.* **2008**, *73*, 558.

(2) (a) Whitehead, D. C.; Yousefi, R.; Jaganathan, A.; Borhan, B. *J. Am. Chem. Soc.* **2010**, *132*, 3298; (b) Yousefi, R.; Ashtekar, K. D.; Whitehead, D. C.; Jackson, J. E.; Borhan, B. *J. Am. Chem. Soc.* **2013**, *135*, 14524.

(3) Jaganathan, A.; Garzan, A.; Whitehead, D. C.; Staples, R. J.; Borhan, B. *Angew. Chem. Int. Ed.* **2011**, *50*, 2593.

(4) Garzan, A.; Jaganathan, A.; Salehi Marzijarani, N.; Yousefi, R.; Whitehead, D. C.; Jackson, J. E.; Borhan, B. *Chem. Eur. J.* **2013**, *19*, 9015.

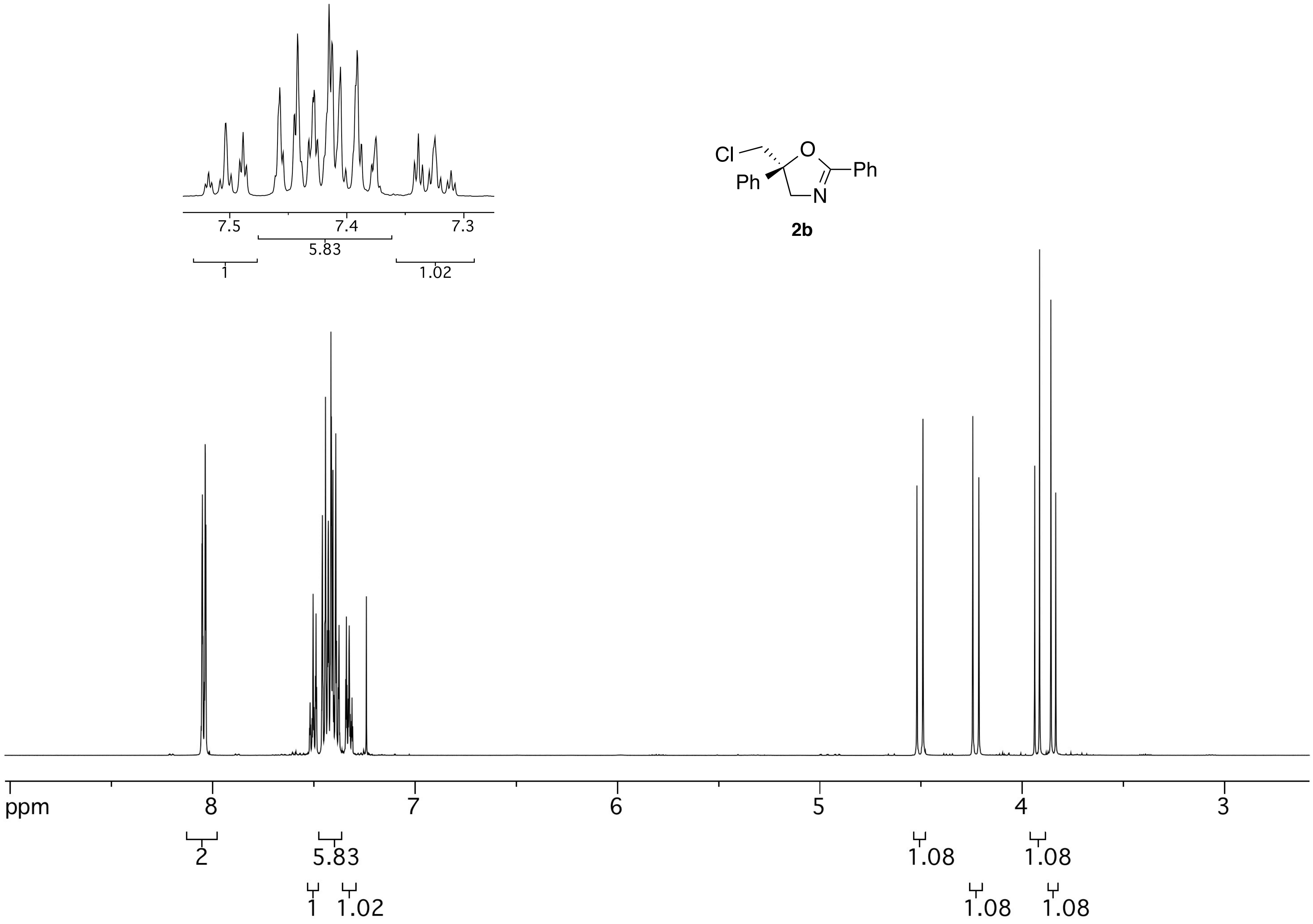

7.5
7.4
7.3
5.83
1
1.02
ppm
8
7
6
5
4
3
2
5.83
1.08
1.08
1
1.02
1.08
1.08
Cl
O
Ph
Ph
N

2b

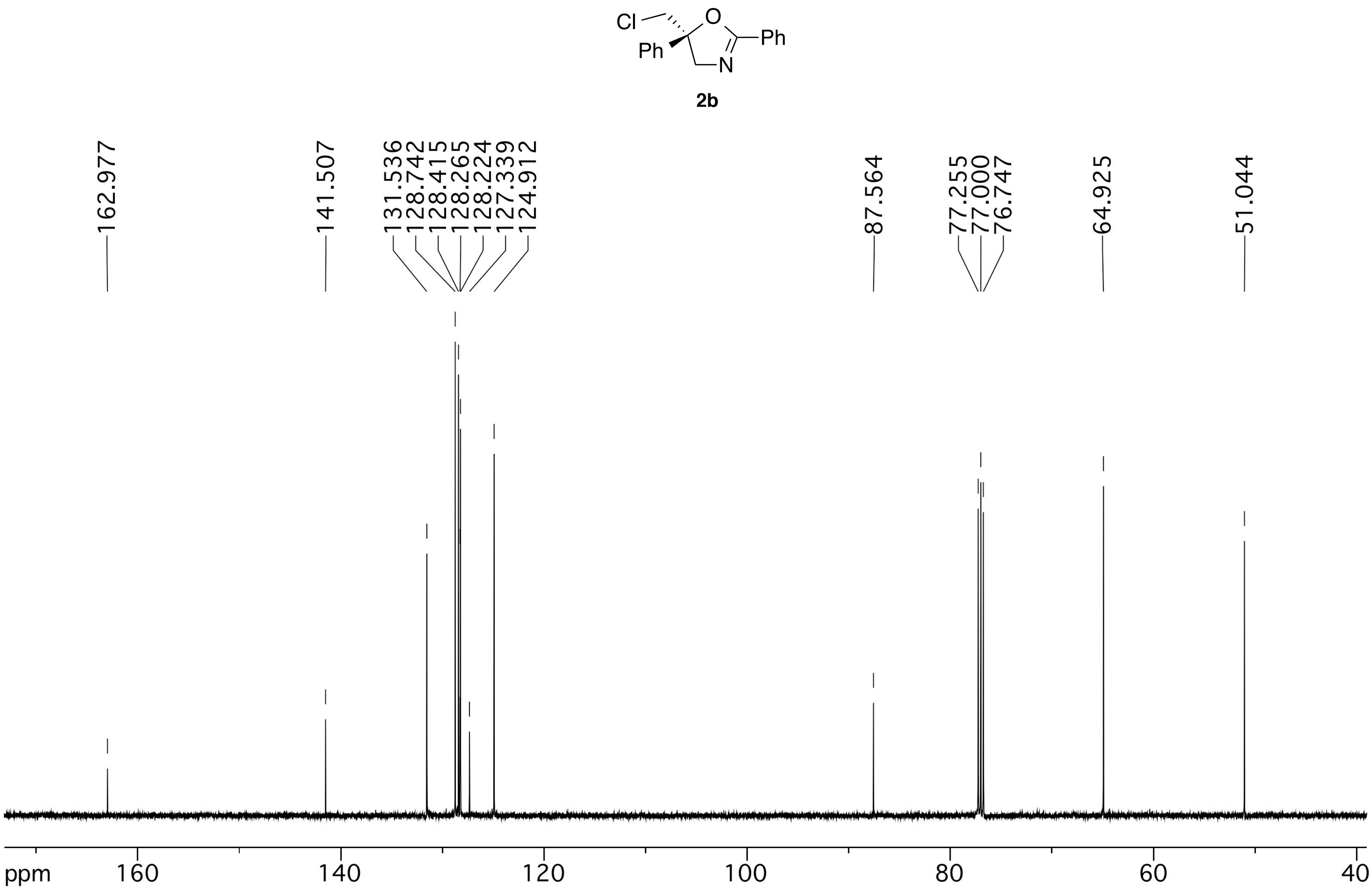
Cl
O
Ph
Ph
N
2b
162.977
141.507
131.536
128.742
128.415
128.265
128.224
127.339
124.912
87.564
77.255
77.000
76.747
64.925
51.044
ppm
160
140
120
100
80
60
40

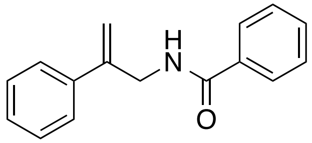

1b

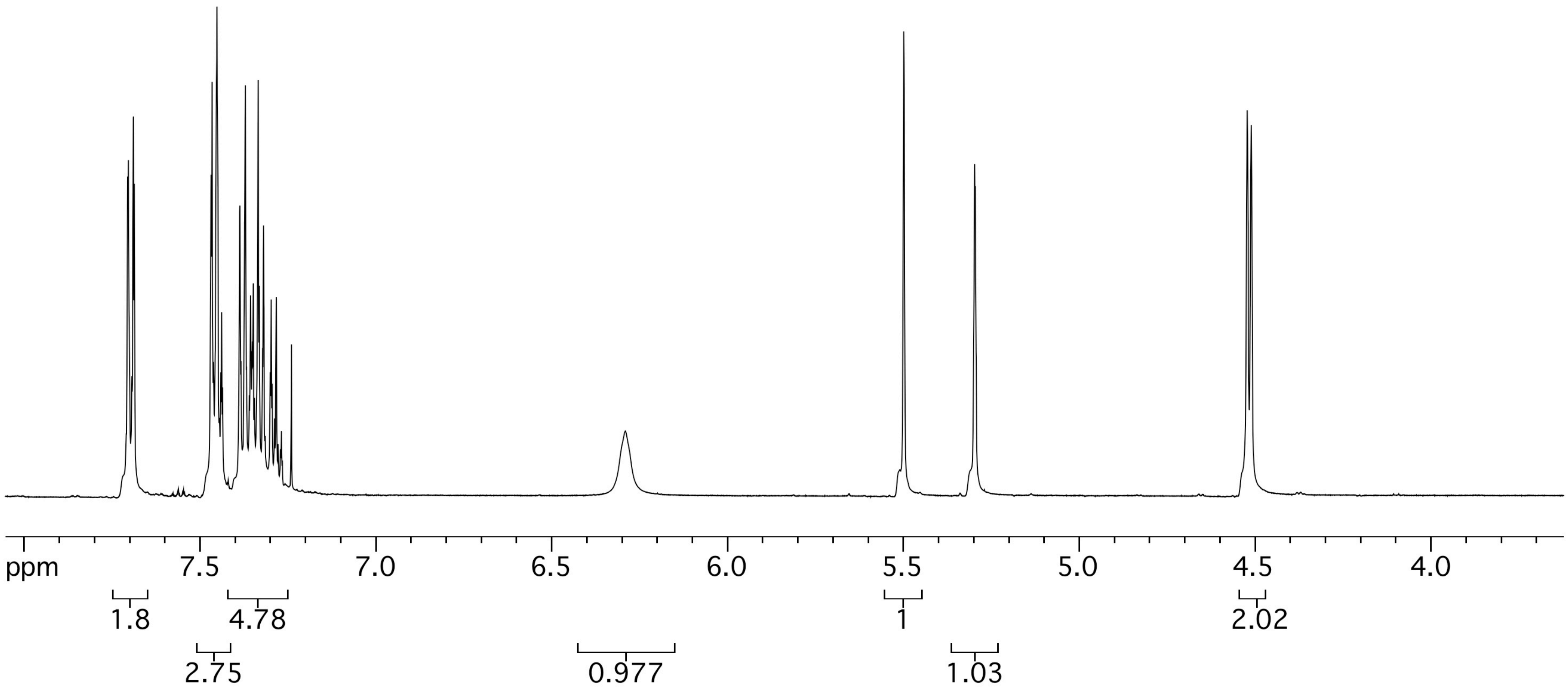

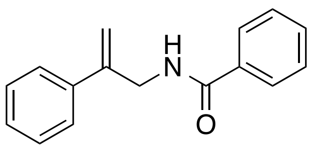
H
N
O

1b

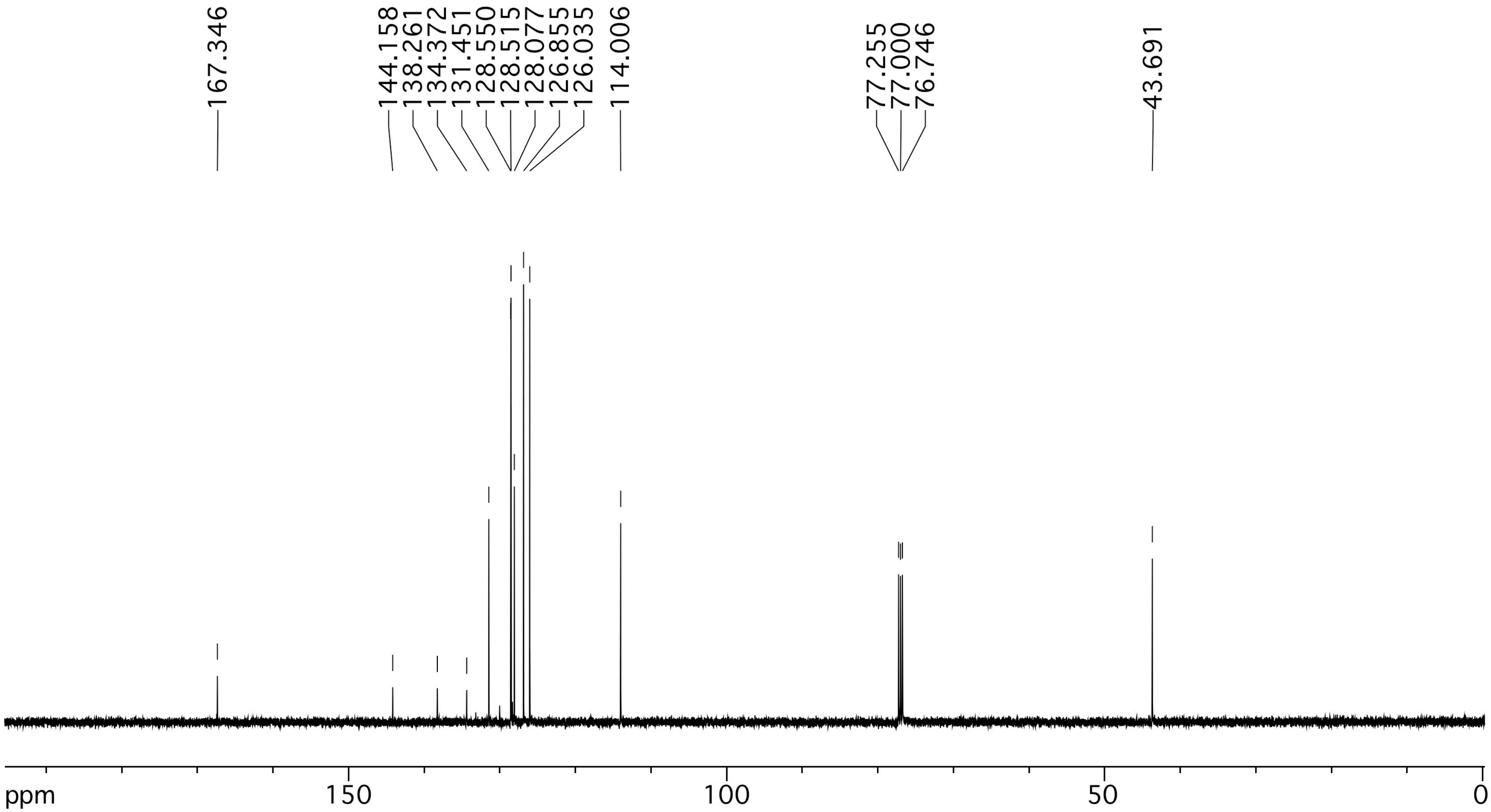
167.346
144.158
138.261
134.372
131.451
128.550
128.515
128.077
126.855
126.035
114.006
77.255
77.000
76.746
43.691
ppm
150
100
50
0

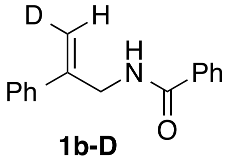
D
H
H
N
Ph
Ph
O

1b-D

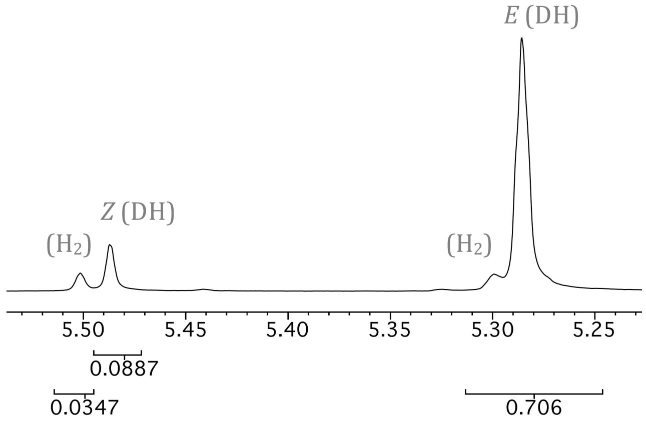
E (DH)
Z (DH)
(H2)
(H2)
5.50 5.45 5.40 5.35 5.30 5.25
0.0887
0.0347
0.706

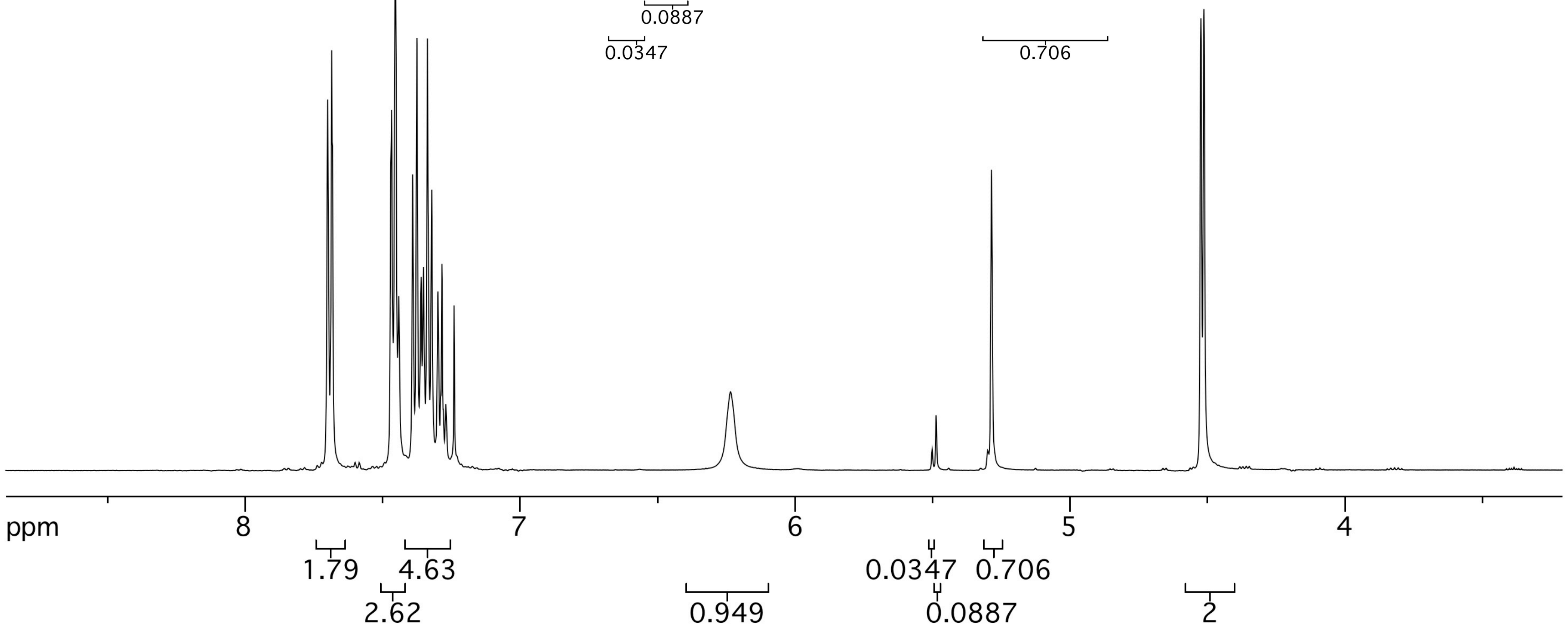
ppm
8 7 6 5 4
1.79 4.63
2.62
0.949
0.0347 0.706
0.0887
2

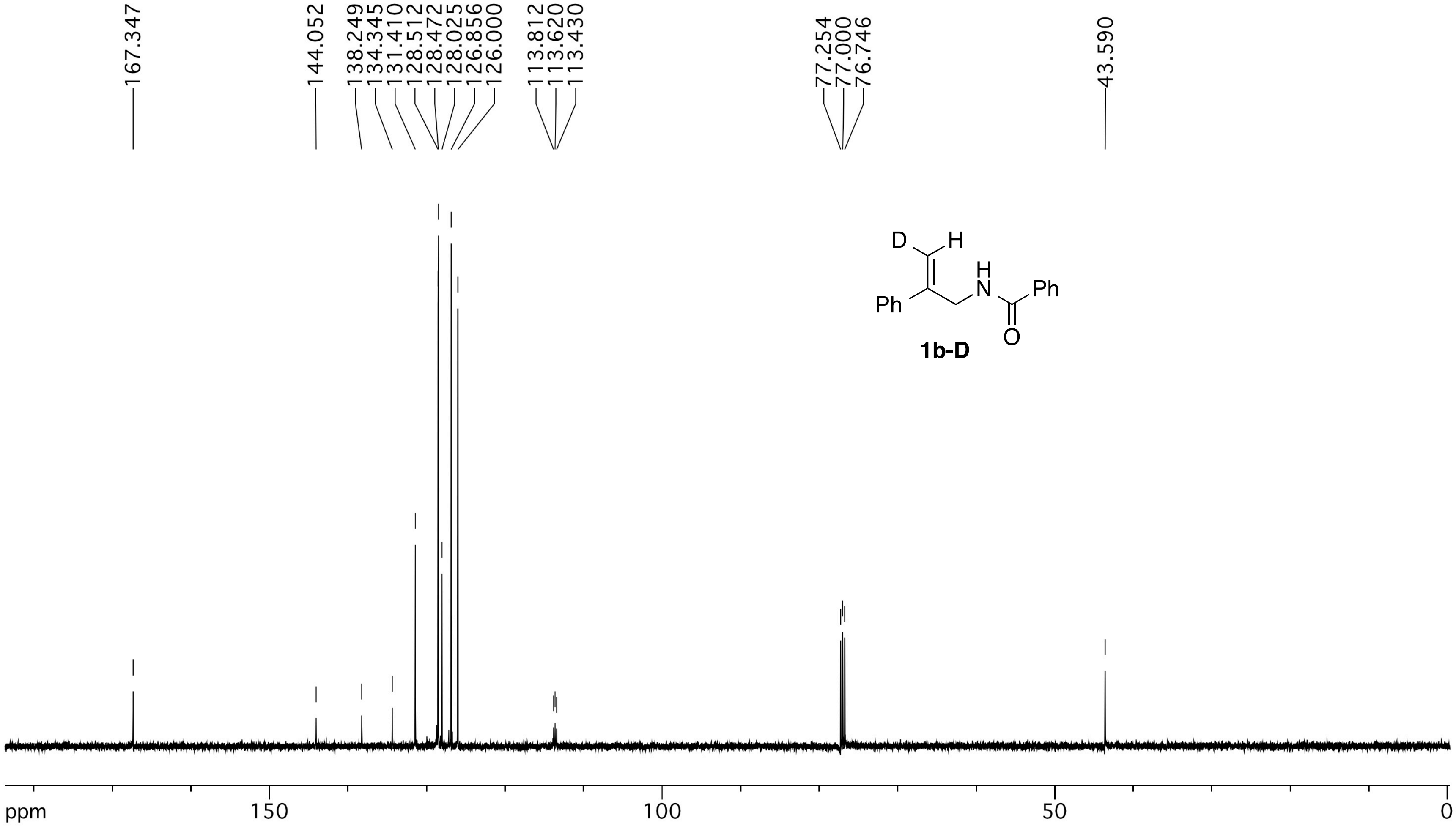

167.347
144.052
138.249
134.345
131.410
128.512
128.472
128.025
126.856
126.000
113.812
113.620
113.430
77.254
77.000
76.746
43.590
D
H
H
N
Ph
Ph
O
1b-D
ppm
150
100
50
0

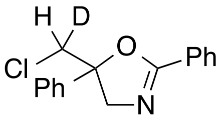

H
D
Cl
O
Ph
Ph
N

2b-D

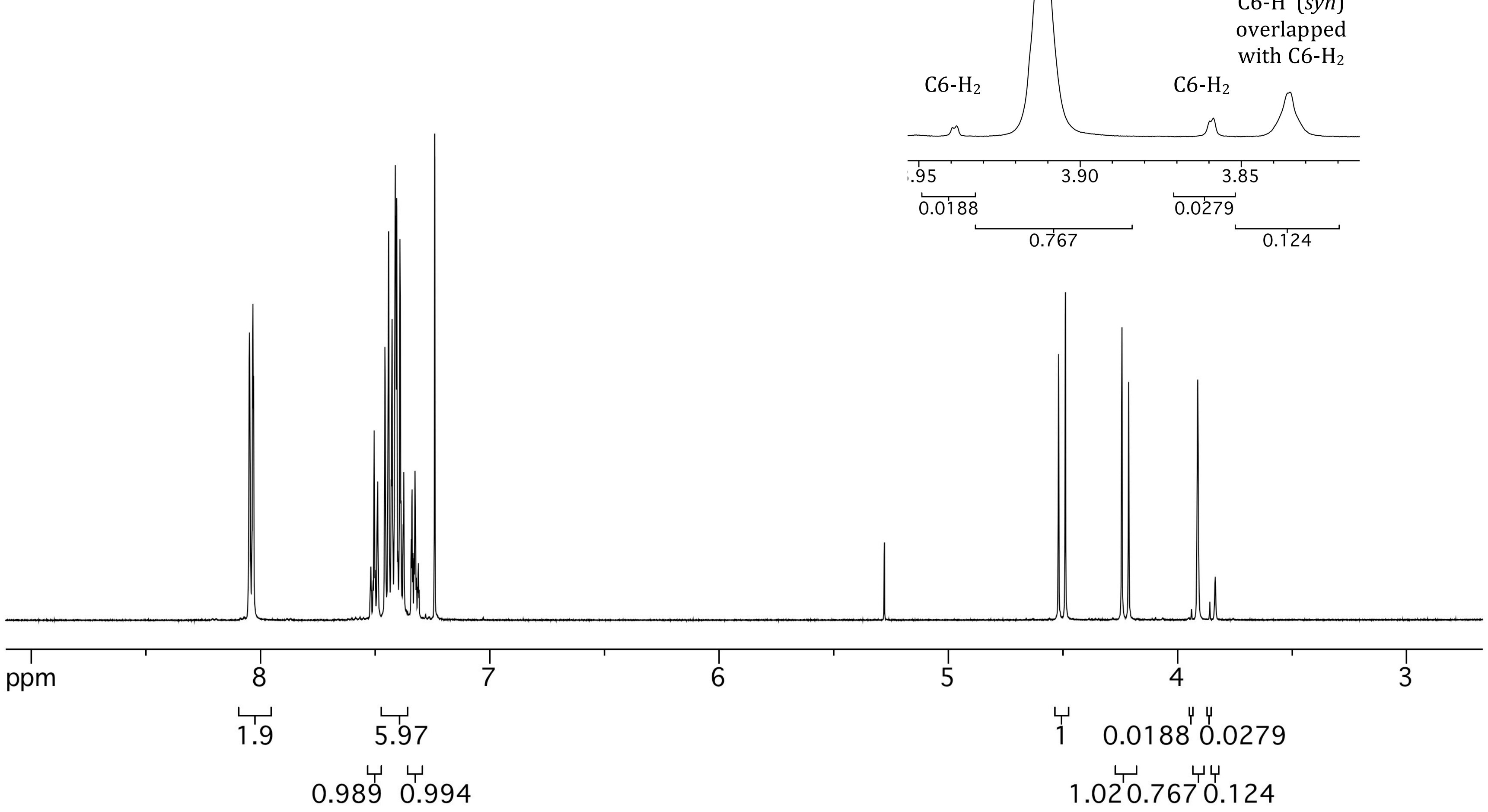

C6-H (*anti*) overlapped with C6-H2
C6-H2
C6-H2
C6-H (*syn*) overlapped with C6-H2
.95
3.90
3.85
0.0188
0.767
0.0279
0.124
ppm
8
7
6
5
4
3
1.9
5.97
1
0.0188
0.0279
0.989
0.994
1.02
0.767
0.124

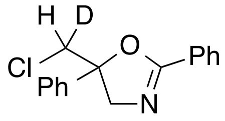
H
D
O
Ph
Cl
Ph
N

2b-D

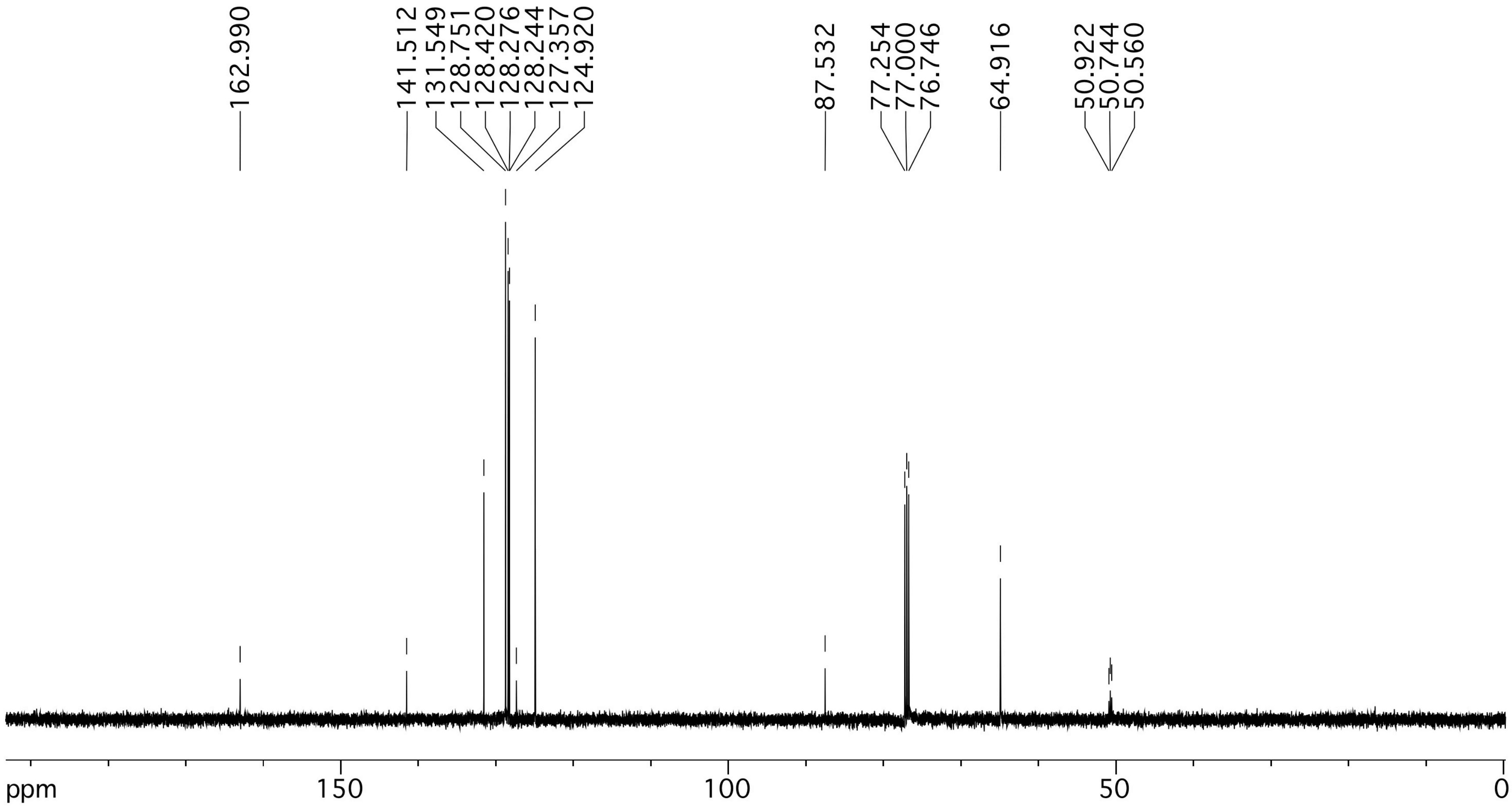
162.990
141.512
131.549
128.751
128.420
128.276
128.244
127.357
124.920
87.532
77.254
77.000
76.746
64.916
50.922
50.744
50.560
ppm
150
100
50
0

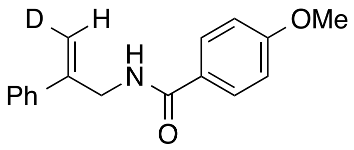
D
H
H
N
OMe
Ph
O

9b-D

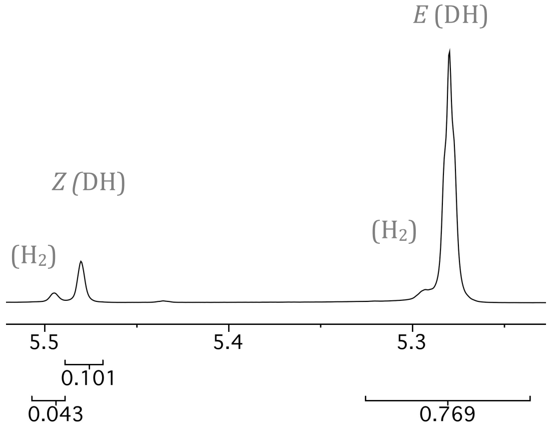
E (DH)
Z (DH)
(H2)
(H2)
5.5
5.4
5.3
0.101
0.043
0.769

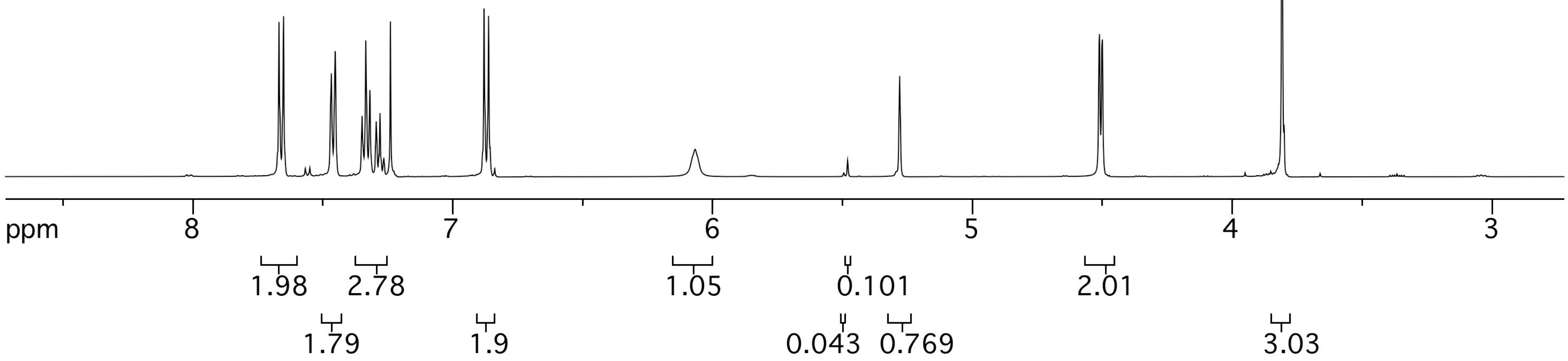
ppm
8
7
6
5
4
3
1.98
2.78
1.05
0.101
2.01
1.79
1.9
0.043
0.769
3.03

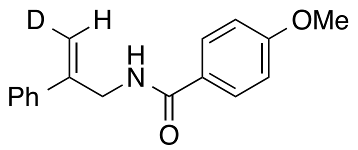
D
H
OMe
H
N
Ph
O

9b-D

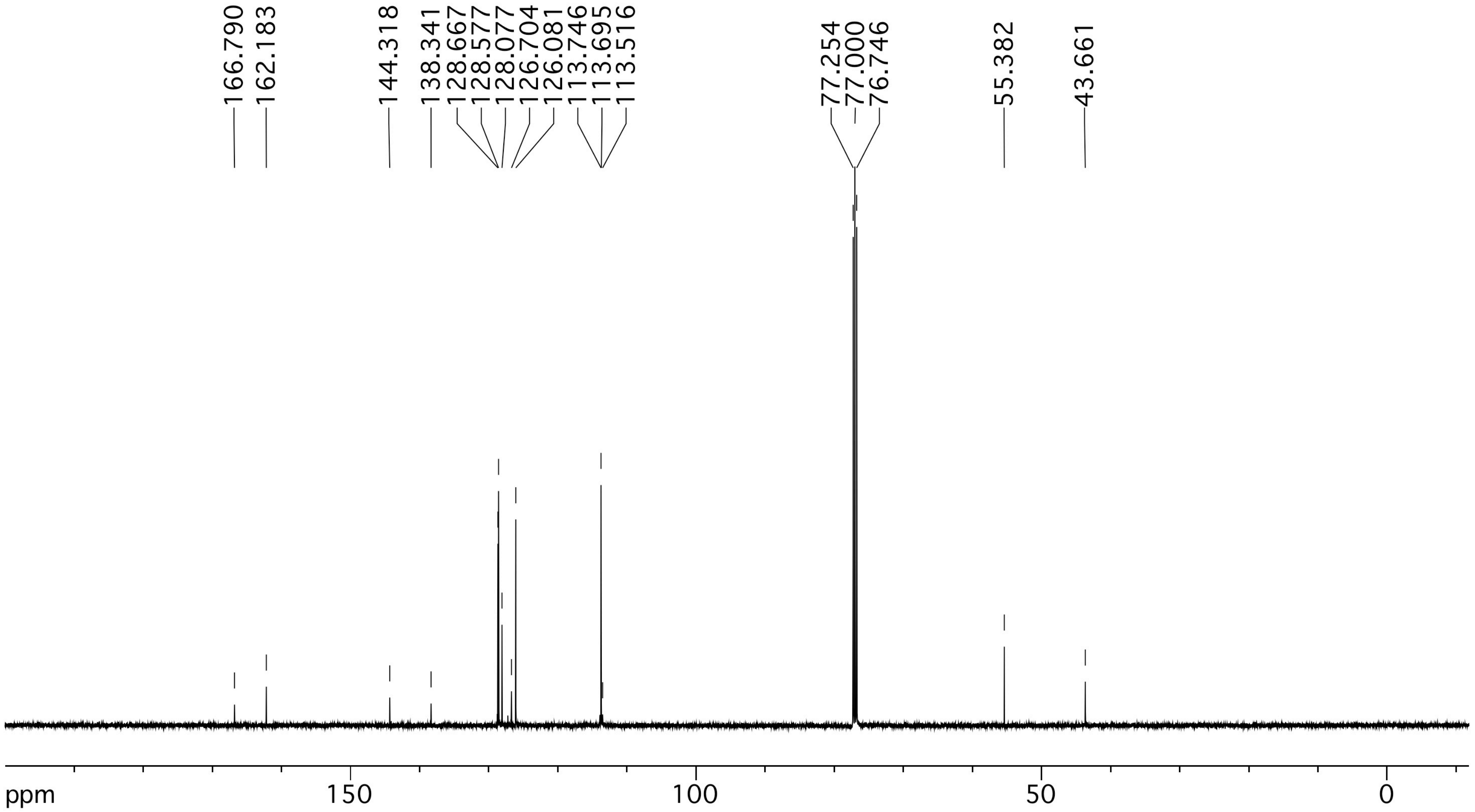
166.790
162.183
144.318
138.341
128.667
128.577
128.077
126.704
126.081
113.746
113.695
113.516
77.254
77.000
76.746
55.382
43.661
ppm
150
100
50
0

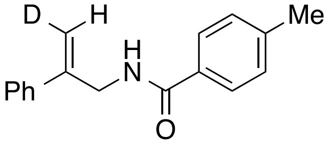
D
H
H
N
Me
Ph
O

10b-D

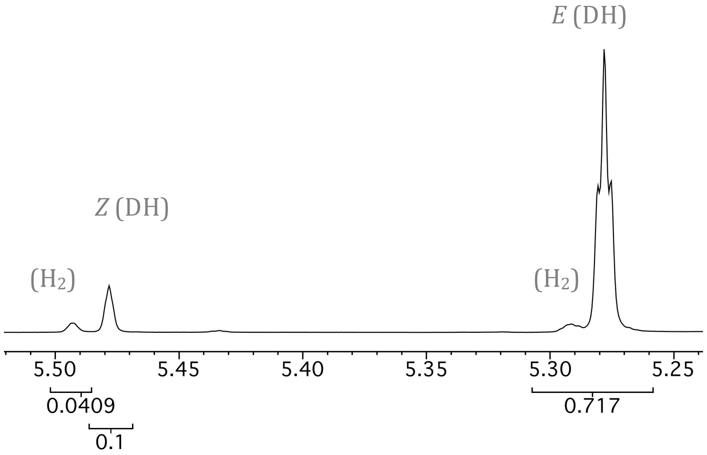
E (DH)
Z (DH)
(H2)
(H2)
5.50
5.45
5.40
5.35
5.30
5.25
0.0409
0.1
0.717

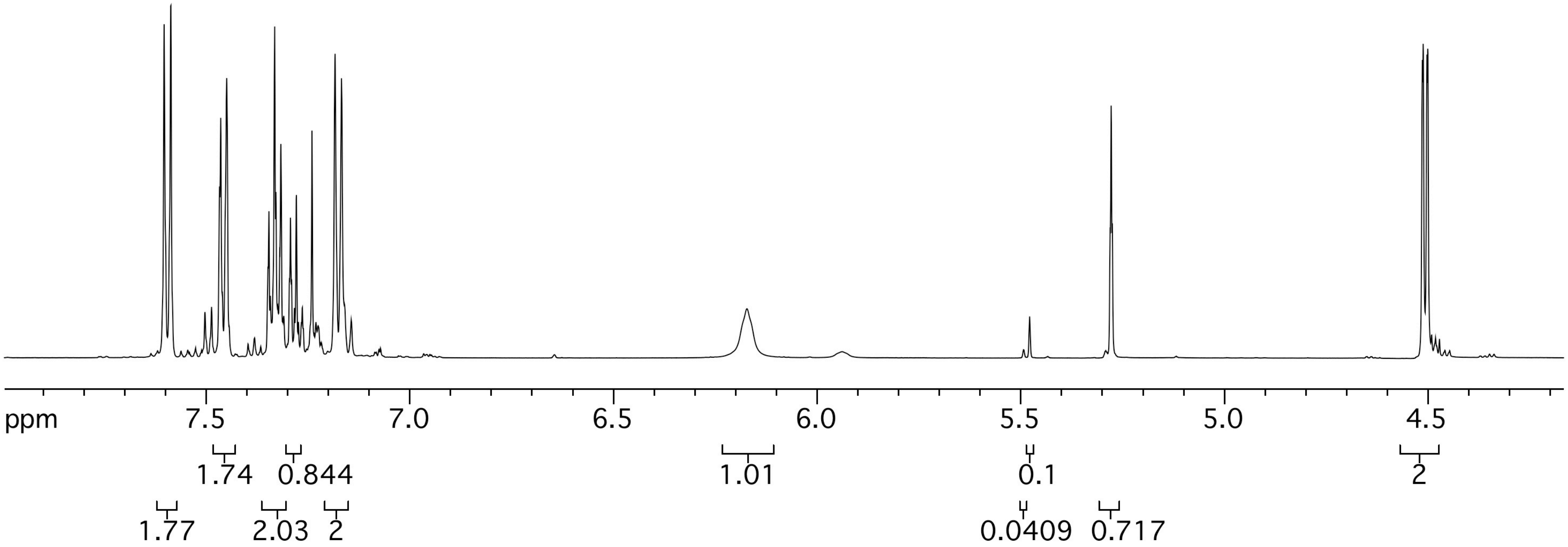
ppm
7.5
7.0
6.5
6.0
5.5
5.0
4.5
1.74
0.844
1.01
0.1
2
1.77
2.03
2
0.0409
0.717

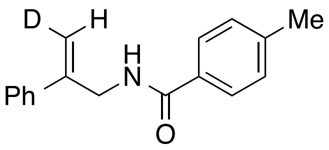
D
H
H
N
Me
Ph
O

10b-D

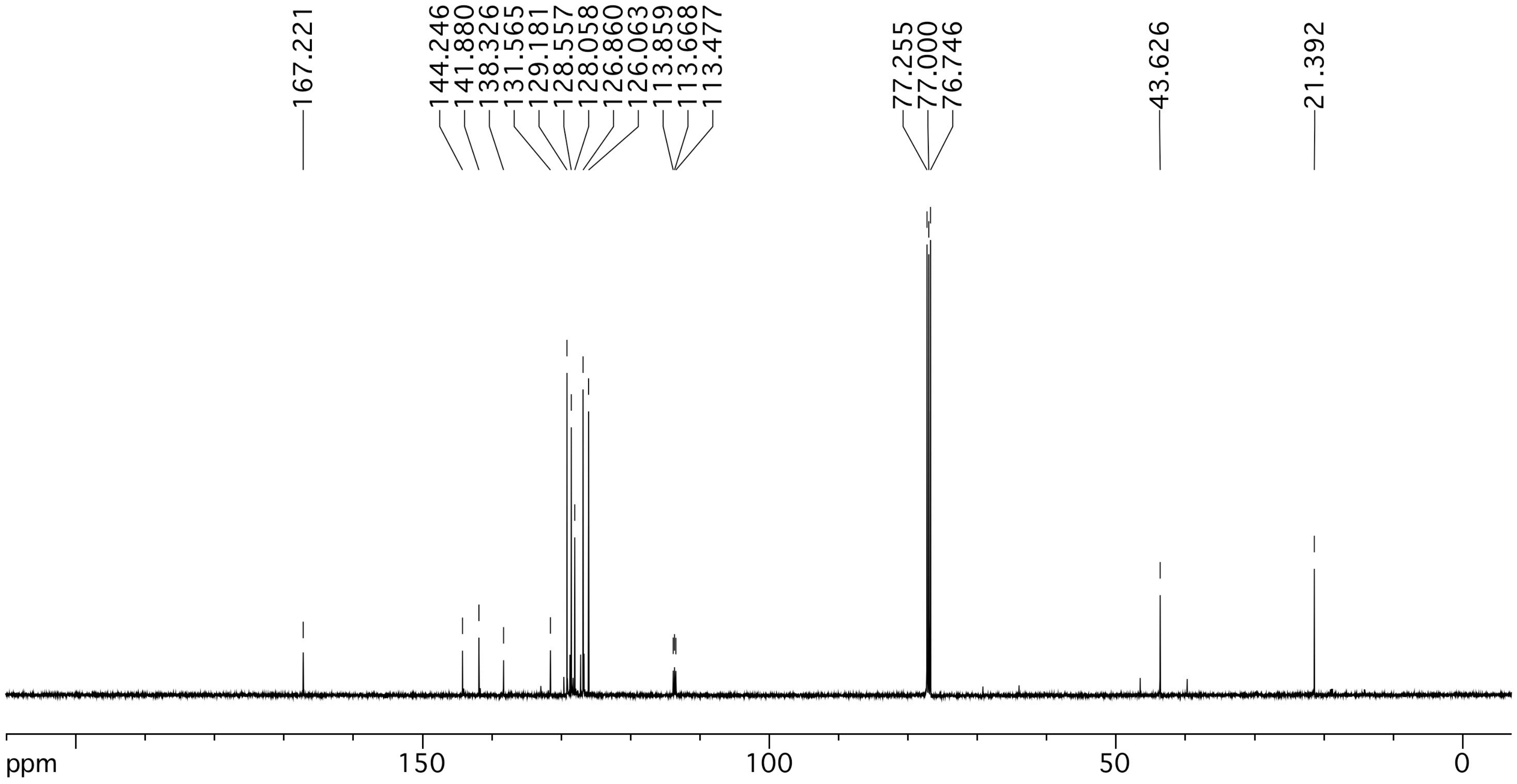
167.221
144.246
141.880
138.326
131.565
129.181
128.557
128.058
126.860
126.063
113.859
113.668
113.477
77.255
77.000
76.746
43.626
21.392
ppm
150
100
50
0

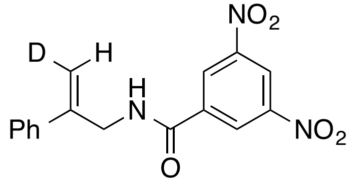
NO2
D
H
H
N
Ph
O
NO2

11b-D

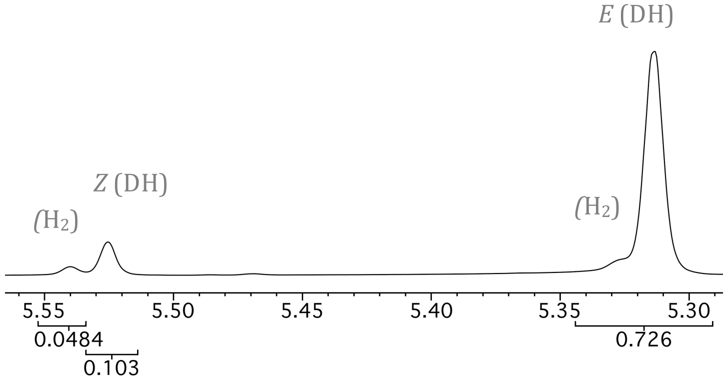
E (DH)
Z (DH)
(H2)
(H2)
5.55
5.50
5.45
5.40
5.35
5.30
0.0484
0.103
0.726

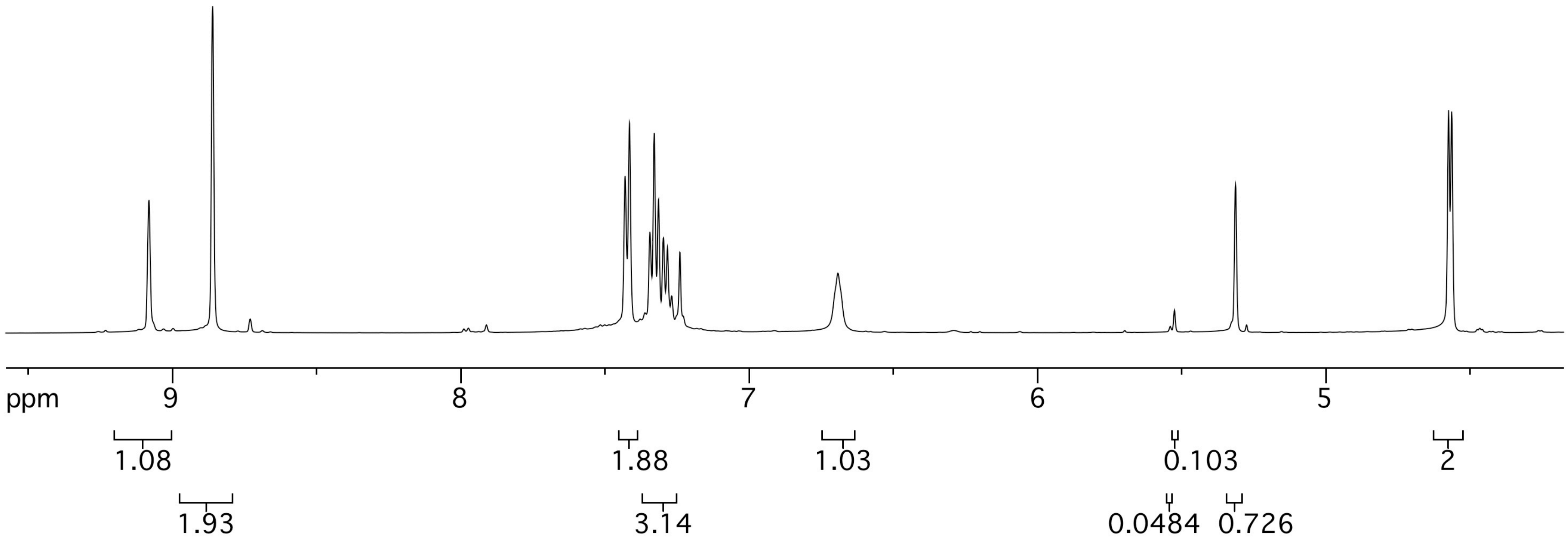
ppm
9
8
7
6
5
1.08
1.93
1.88
3.14
1.03
0.103
0.0484
0.726
2

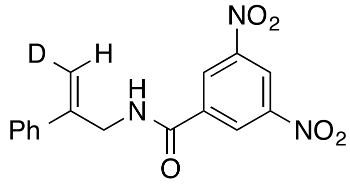

NO2
D
H
H
N
Ph
NO2
O

11b-D

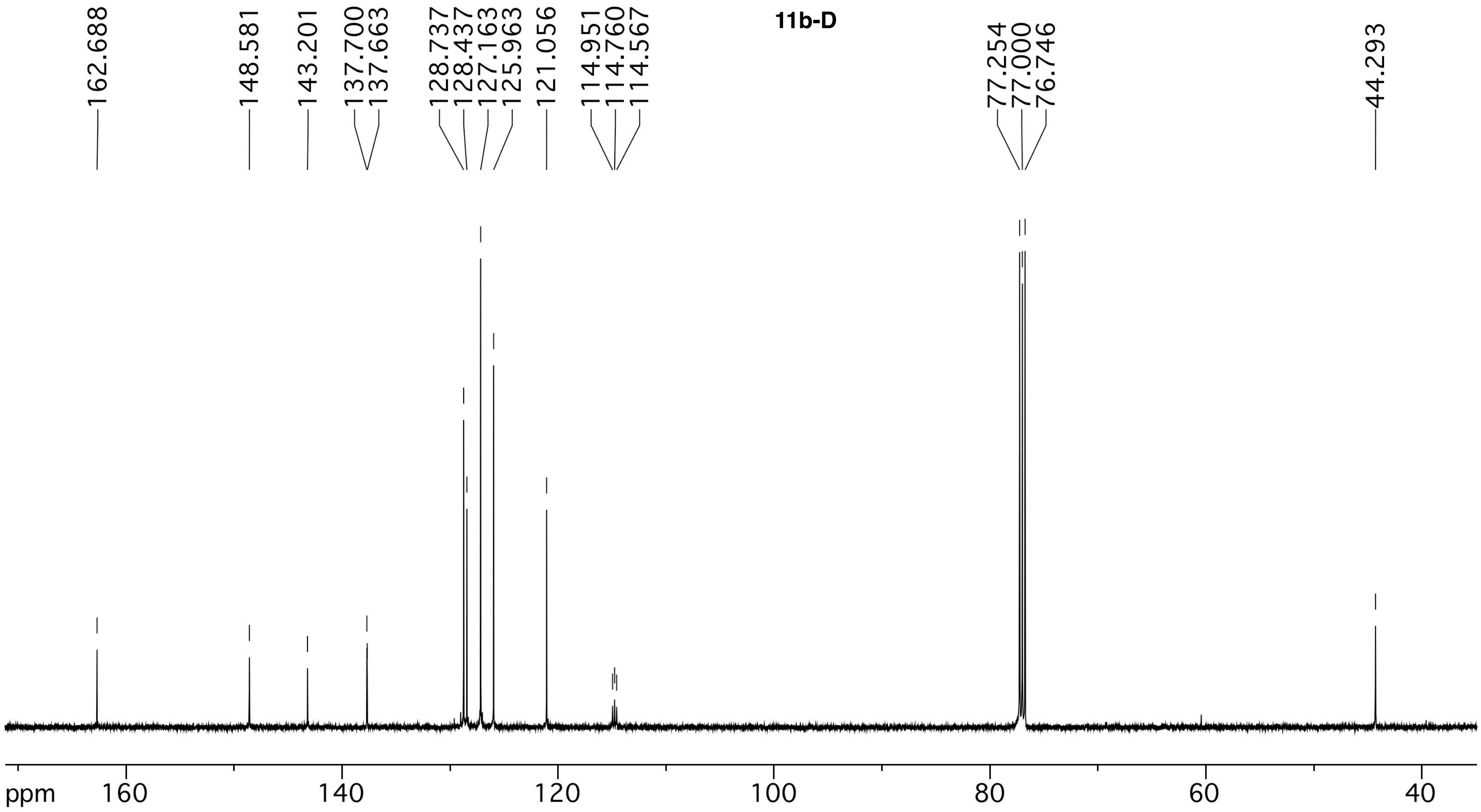

162.688
148.581
143.201
137.700
137.663
128.737
128.437
127.163
125.963
121.056
114.951
114.760
114.567
77.254
77.000
76.746
44.293
ppm
160
140
120
100
80
60
40

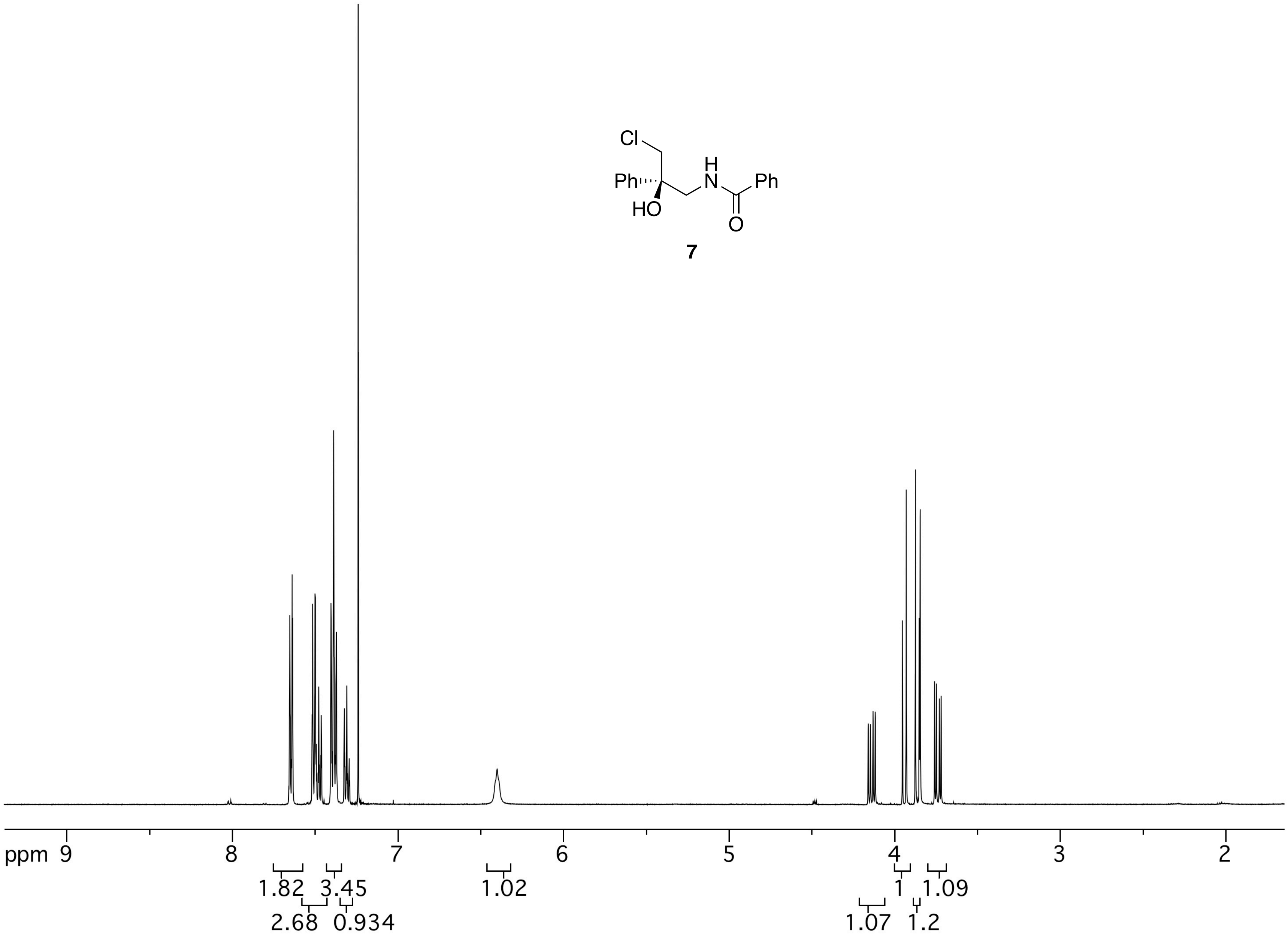
Cl
H
Ph
N
Ph
HO
O
7
ppm
9
8
7
6
5
4
3
2
1.82
3.45
2.68
0.934
1.02
1
1.09
1.07
1.2

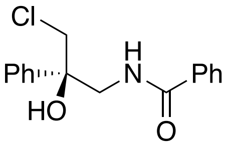
Cl
Ph
HO
H
N
Ph
O

7

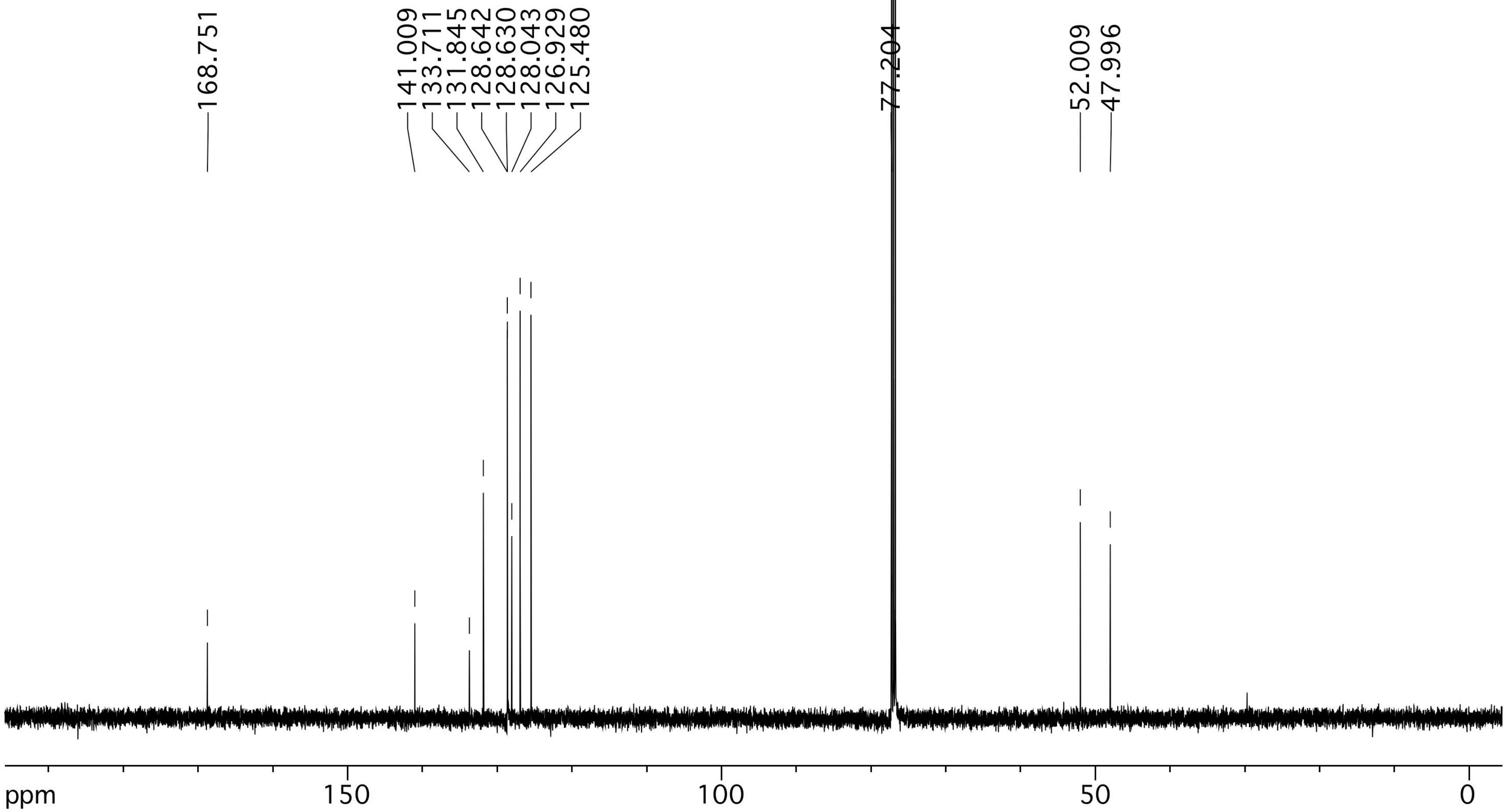
168.751
141.009
133.711
131.845
128.642
128.630
128.043
126.929
125.480
77.204
52.009
47.996
ppm
150
100
50
0

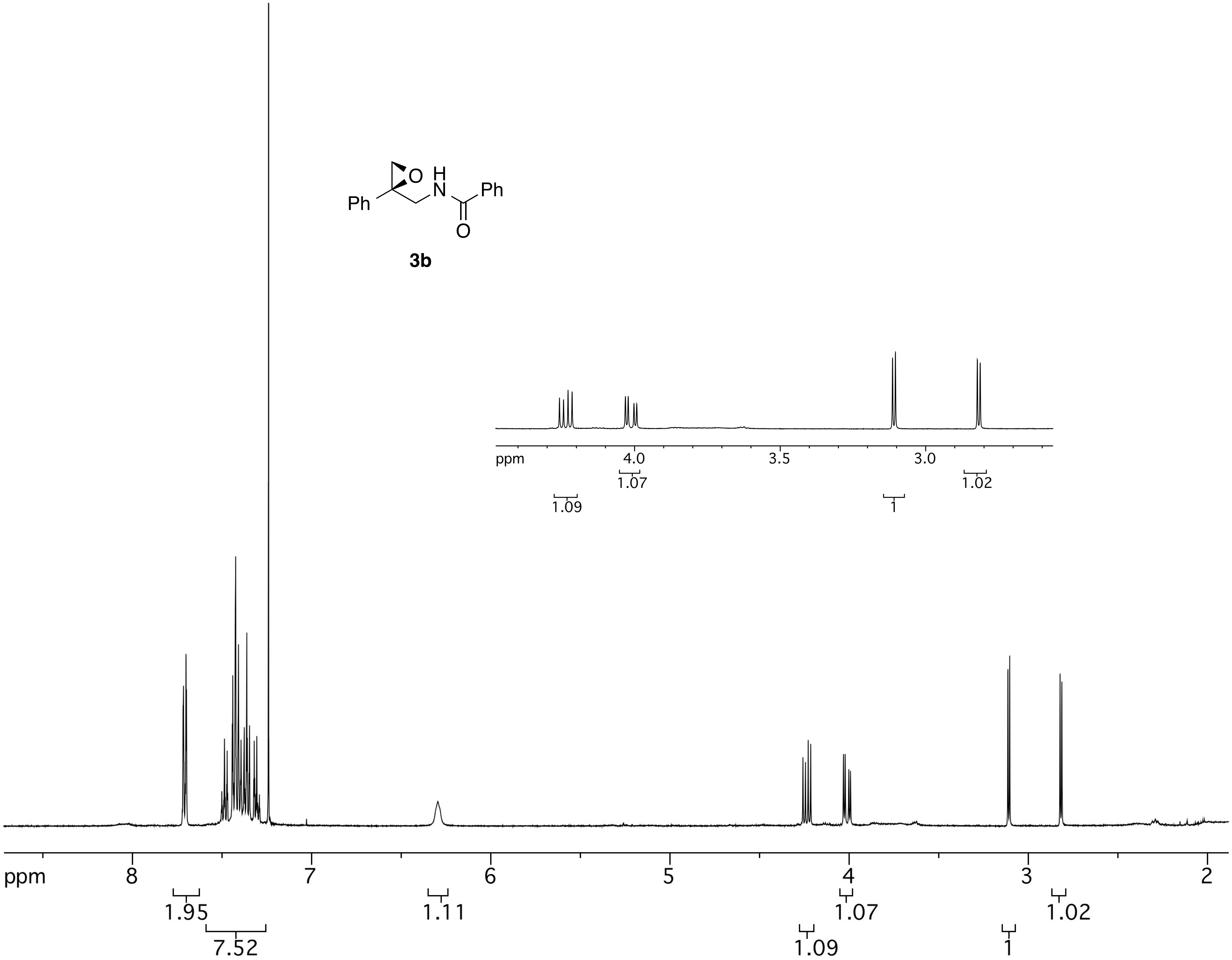

O
H
N
Ph
Ph
O
3b
ppm
4.0
3.5
3.0
1.07
1.02
1.09
1
ppm
8
7
6
5
4
3
2
1.95
7.52
1.11
1.07
1.09
1
1.02

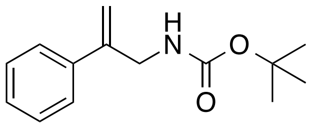

1c

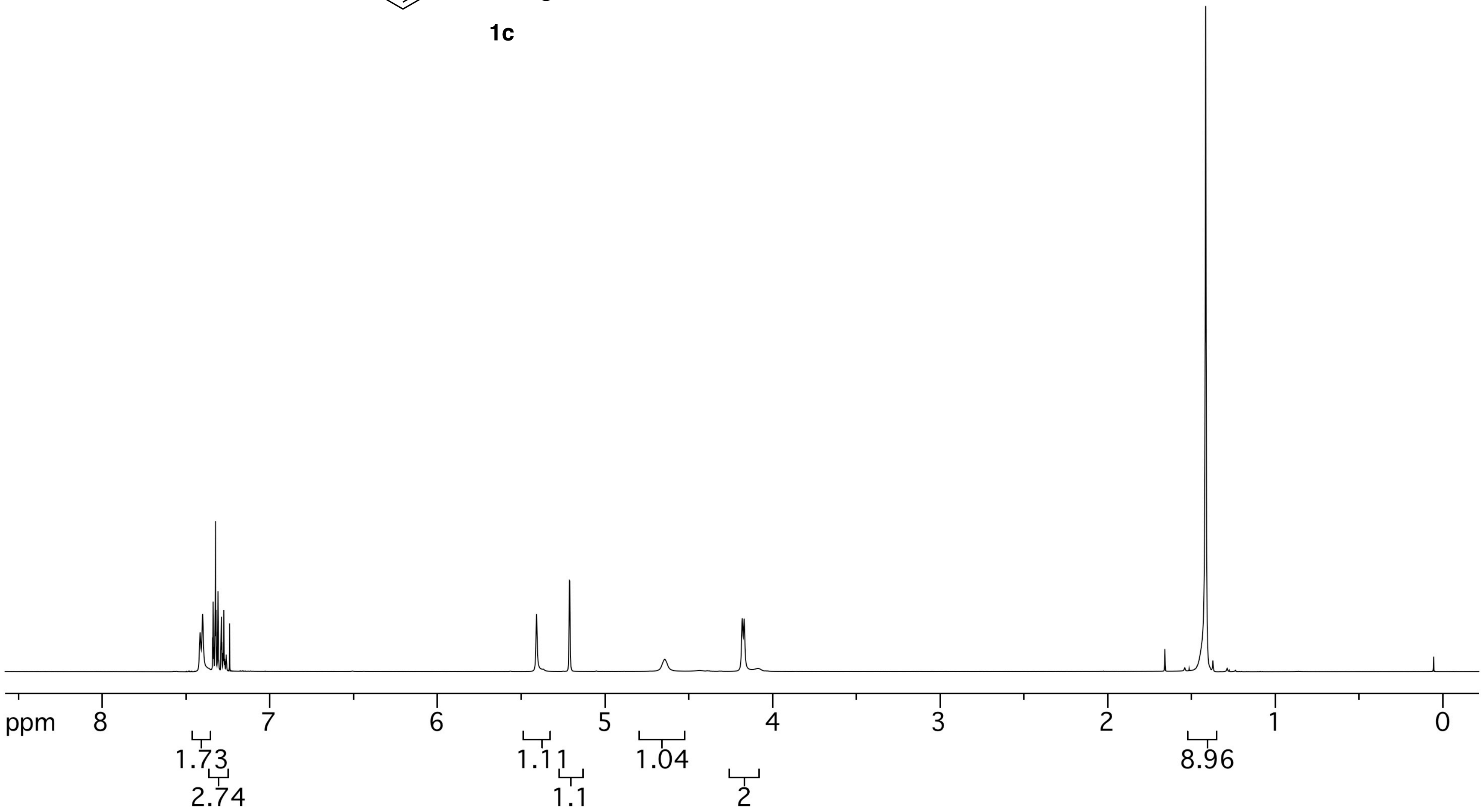

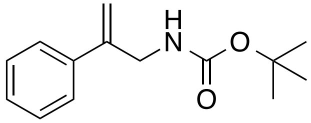

1c

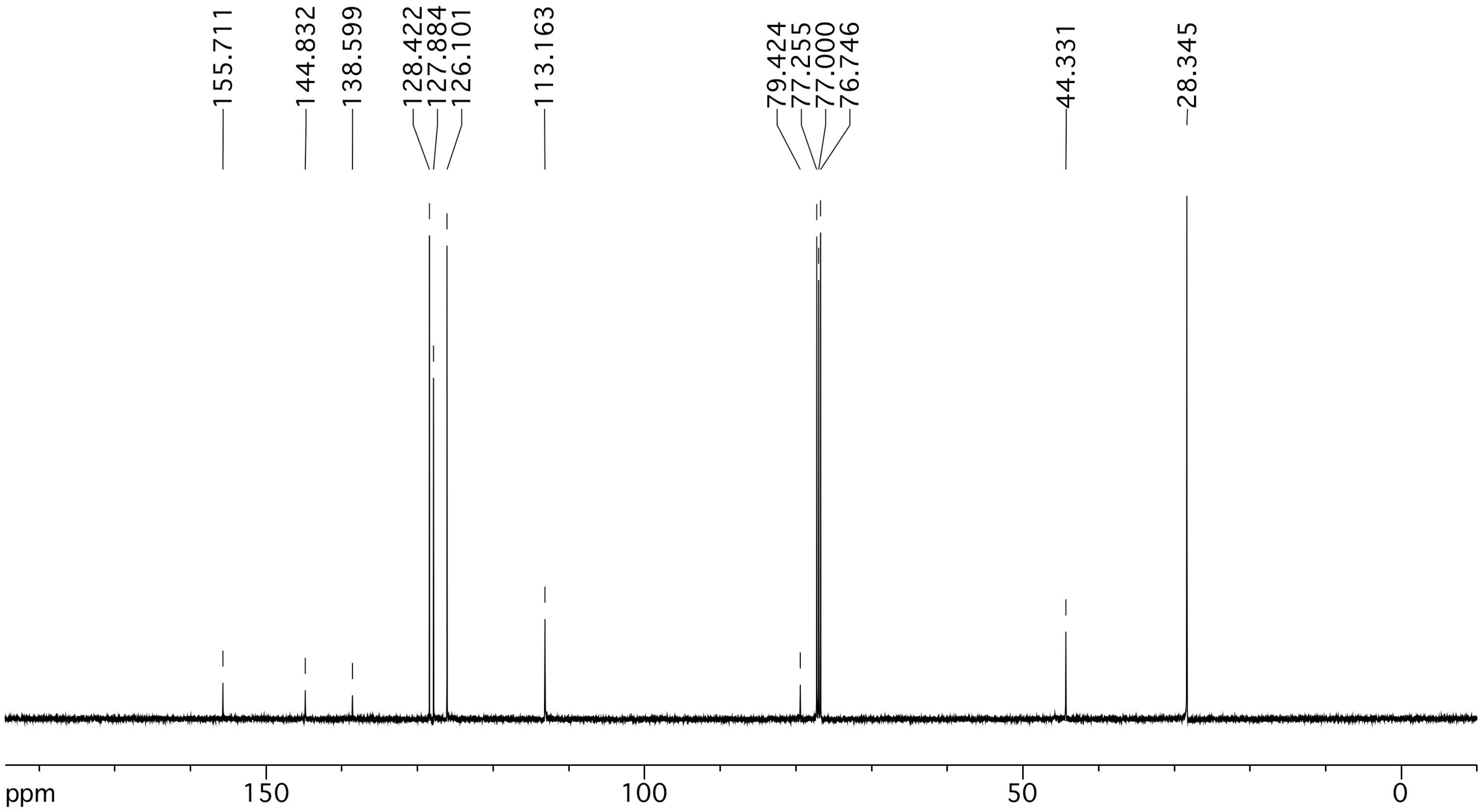

155.711
144.832
138.599
128.422
127.884
126.101
113.163
79.424
77.255
77.000
76.746
44.331
28.345
ppm
150
100
50
0

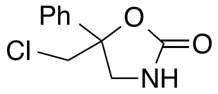

2c

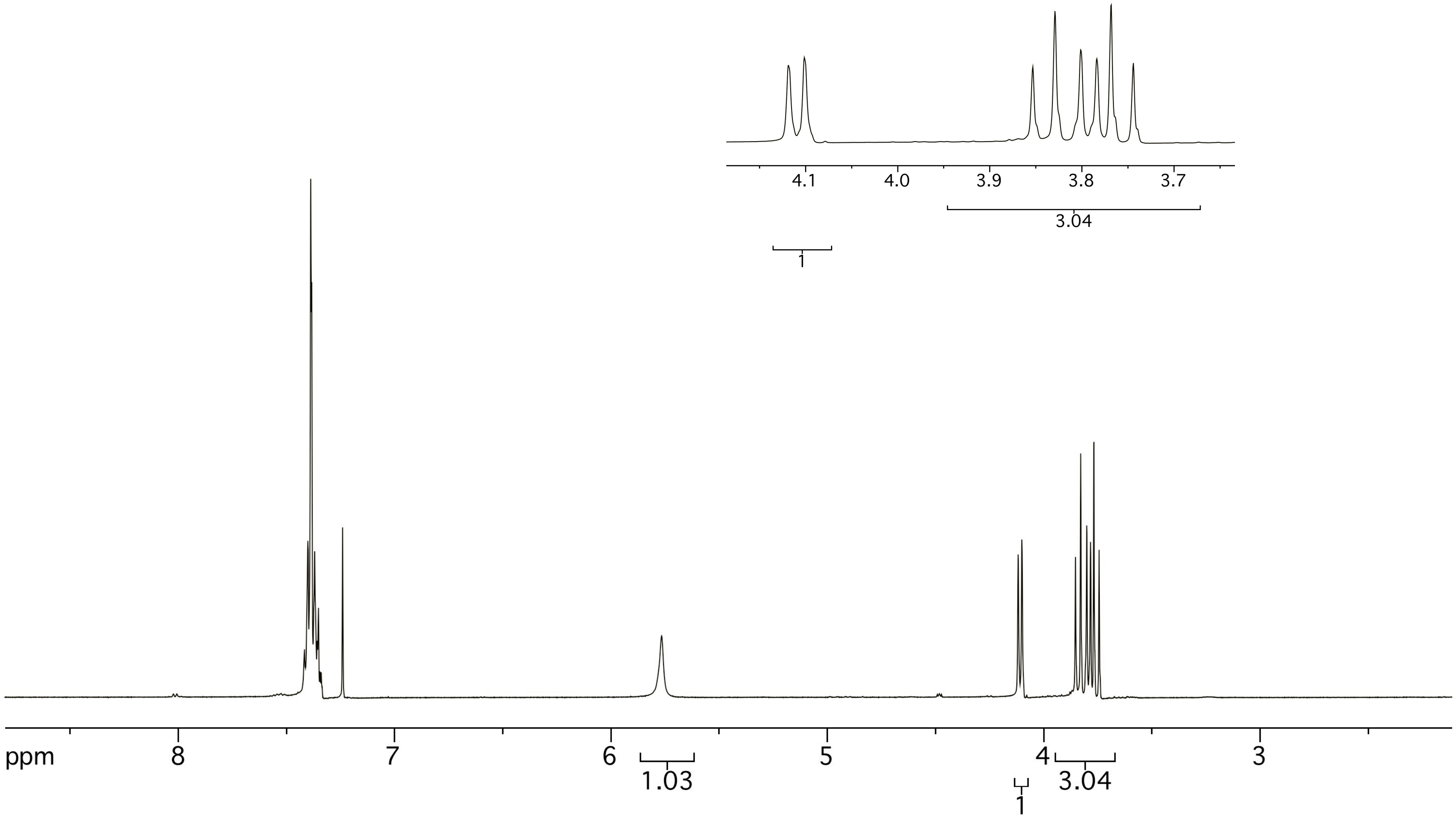

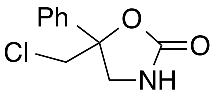

Ph
O
O
Cl
NH

2c

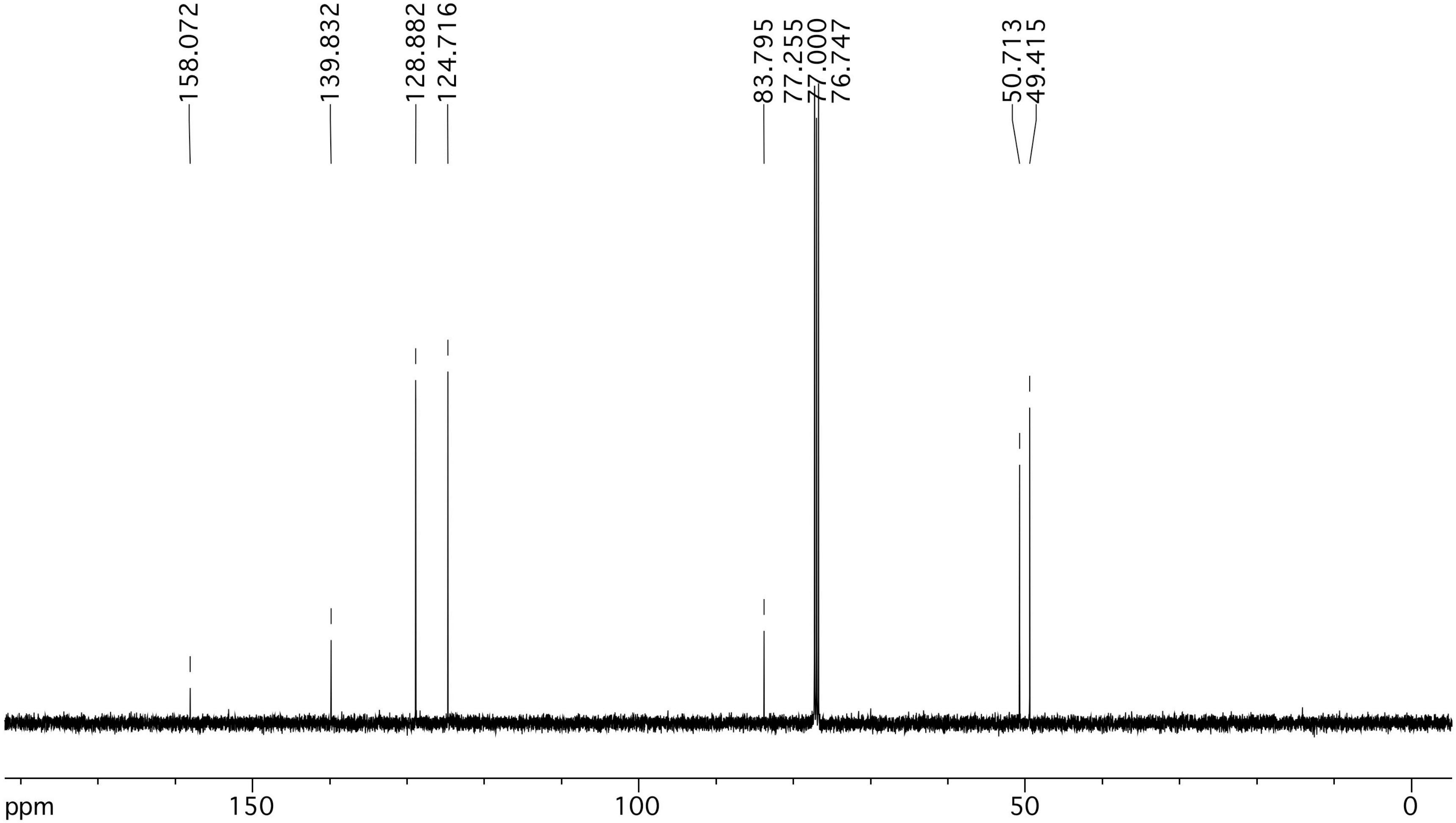

158.072
139.832
128.882
124.716
83.795
77.255
77.000
76.747
50.713
49.415
ppm
150
100
50
0

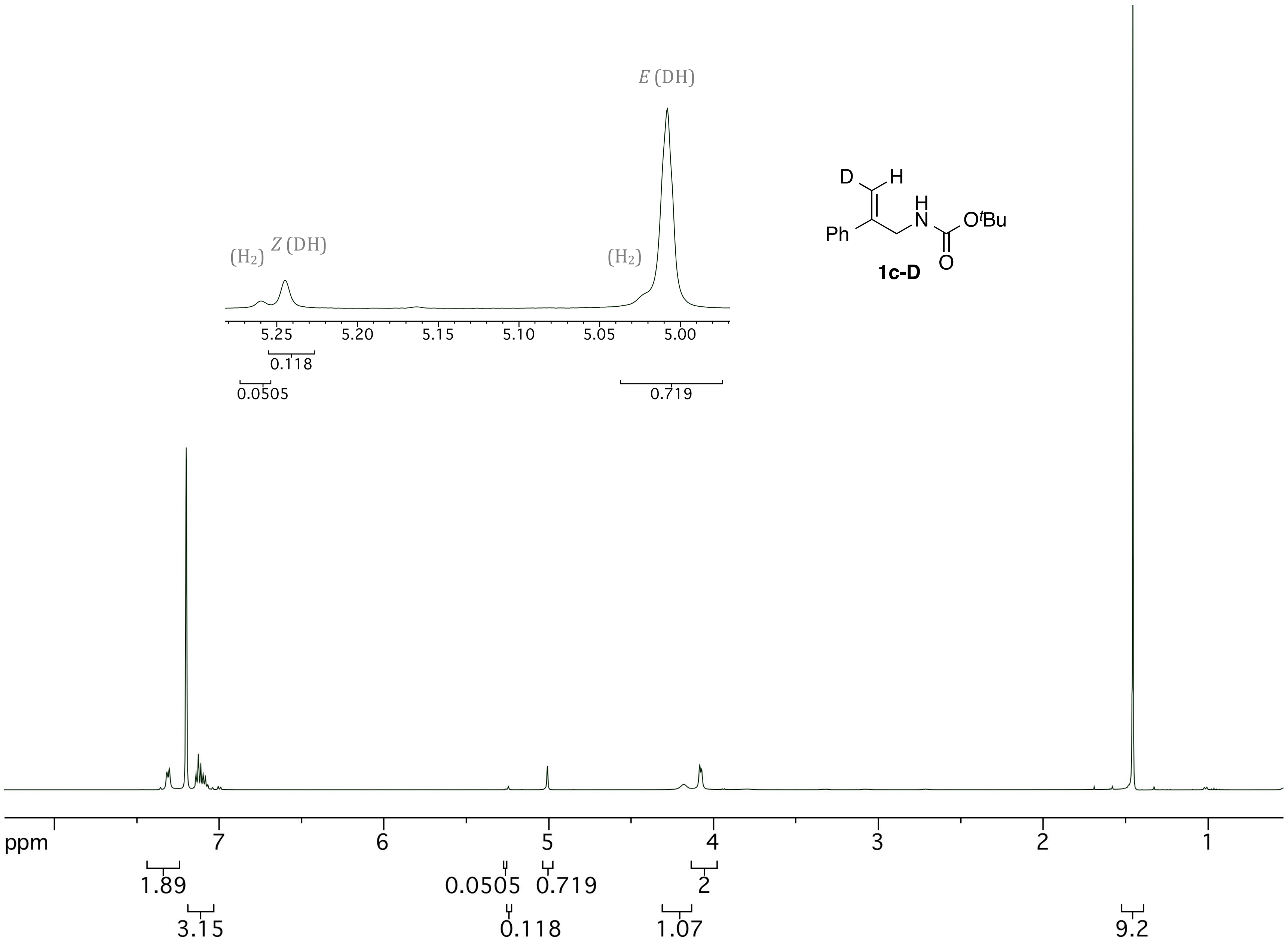
E (DH)
(H₂) Z (DH)
(H₂)
5.25 5.20 5.15 5.10 5.05 5.00
0.118
0.0505
0.719
D H
H
N
Ph
O^tBu
O
1c-D
ppm 7 6 5 4 3 2 1
1.89
3.15
0.0505 0.719
0.118
2
1.07
9.2

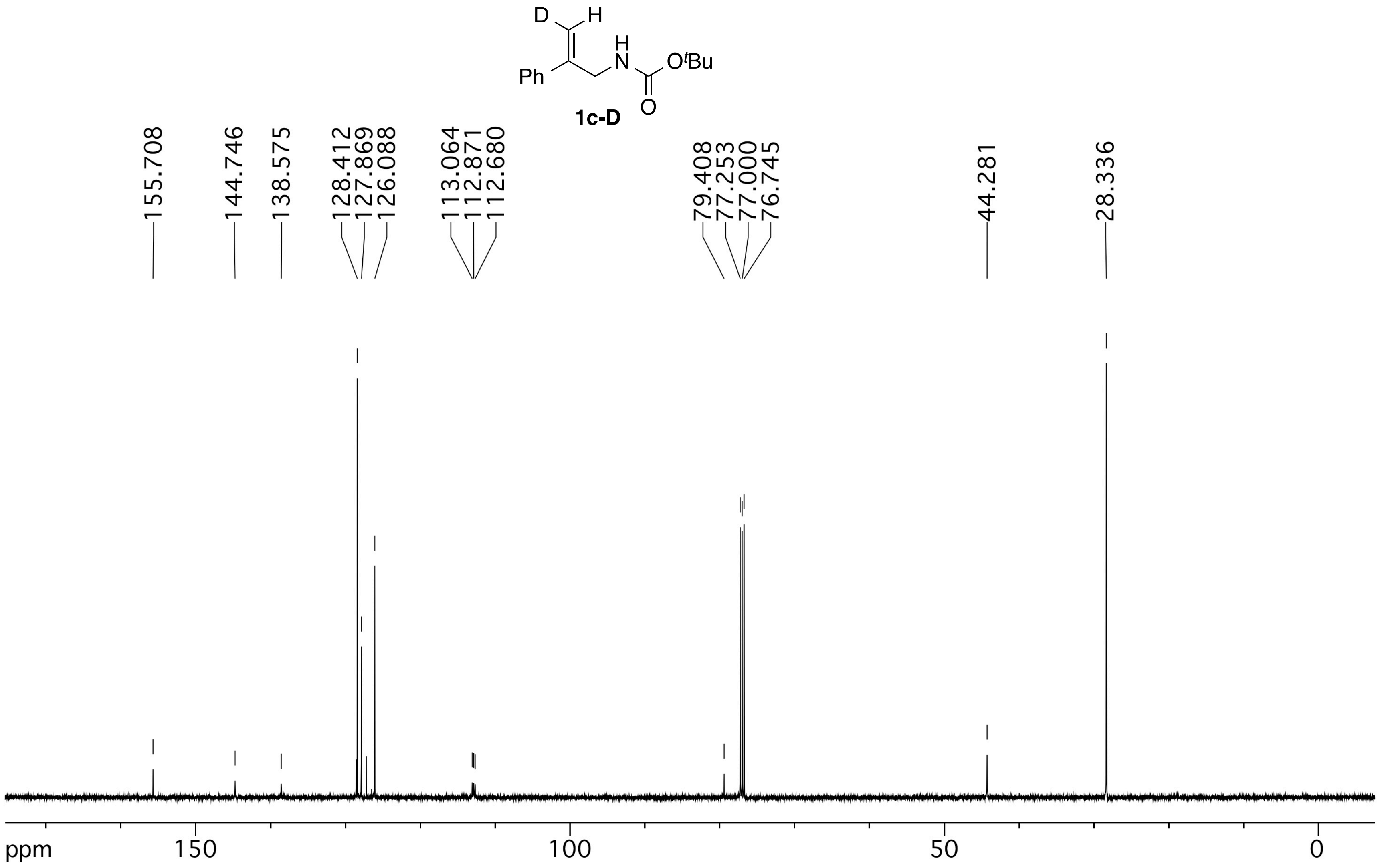
D
H
H
N
O
tBu
Ph
O
1c-D
155.708
144.746
138.575
128.412
127.869
126.088
113.064
112.871
112.680
79.408
77.253
77.000
76.745
44.281
28.336
ppm
150
100
50
0

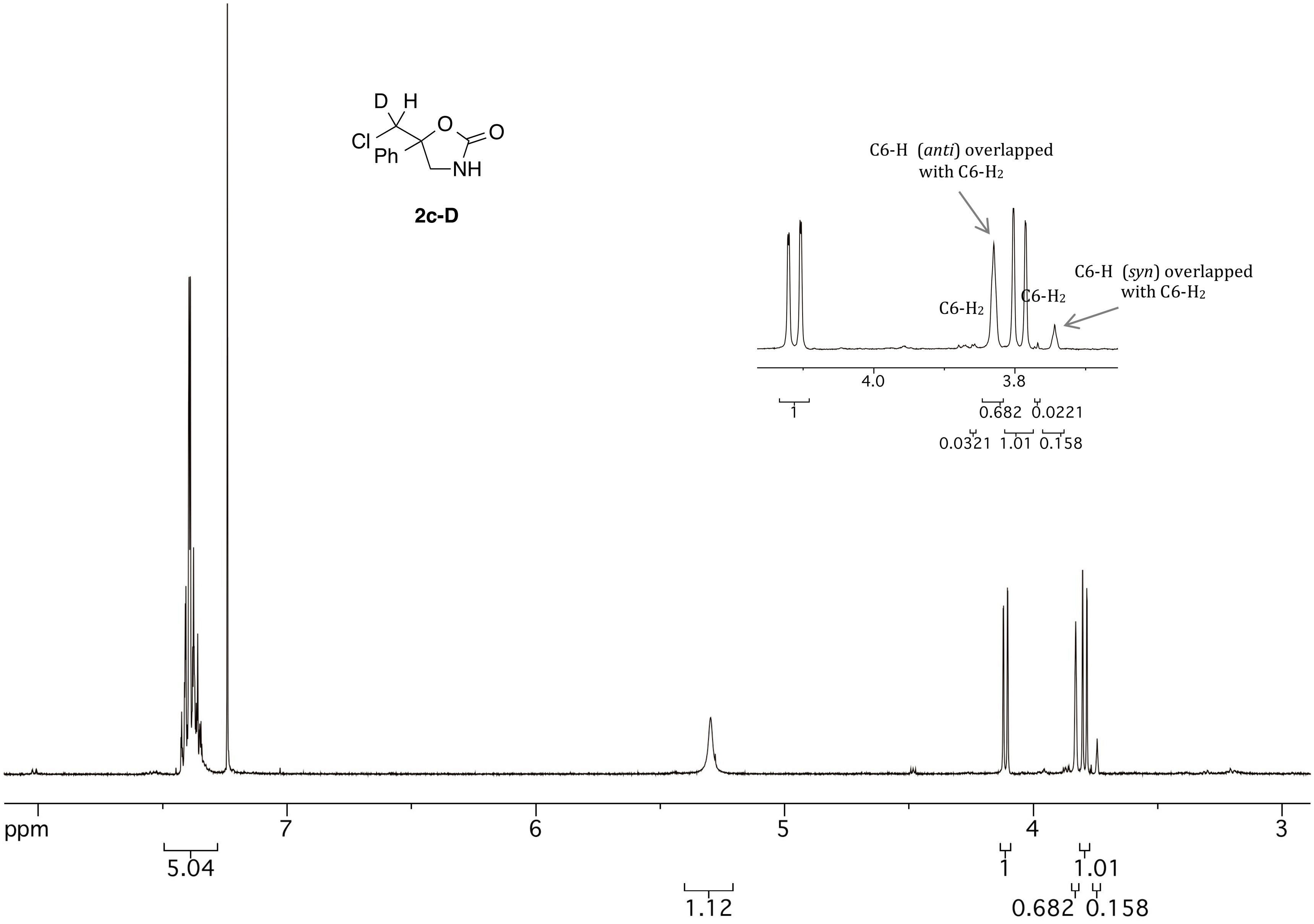

D H
Cl
Ph
O
O
NH
2c-D
C6-H (anti) overlapped
with C6-H2
C6-H (syn) overlapped
with C6-H2
C6-H2
C6-H2
4.0
3.8
1
0.682
0.0221
0.0321
1.01
0.158
ppm
7
6
5
4
3
5.04
1.12
1
1.01
0.682
0.158

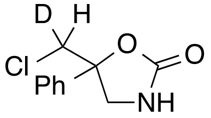
D
H
O
O
Cl
Ph
NH

2c-D

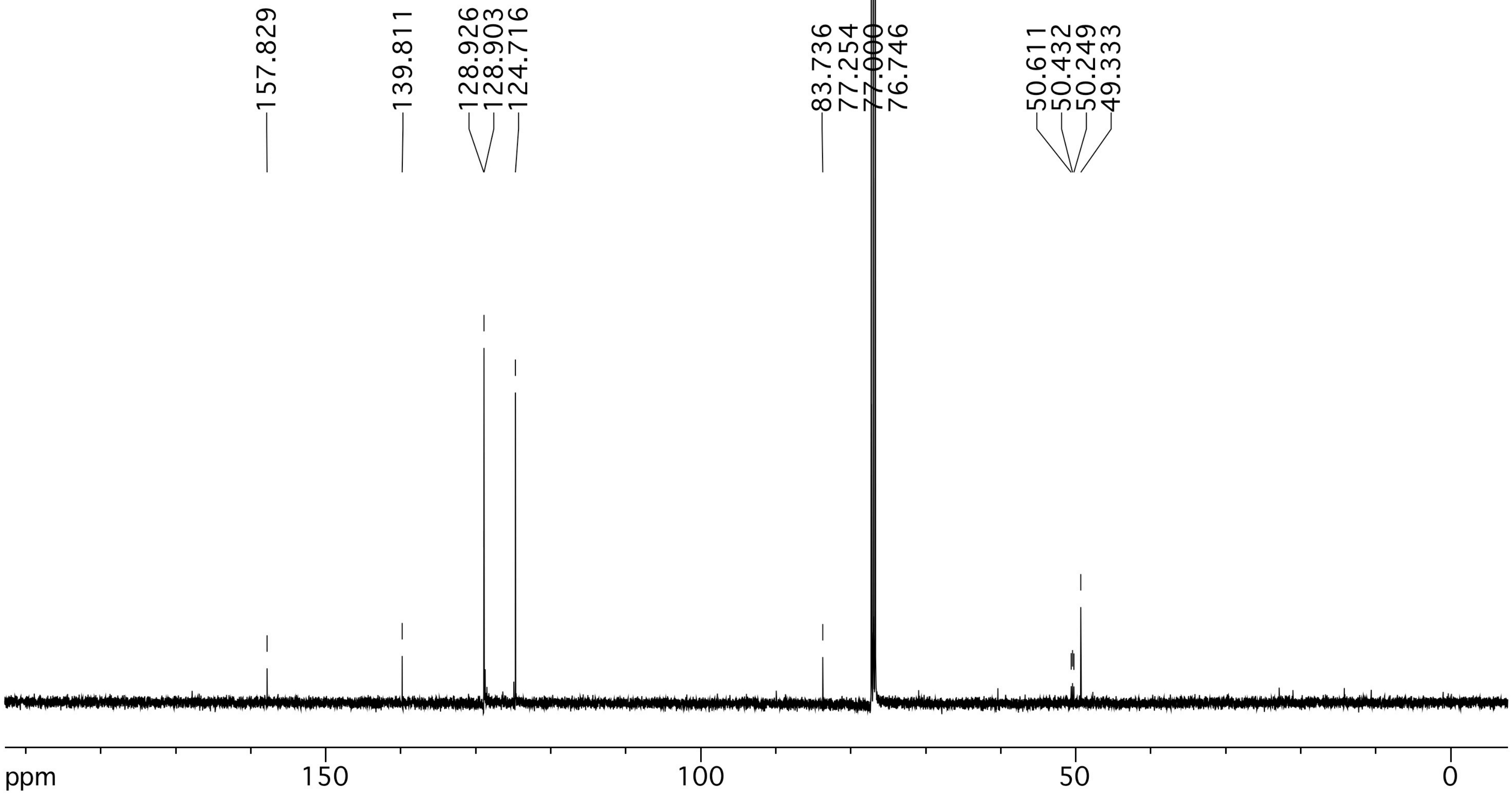
157.829
139.811
128.926
128.903
124.716
83.736
77.254
77.000
76.746
50.611
50.432
50.249
49.333
ppm
150
100
50
0

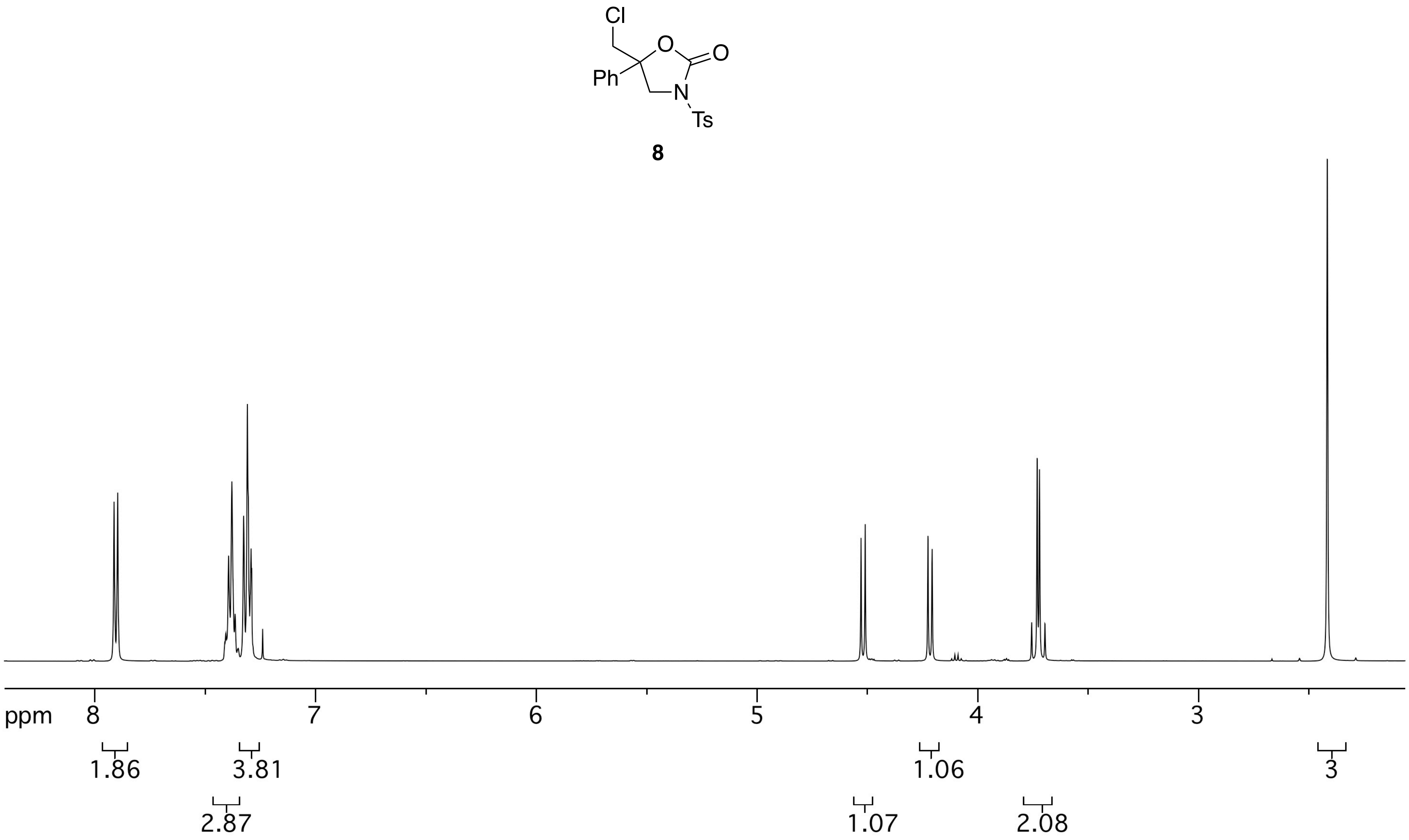

Cl
O
O
Ph
N
Ts
8
ppm
8
7
6
5
4
3
1.86
3.81
2.87
1.07
1.06
2.08
3

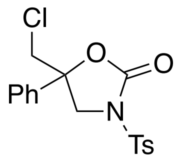
Cl
O
O
Ph
N
Ts

8

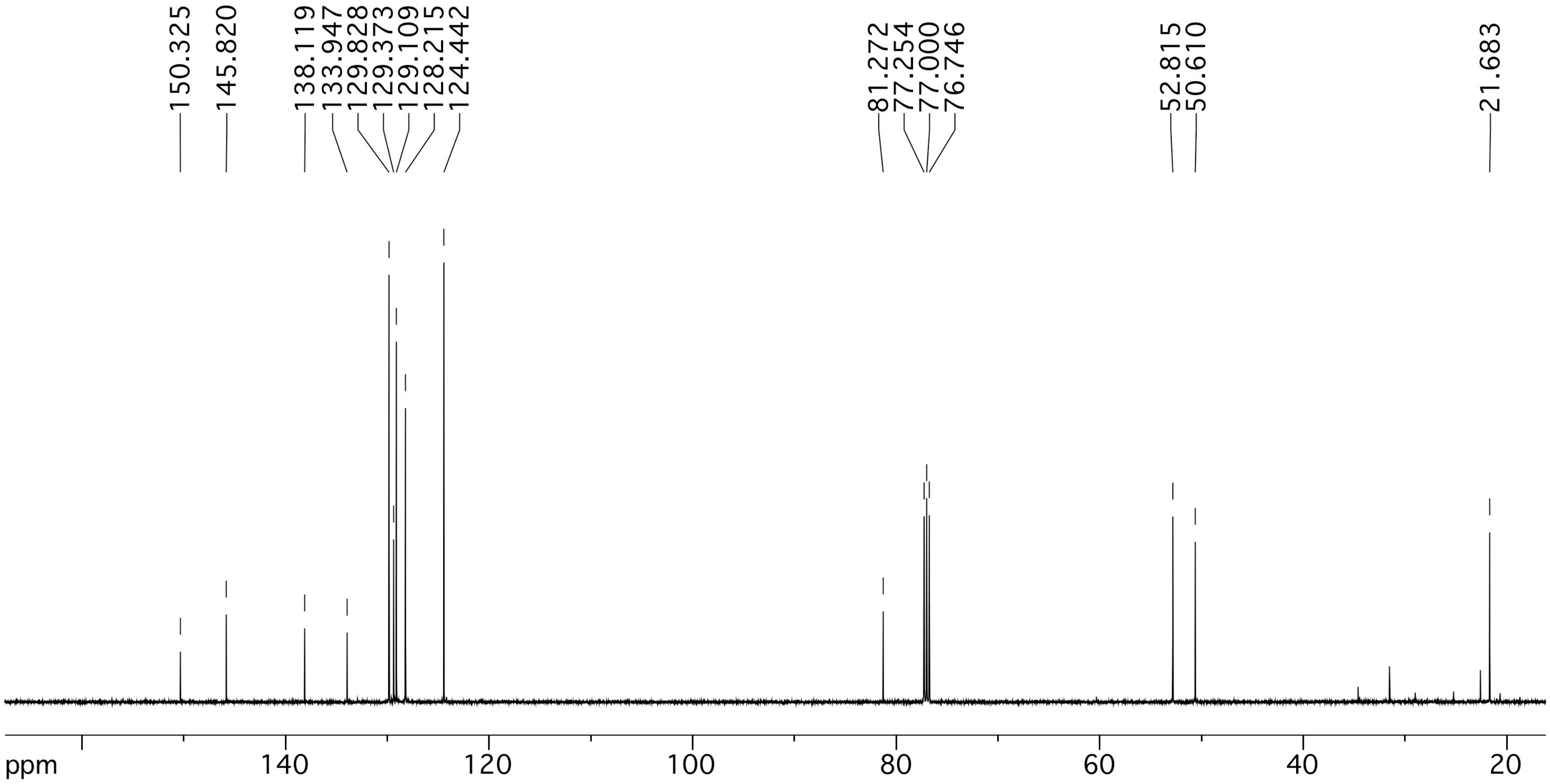
150.325
145.820
138.119
133.947
129.828
129.373
129.109
128.215
124.442
81.272
77.254
77.000
76.746
52.815
50.610
21.683
ppm
140
120
100
80
60
40
20

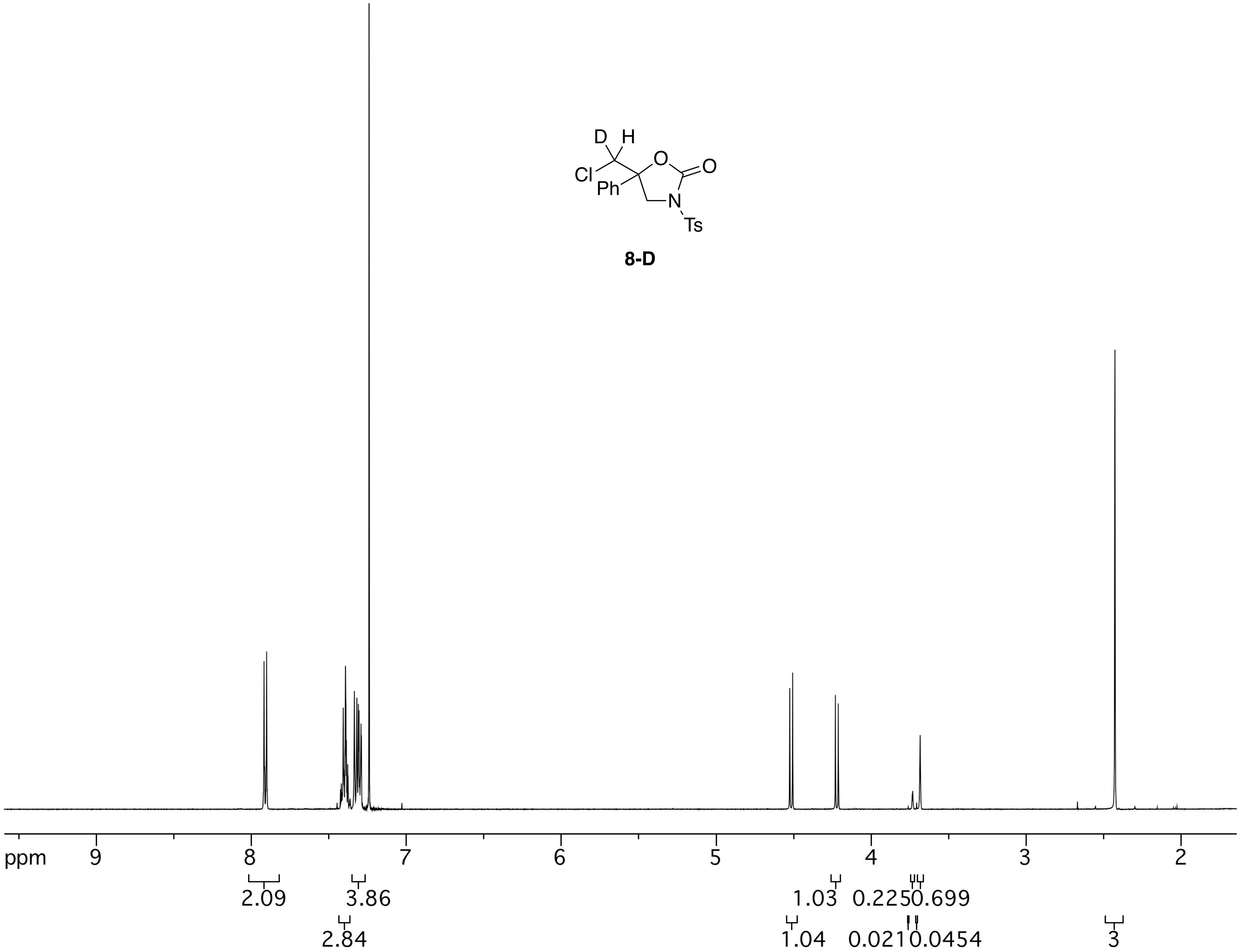
D
H
O
O
Cl
Ph
N
Ts
8-D
ppm
9
8
7
6
5
4
3
2
2.09
3.86
2.84
1.03
0.225
0.699
1.04
0.021
0.0454
3

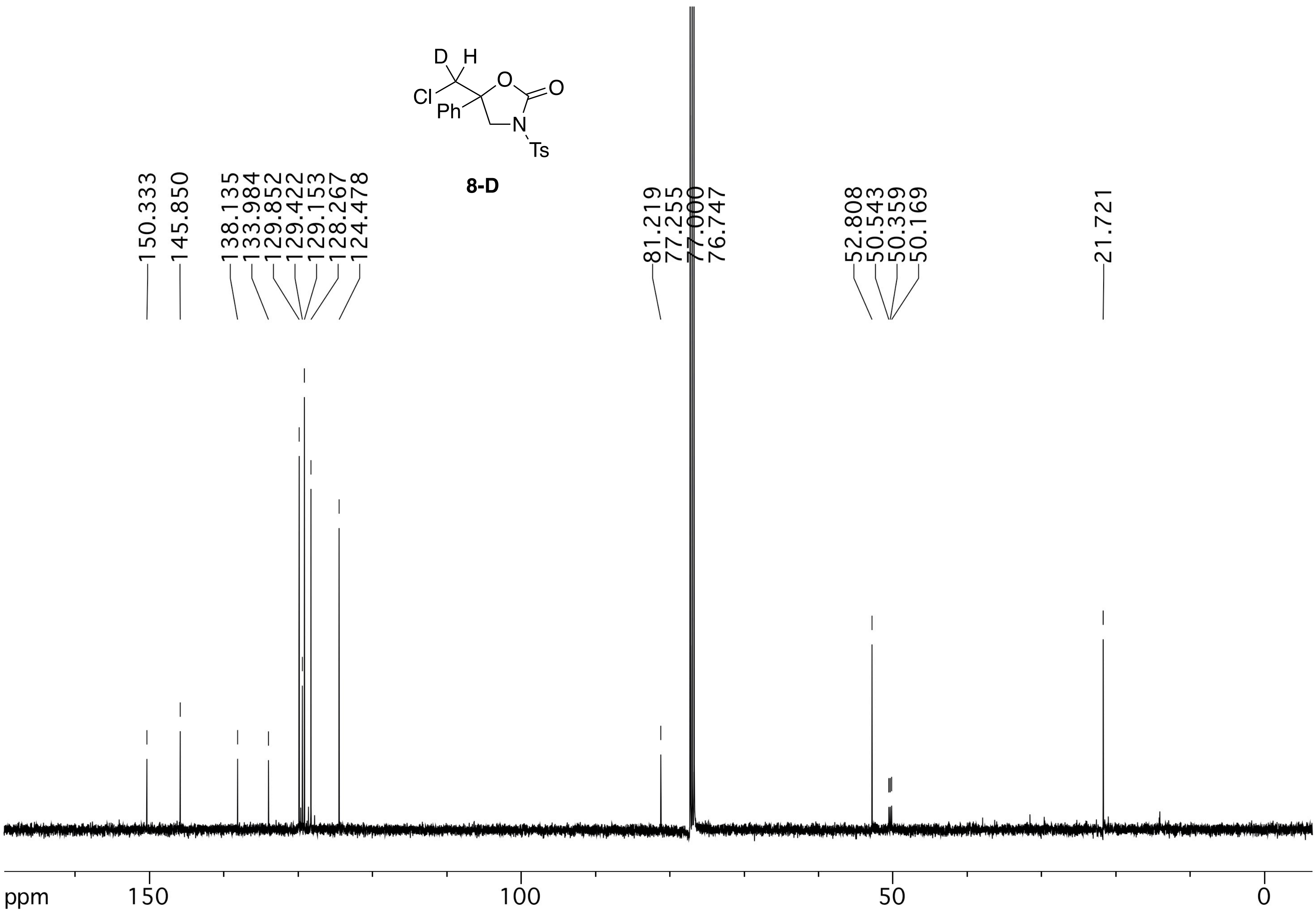
D H
O
O
Cl
Ph
N
Ts
8-D
150.333
145.850
138.135
133.984
129.852
129.422
129.153
128.267
124.478
81.219
77.255
77.000
76.747
52.808
50.543
50.359
50.169
21.721
ppm
150
100
50
0

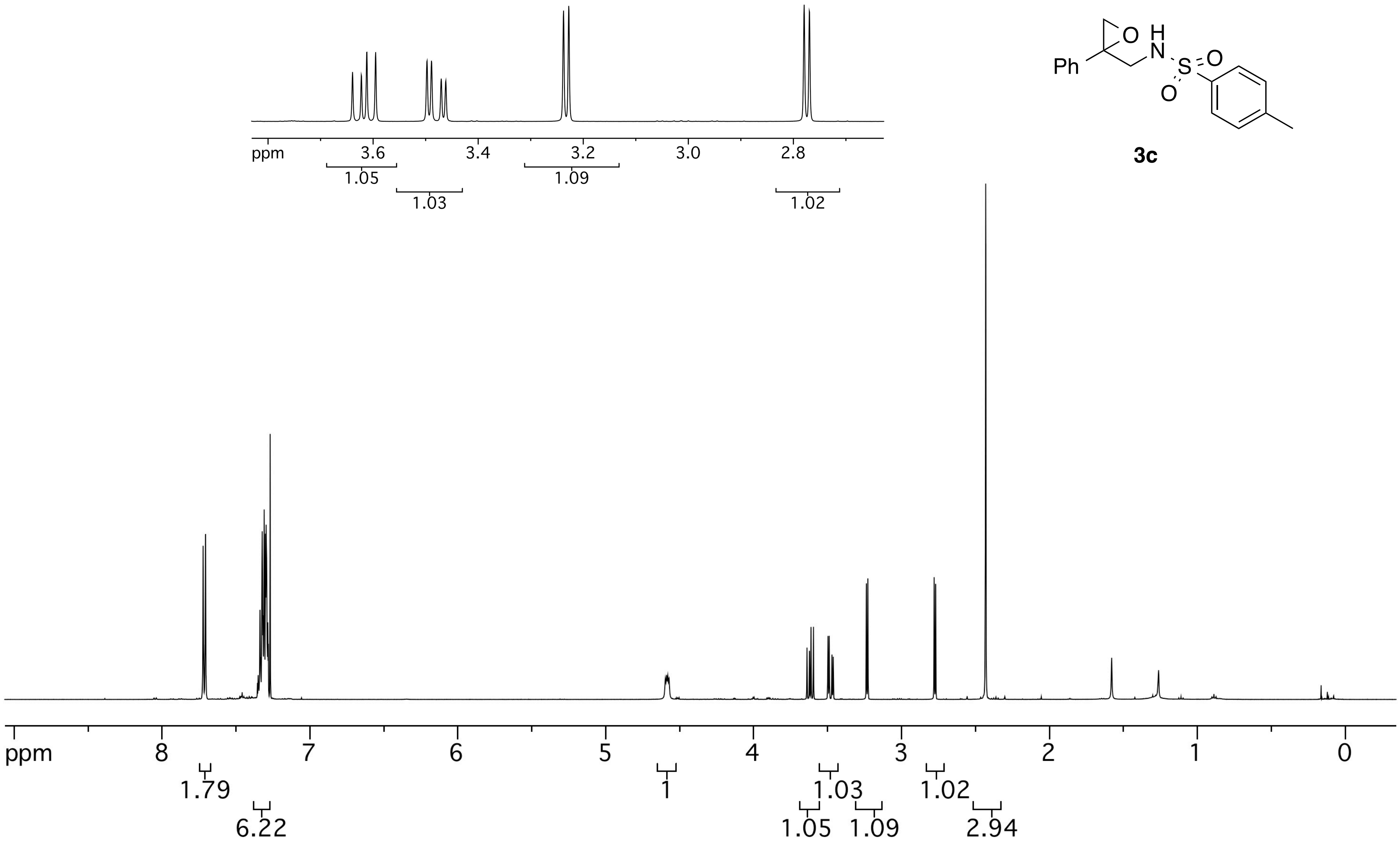
Ph
O
H
N
S
O
O
ppm
3.6
3.4
3.2
3.0
2.8
1.05
1.03
1.09
1.02
ppm
8
7
6
5
4
3
2
1
0
1.79
6.22
1
1.03
1.05
1.09
1.02
2.94

3c

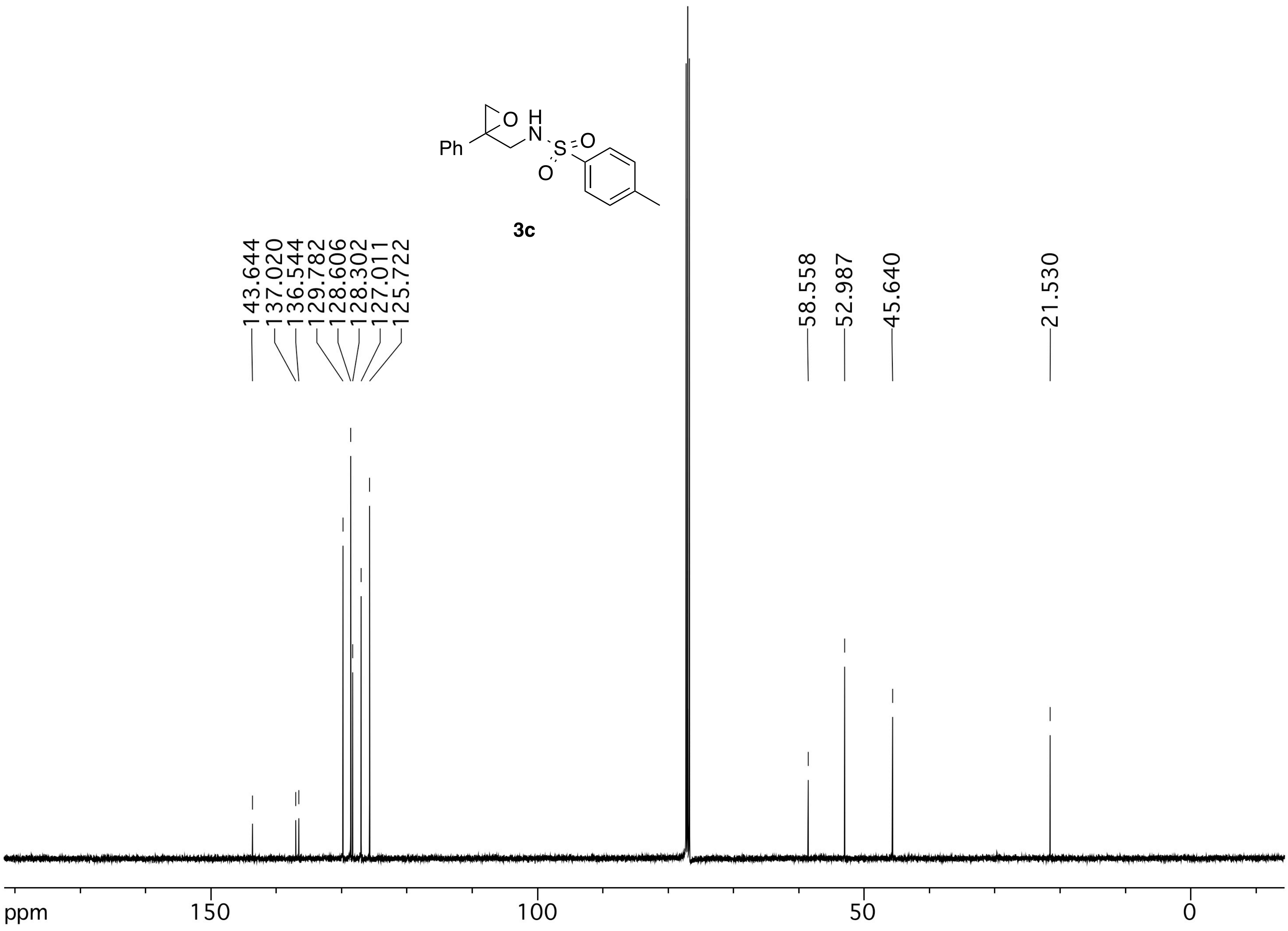
Ph
O
H
N
S
O
O
3c
143.644
137.020
136.544
129.782
128.606
128.302
127.011
125.722
58.558
52.987
45.640
21.530
ppm
150
100
50
0

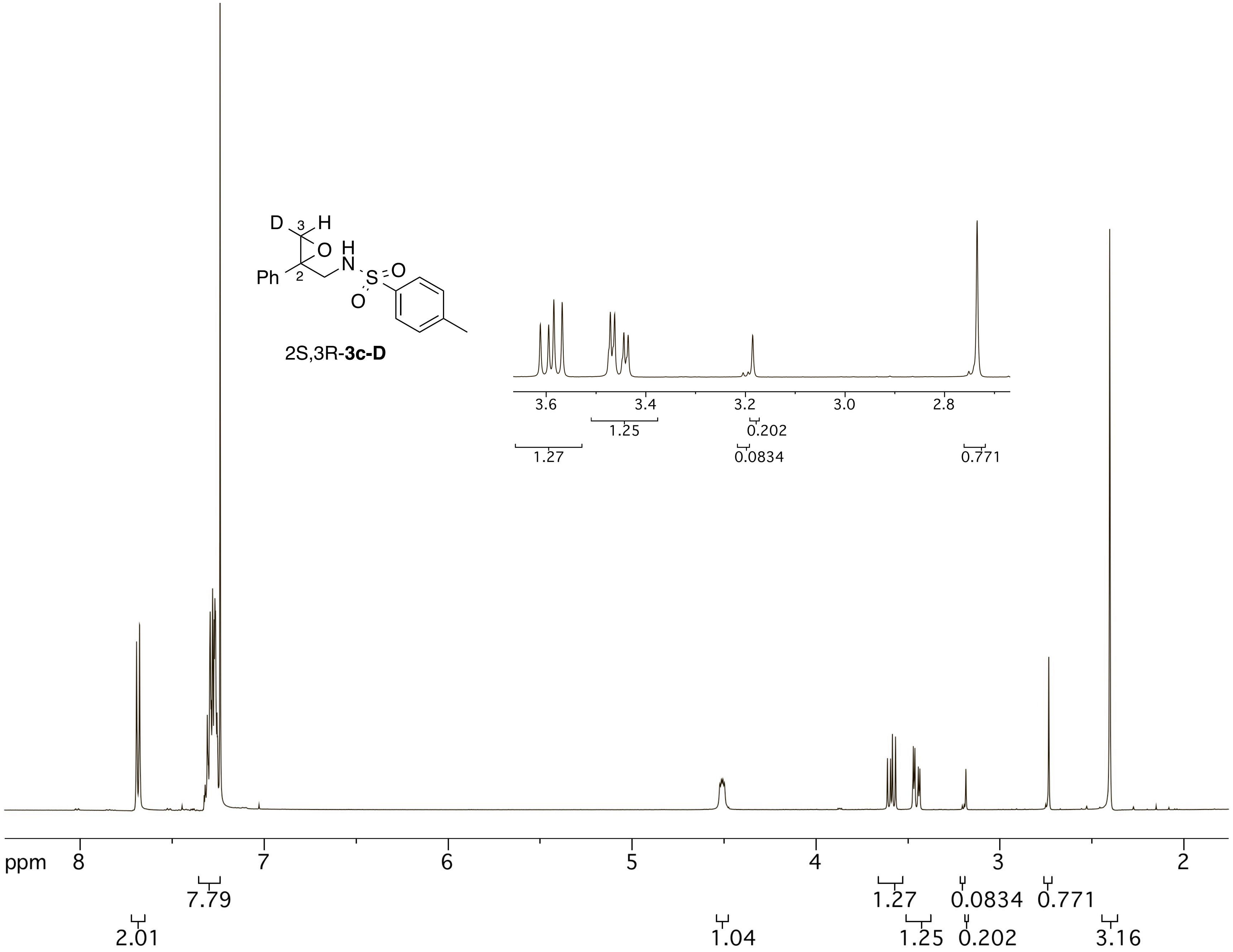
D
3
H
O
H
N
S
O
O
Ph
2
2S,3R-**3c-D**
3.6
3.4
3.2
3.0
2.8
1.25
0.202
1.27
0.0834
0.771
ppm
8
7
6
5
4
3
2
7.79
2.01
1.04
1.27
1.25
0.0834
0.202
0.771
3.16

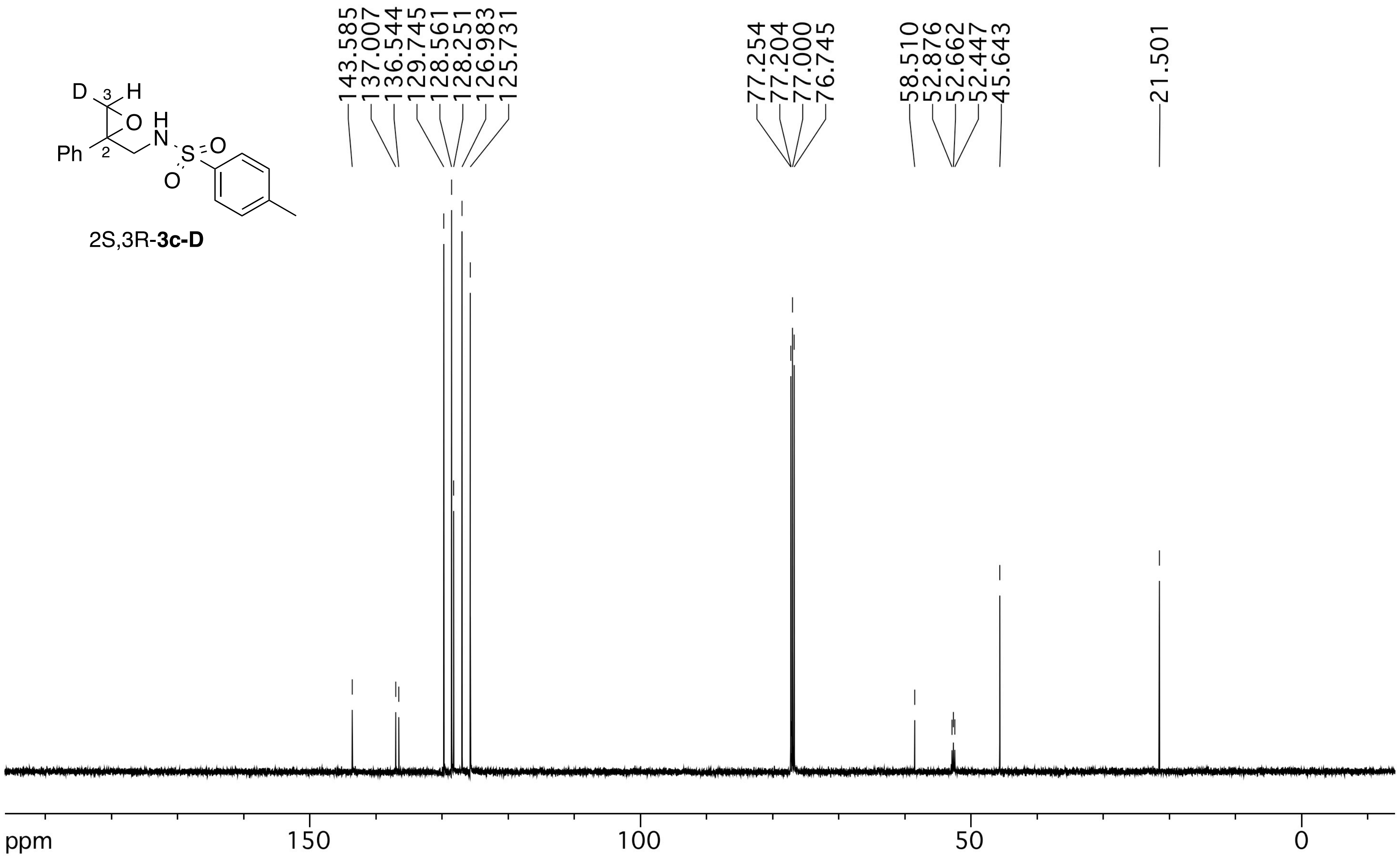
D
3
H
O
H
N
S
O
O
Ph
2
2S,3R-3c-D
143.585
137.007
136.544
129.745
128.561
128.251
126.983
125.731
77.254
77.204
77.000
76.745
58.510
52.876
52.662
52.447
45.643
21.501
ppm
150
100
50
0

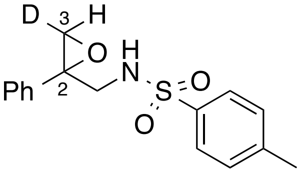
D
3
H
O
H
N
S
O
O
Ph
2

2R,3R-**3c-D**

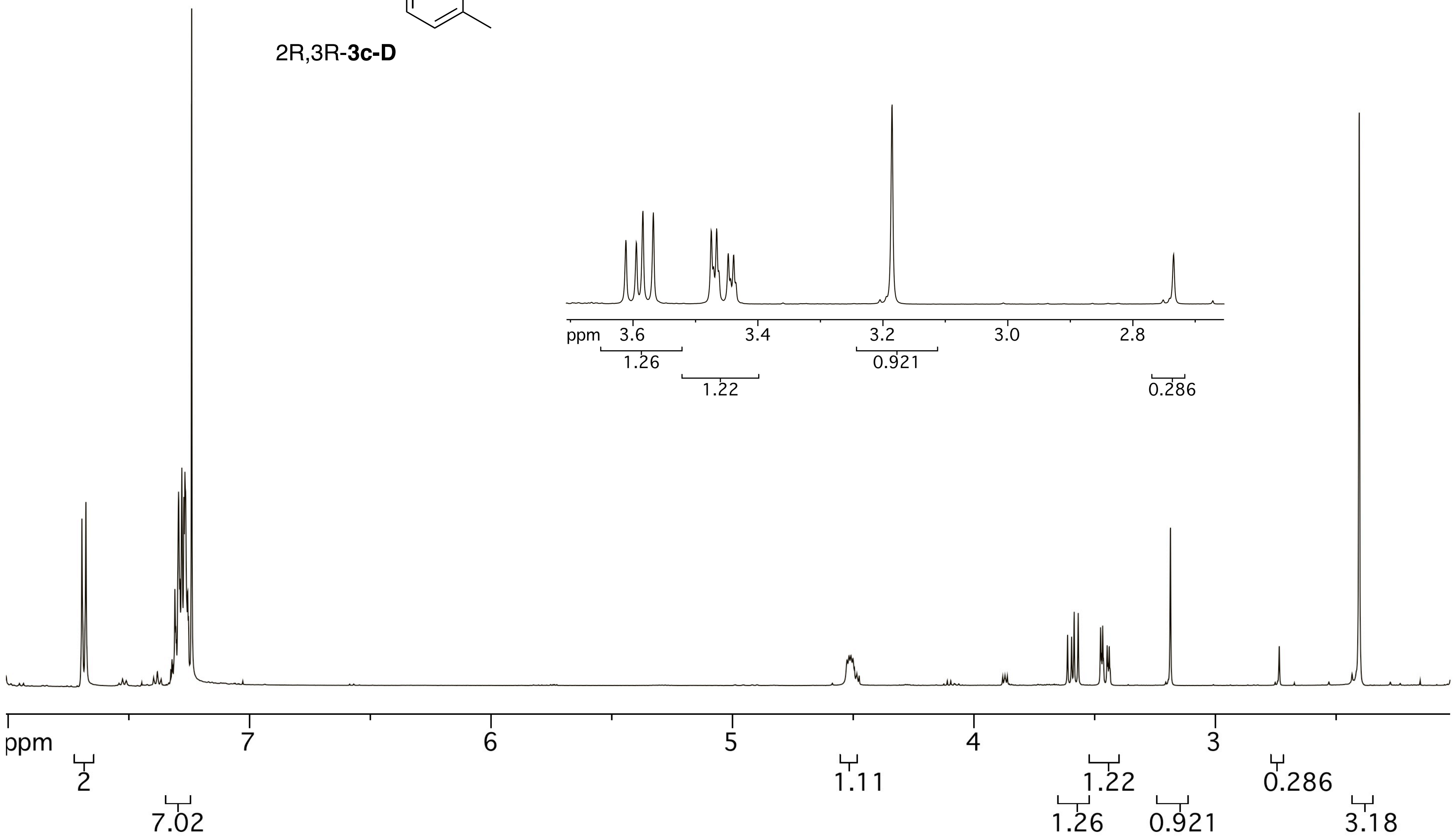
ppm
3.6
3.4
3.2
3.0
2.8
1.26
1.22
0.921
0.286
ppm
7
6
5
4
3
2
7.02
1.11
1.22
1.26
0.921
0.286
3.18

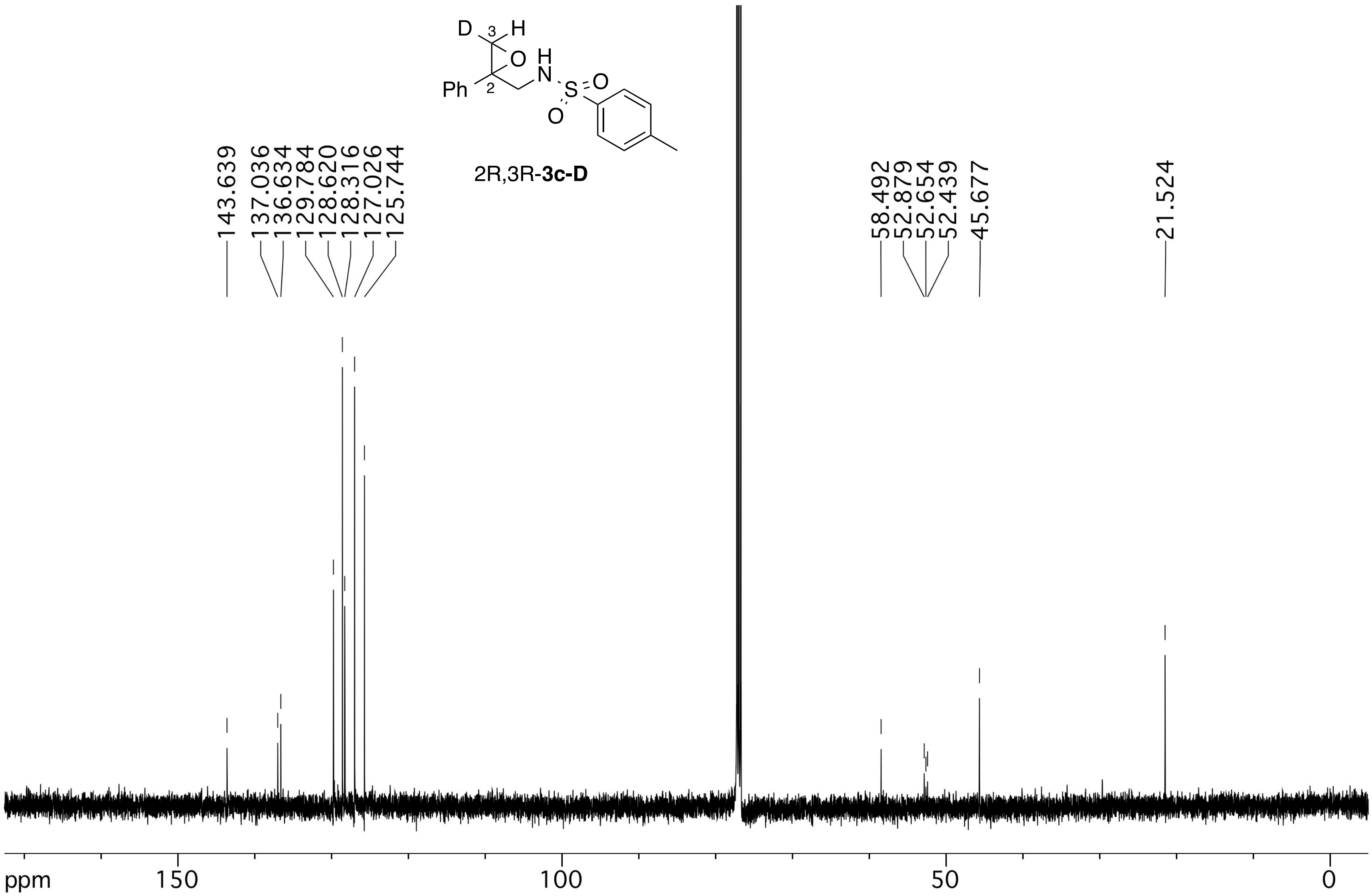

2R,3R-**3c-D**
143.639
137.036
136.634
129.784
128.620
128.316
127.026
125.744
58.492
52.879
52.654
52.439
45.677
21.524
ppm
150
100
50
0

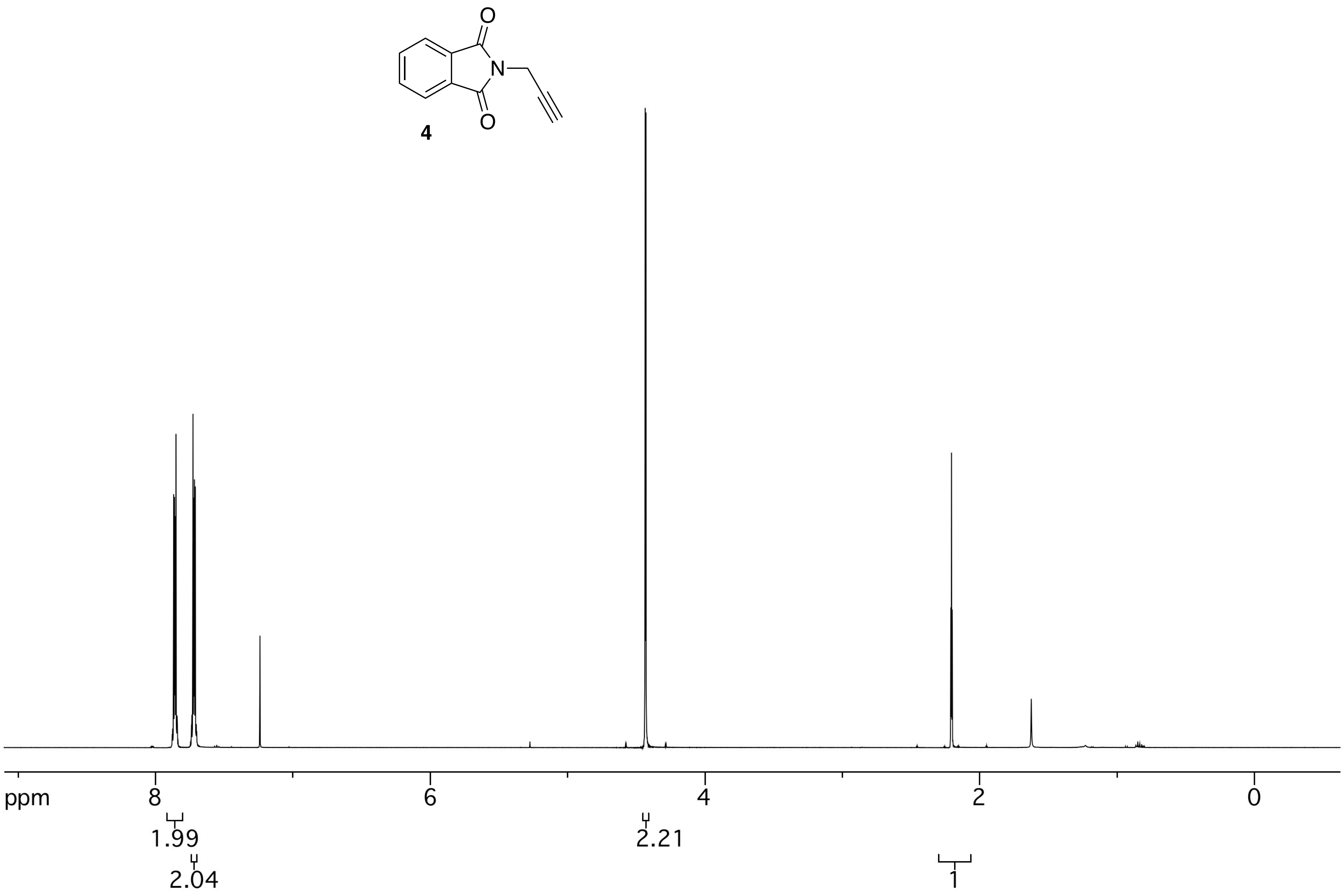

O
N
O
4
ppm
8
6
4
2
0
1.99
2.04
2.21
1

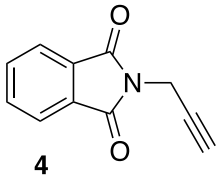

4

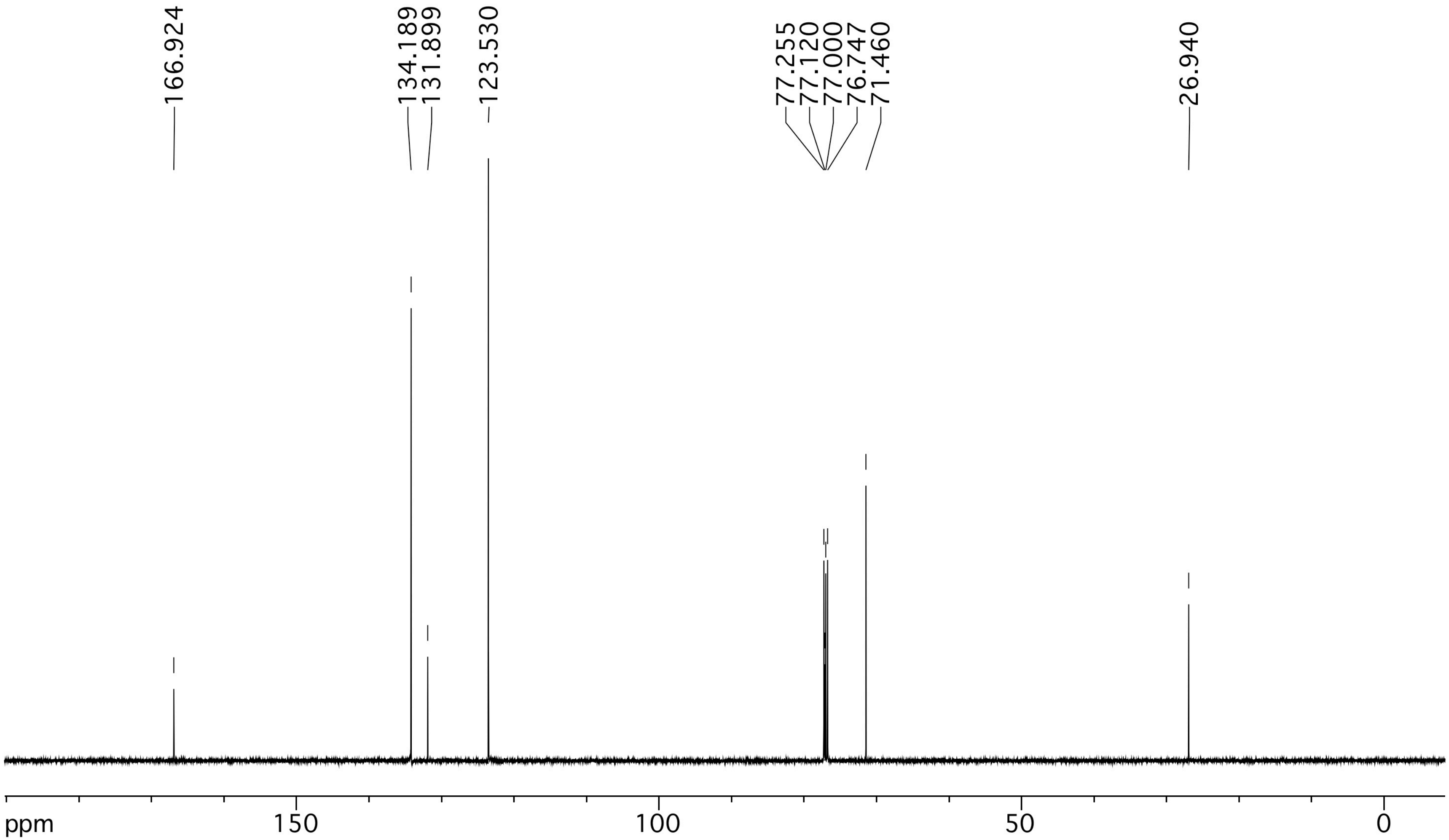
166.924
134.189
131.899
123.530
77.255
77.120
77.000
76.747
71.460
26.940
ppm
150
100
50
0
